# The crystal engineering potential and challenge of dl-malic acid, a chemical chameleon for coordination polymers.

**DOI:** 10.1039/d6ce00715e

**Published:** 2026-10-19

**Authors:** Nicholas B. Page, Michael J. Zaworotko

**Affiliations:** a Department of Chemical Sciences, Bernal Institute, University of Limerick V94 T9PX Republic of Ireland xtal@ul.ie

## Abstract

The conformational flexibility of malate anions, a biomass-derived linker ligand, enables a diverse range of coordination environments that complicates its utility for crystal engineering studies. This work seeks to expand upon our understanding of the malate anion as a linker for the generation of coordination networks. We report six dl-malate coordination compounds that can be classified as 1D coordination polymers, 2D coordination networks or porous hydrogen-bonded frameworks. In addition to analysis of the crystal structures, a CSD mining study was undertaken to examine the coordination behaviour of *gauche*- and *trans*-malate conformers upon the structure of 80 reported coordination polymers that comprise malate anions. Our analysis indicates that, whereas the torsional diversity of malate anions represents a crystal engineering challenge, there are possible crystal engineering strategies for biasing malate linkers to adopt a particular conformer in order to enable this green biomass-derived linker to control a specific structural outcome for coordination polymers.

## Abstract 2

Malate linker ligands coordinate to metal cations in multiple modes, thereby strongly influencing the dimensionality and topology of the resulting coordination polymers.

## Graphical abstract

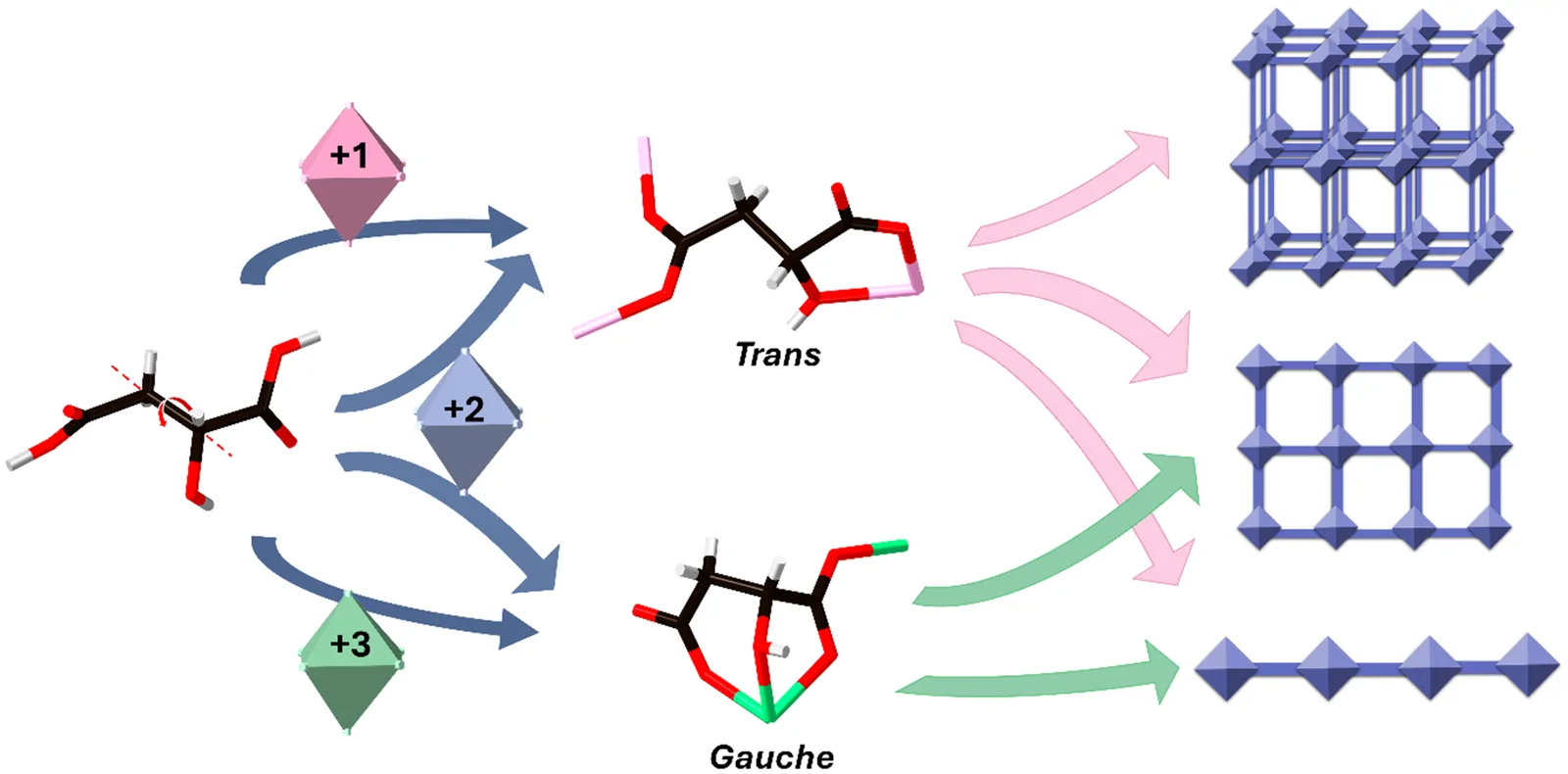

## Introduction

Crystal engineering offers a powerful approach for designing coordination networks (CNs)^1,2^ that exhibit porosity as exemplified by metal–organic frameworks (MOFs)^3,4^ and hybrid ultramicroporous materials (HUMs).^5^ That CNs can be prepared by self-assembly of pre-built building blocks^5–7^ (molecular building blocks, MBBs^8^) means that CNs are inherently modular. Therefore, not only can they be designed from first principles,^9–11^ they can be fine-tuned to enable systematic study of structure–property relationships.^12–15^ That CNs are typically comprised of metal ion (or cluster) nodes and one or more linker ligands (organic and/or inorganic) means that multiple components can be substituted to enable fine-tuning of pore structure and pore chemistry.^16,17^ Two common design elements that can be controlled by ligand selection are (i) the use of bifunctional linker ligands to propagate adjacent metal nodes (metal ions or metal clusters)^6,18–20^ or (ii) exploitation of bridging ligands to facilitate the formation of infinite one-dimensional rod building blocks (RBBs).^17,21,22^ Applying such principles does not mean that specific CN topologies and structures are always predictable. Indeed, this is especially the case for ligands that can serve as a linker and/or a bridge.

Biomass-derived linkers represent a sustainable approach to design and synthesis to CNs as they are renewably sourced, biocompatible and typically cost-effective. Further, they can be obtained as homochiral compounds. Such linkers encompass a broad range of compositions, including amino acids, nucleobases, saccharides, and their metabolites.^23^ Nevertheless, biomass-derived linkers remain generally underexplored relative to their hydrocarbon counterparts.^24^ This disparity arises largely because synthetic linkers with rigid aromatic backbones have been preferred as they tend to offer more control over structural outcomes. In comparison, many biomass-derived linkers possess aliphatic backbones which can adopt multiple (*e.g. trans*, *eclipsed*, *gauche* and *cis*) conformations.^25^ Citrate, thanks to its branched aliphatic backbone with three carboxylates and an α hydroxyl group, offers numerous possible coordination modes with a high degree of chelation, resulting in a tendency to form dense non-porous coordination networks.^26–28^ This can complicate applying crystal engineering principles to these systems.

Aliphatic dicarboxylic acid linkers can be derived from biomass and, with a C4 aliphatic backbone or longer, such linkers can adopt multiple conformers as seen with the unsubstituted backbone of succinate (Fig. S2.1). With two α-hydroxyl groups on the C_2_ and C_3_ carbon atoms of the C4 backbone, the C_2_–C_3_ bond of tartrate is too sterically hindered to freely rotate and is largely limited to the *gauche*-conformer (Fig. S2.2). Malic acid (H_2_mal) sits in the goldilocks zone, thanks to its single α-hydroxyl group, the malate anion (mal) has a strikingly large number of coordination modes while limited to the extremes of the *gauche* or *trans* conformers (Fig. 1).^29^ This makes mal an interesting candidate to investigate its potential for crystal engineering as the conformers that mal adopts fundamentally change its function as a linker and the resulting coordination polymers and networks it can form. Specifically, *trans*-mal tends to serve as a linear linker with two carboxylate moieties separated by an ethylene backbone. Conversely, *gauche*-mal, is predisposed to form more complex coordination structures by connecting adjacent inorganic nodes to afford infinite 1D and 2D metal carboxylate structures. This is due to the positioning of the α-carboxylate, hydroxyl moiety and β-carboxylate of the *gauche*-mal, which enables these donors to adopt a tridentate (κ^3^) coordination mode with metal ions.^30^ This tridentate coordination mode then limits the resulting coordination polymers to 1D RBBs or 2D layer structures as further coordination with *gauche*-mal to form polymeric structures is only available through carboxylates (Fig. 1).

**Fig. 1:**
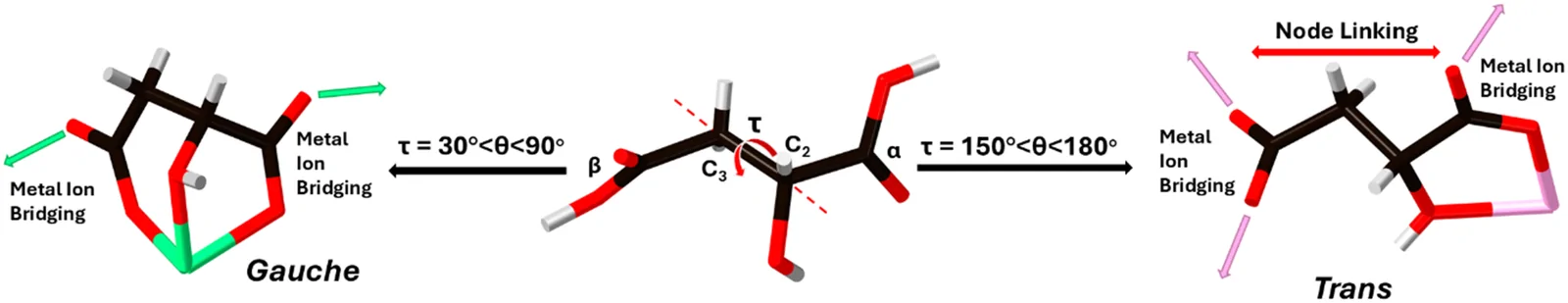
Available coordination modes of the *gauche* (left) and *trans* (right) coordination conformers of malic acid (centre) through the rotation of the C2–C3 bond.

In this study, we report six coordination polymers comprising racemic dl-mal and various divalent metal ions in the presence of hard and soft neutral monotopic ligands such as aqua ligands or benzimidazole (Bzim). By varying the hardness/softness of the metal ion under the same conditions, the resulting structures cover a range of mal conformers and coordination polymers. We also report the results of a Cambridge Structural Database, CSD, v 6.01, May 2026,^31^ database mining study to analyse what might influence the adoption of the *trans*- or *gauche*-conformers and how the conformation of mal in turn affects the resulting coordination networks that form from homochiral d- or l-mal and racemic dl-mal.

## Results and discussion

The conditions used for the synthesis of the mal structures reported herein were adapted from the reported synthesis of [Co_4_(Bzim)_8_(d-mal)_2_(l-mal)_2_]·20H_2_O (SI S1.2).^32^ The synthetic procedure provides hard and soft neutral monotopic ligands from the solvent (water) and/or Bzim to fill the remaining coordination sphere of the metal ion not occupied by mal. Using a range of divalent s-block and d-block metal ions under the same aforementioned synthesis conditions resulted in mal taking on distinct roles in the formation of a variety of coordination complexes (Fig. 2). The harder metal ions were shown to favour water as an ancillary ligand and resulted in distinct 1D and 2D coordination polymers, whereas the softer metal ions favoured Bzim as an ancillary ligand to form isostructural 0D tetranuclear coordination complexes which generate a hydrogen bonded organic framework (HOF^33^). Structures are discussed individually below.

**Fig. 2:**
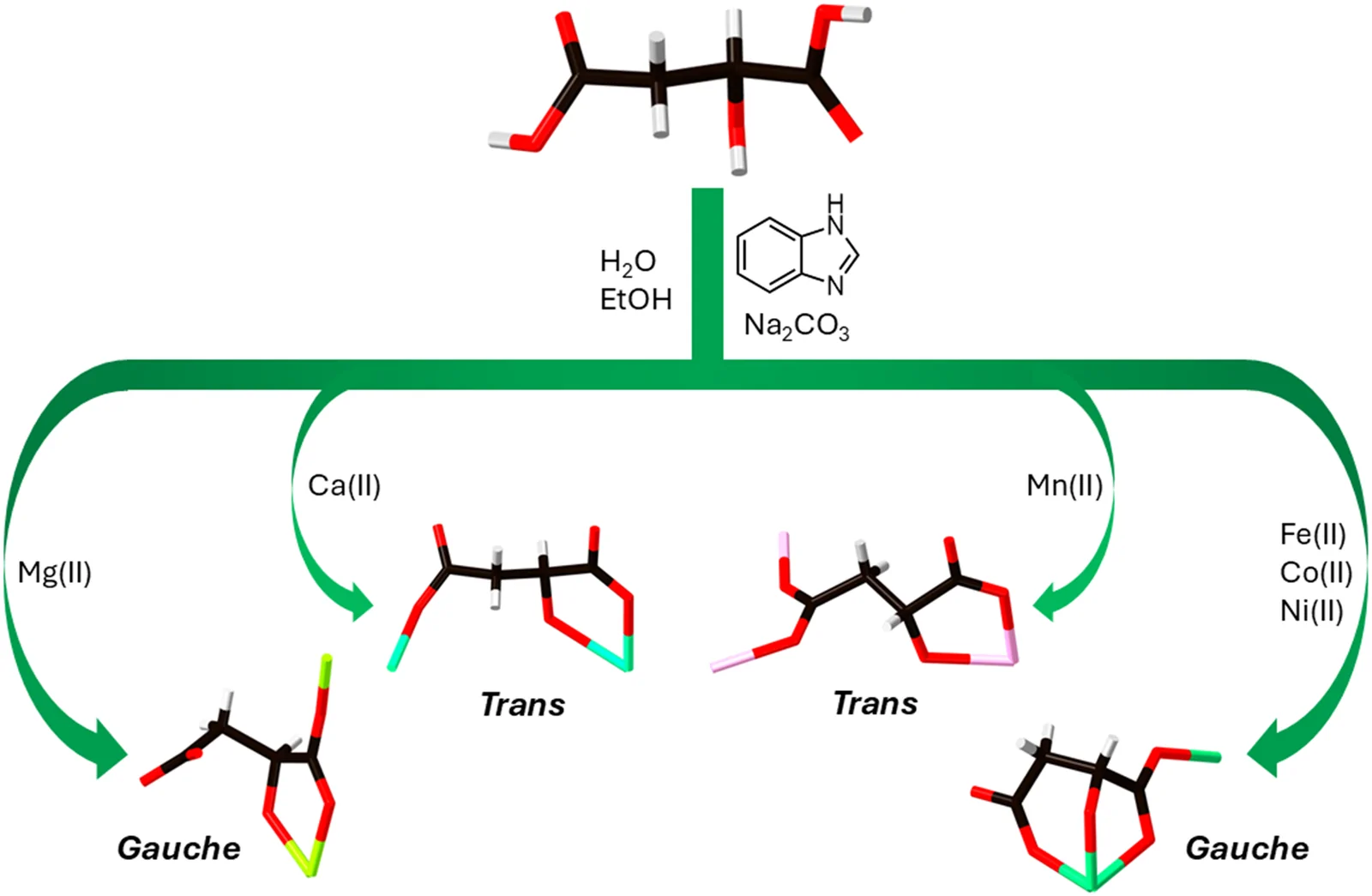
Coordination modes and conformation of the mal ligands in **Mg-Aqua-dl-mal**, **Ca-Aqua-dl-mal**, **Mn-Aqua-dl-mal**, **Fe Bzim-dl-mal**, **Co-Bzim-dl-mal** and **Ni-Bzim-dl-mal**.

### [Mg_2_(H_2_O)_6_(d-mal)(l-mal)] (**Mg-Aqua-dl-mal**)

Single-crystal X-ray diffraction (SCXRD) revealed that **Mg-Aqua-dl-mal** had crystallised in the monoclinic *P*2_1_/*c* space group. Its asymmetric unit is comprised of one Mg(ii) ion, one mal, and three aqua ligands. mal adopts a *gauche* conformation with a C_1_–C_2_–C_3_–C_4_ bond torsion angle of 65.58° and a (κ^2^)-(κ^1^)-μ_2_ coordination mode, connecting adjacent cations through the α-carboxylate hydroxy moiety to form an RBB of alternating d- and l-mal enantiomers (Fig. 3). The remaining coordination sphere of each Mg(ii) ion comprises three aqua ligands. The β-carboxylate does not form any coordination bonds, limiting the dimensionality of the coordination polymer to a 1D RBB structure. The β-carboxylate instead forms a hydrogen bond to an aqua ligand from within the coordination polymer (O⋯O, 2.808(3) Å) and three hydrogen bonds to adjacent coordination polymers, two through aqua ligands (O⋯O, 2.636(3) Å; 2.769 (3) Å) and one to a hydroxyl group (O⋯O, 2.610 (3) Å). The inter-polymer interactions mean that the 1D RBBs face inwards towards the hydrogen-bond-donor aqua ligand to form a bilayer hydrogen-bonding sheet. Thermogravimetric analysis (TGA, Fig. S6.1) reveals a first weight loss event beginning at 110 °C corresponding to 25.5% of its original mass. This is consistent with full dehydration (calculated 25.7%). Two subsequent weight loss events above 285 °C and 450 °C are consistent with thermal decomposition of the mal ligand.

**Fig. 3:**
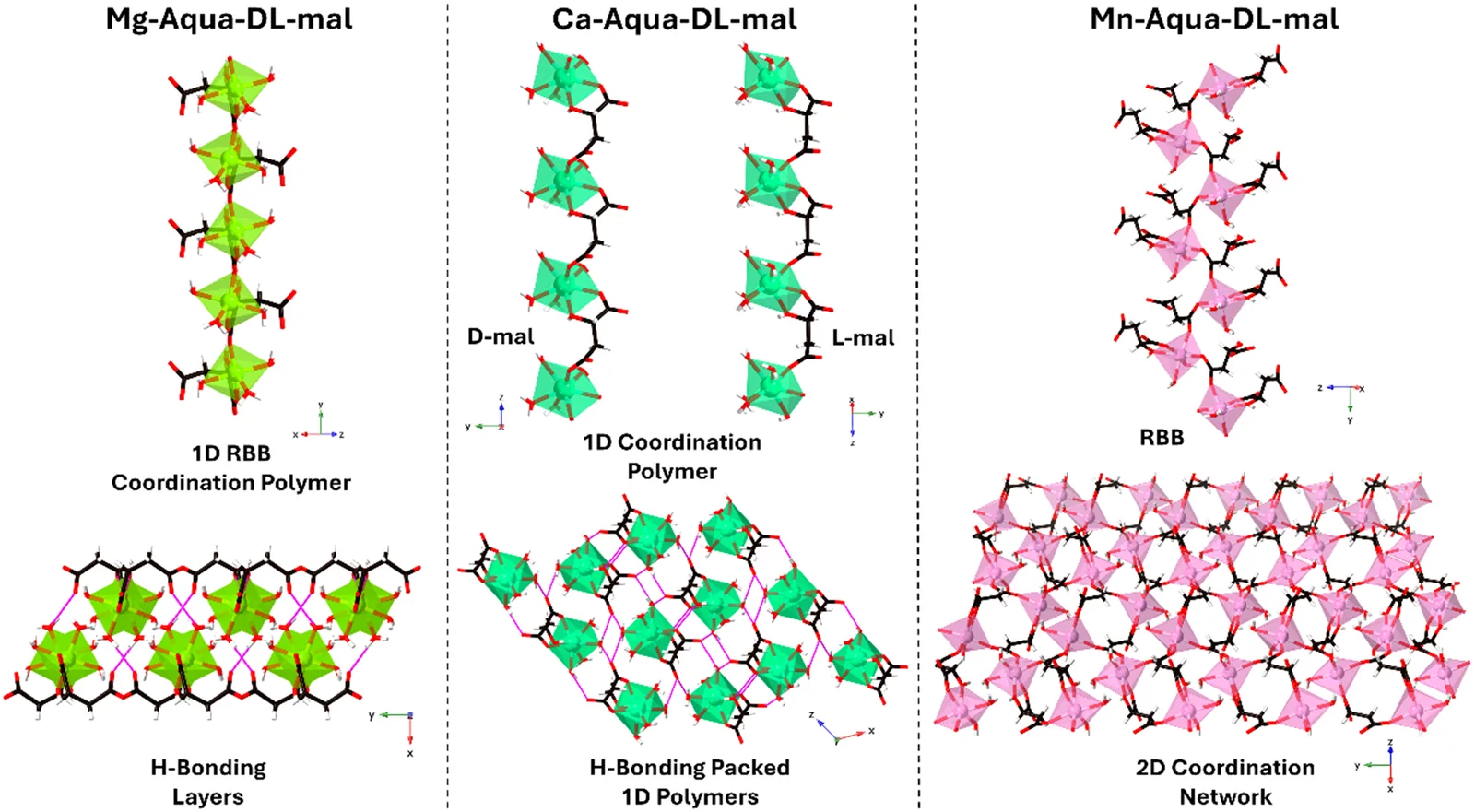
1D RBB structure of the **Mg-Aqua-dl-mal** coordination polymer and hydrogen-bonded layers (left), d-mal and l-mal 1D **Ca-Aqua-dl-mal** coordination polymers and their crystal packing (centre), **Mn-Aqua-dl-mal** RBB and 2D coordination network (right).

### [Ca_2_(H_2_O)_8_(d-mal)(l-mal)] (**Ca-Aqua-dl-mal**)

**Ca-Aqua-dl-mal** crystallised in the monoclinic space group *C*2/*c* with an asymmetric unit comprising one Ca(ii) ion, one mal ligand, and four aqua ligands. This time mal adopted the *trans*-conformation with C_1_–C_2_–C_3_–C_4_ of 168.43° and a (κ^2^)-(κ^1^)-μ_2_ coordination mode, thus serving as a linker. Adjacent cations in **Ca-Aqua-dl-mal** are connected through bidentate α-carboxylate/hydroxy moiety and a monodentate β-carboxylate to generate a 1D coordination polymer (Fig. 3). Each coordination polymer is intrinsically chiral, being comprised from only one mal enantiomer. **Ca-Aqua-dl-mal** crystals are therefore a racemic mixture of the [Ca(d-mal)(H_2_O)_4_] and [Ca(l-mal)(H_2_O)_4_] coordination polymers which are connected by inter-polymer hydrogen bonds between the mal carboxylates and aqua ligands (O_α_⋯O, 2.71(1) Å; O_β_⋯O, 2.77(1) Å). The 1D coordination polymer for the l-mal enantiomer was observed in a previously reported co-crystal with a [Ca(l-tartrate)(H_2_O)_4_] 1D coordination polymer in a 1 : 1 ratio.^34^

### [Mn_2_(H_2_O)_4_(d-mal)(l-mal)]·2H_2_O (**Mn-Aqua-dl-mal**)

**Mn-Aqua-dl-mal** crystallised in the orthorhombic space group *Pbca* with an asymmetric unit comprised of one Mn(ii) ion, one mal, two aqua ligands, and a lattice water molecule of hydration. The crystal structure of **Mn-Aqua-dl-mal** has previously been reported, having been synthesised under a variety of conditions.^35–39^ In addition, two isostructural analogues have been reported using Mg(ii) and Fe(ii) (Refcodes: HILGAO and XIRFIO) being synthesised under different conditions and without the presence of Bzim.^40,41^ The mal anion adopts a *trans*-conformation with a C_1_–C_2_–C_3_–C_4_ torsion angle of 172.47° and a (κ^2^)-(κ^1^)-(κ^1^)-μ_3_ coordination mode and serves as both a linker and a bridge. The β-carboxylate of mal anions bridge adjacent Mn(ii) ions to form a 1D Mn–(COO)–Mn RBB. d- and l-mal pairs are arranged in a head-to-tail fashion. These mal anions link the equivalent Mn(ii) ion of adjacent RBBs to result in a 2D coordination network. The two aqua ligands coordinated to each Mn(ii) ion adopt a *cis*-configuration, with one aqua ligand lying in the plane of the 2D network and hydrogen-bonding to the mal of adjacent 2D sheets (O⋯O, 2.712(4) Å). The TGA thermogram (Fig. S6.2) shows an initial weight loss of 16.2% beginning at 80 °C which is constant with the loss of the lattice water and one aqua ligand (calculated 14.9%). The second weight loss event of 6.1% beginning at 150 °C indicates full dehydration (calculated 7.5%). The further weight loss events occur gradually above 250 °C and 400 °C, respectively, and are consistent with thermal decomposition of the mal ligand.

### [M_4_(Bzim)_8_(d-mal)_2_(l-mal)_2_]·X, **Fe Bzim-dl-mal**, **Co-Bzim-dl-mal**, **Ni-Bzim-dl-mal**

SCXRD revealed that **Fe-Bzim-dl-mal**, **Co-Bzim-dl-mal**, and **Ni-Bzim-dl-mal** are isostructural, each crystallising in the tetragonal *P*4/*n* space group. The asymmetric unit comprises the respective divalent metal ion (Co(ii), Ni(ii), or Fe(ii)), one mal, two Bzim ligands, and lattice solvent. The mal ligand adopts *gauche*-conformation with a C_1_–C_2_–C_3_–C_4_ torsion angle of 61.62°, 61.02°, and 61.65° for the Fe(ii), Co(ii), and Ni(ii) analogues, respectively. The *gauche*-mal acts as a tridentate ligand with the α-carboxylate and hydroxyl group as well as the β-carboxylate coordinating to a metal ion in addition to the β-carboxylate coordinating to a second metal ion in a monodentate fashion resulting in mal having a (κ^3^)-(κ^1^)-μ_2_ coordination mode. The mal connects pairs of metal ions through monodentate coordination with the α-carboxylate. The enantiomers of mal alternate; with this alternation, the tridentate coordination mode caps alternating top and bottom faces of the metal ion and results in the bridging α-carboxylate being angled to form a closed tetranuclear ring cluster. The coordinated Bzim ligands are arranged in a *cis*-fashion, with one Bzim lying in an equatorial position or an alternating axial position on the top or bottom face of the ring. The equatorial Bzim ligands each hydrogen-bond to a separate tetranuclear ring *via* the imidazole hydrogen to the β-carboxylate (Fe, Co, Ni; N⋯O, 2.962(7), 2.862(8), 2.812(7) Å). This hydrogen bond is reciprocated by an equatorial Bzim of the adjacent tetranuclear ring. This hydrogen-bonding motif likely plays a structure-directing role in the formation of the tetranuclear clusters. Considering each tetranuclear ring as a node results in a 2D **sql** hydrogen-bonding framework along the *a* and *b* crystallographic axes. Along the *c* axis, the tetranuclear clusters stack in a linear fashion, with the pairs of axial Bzim interlocked together through edge-to-face π–π interactions. This aligns the void space of the 2D hydrogen-bonding sheets into 1D solvent-accessible channels (Fig. 4).

**Fig. 4:**
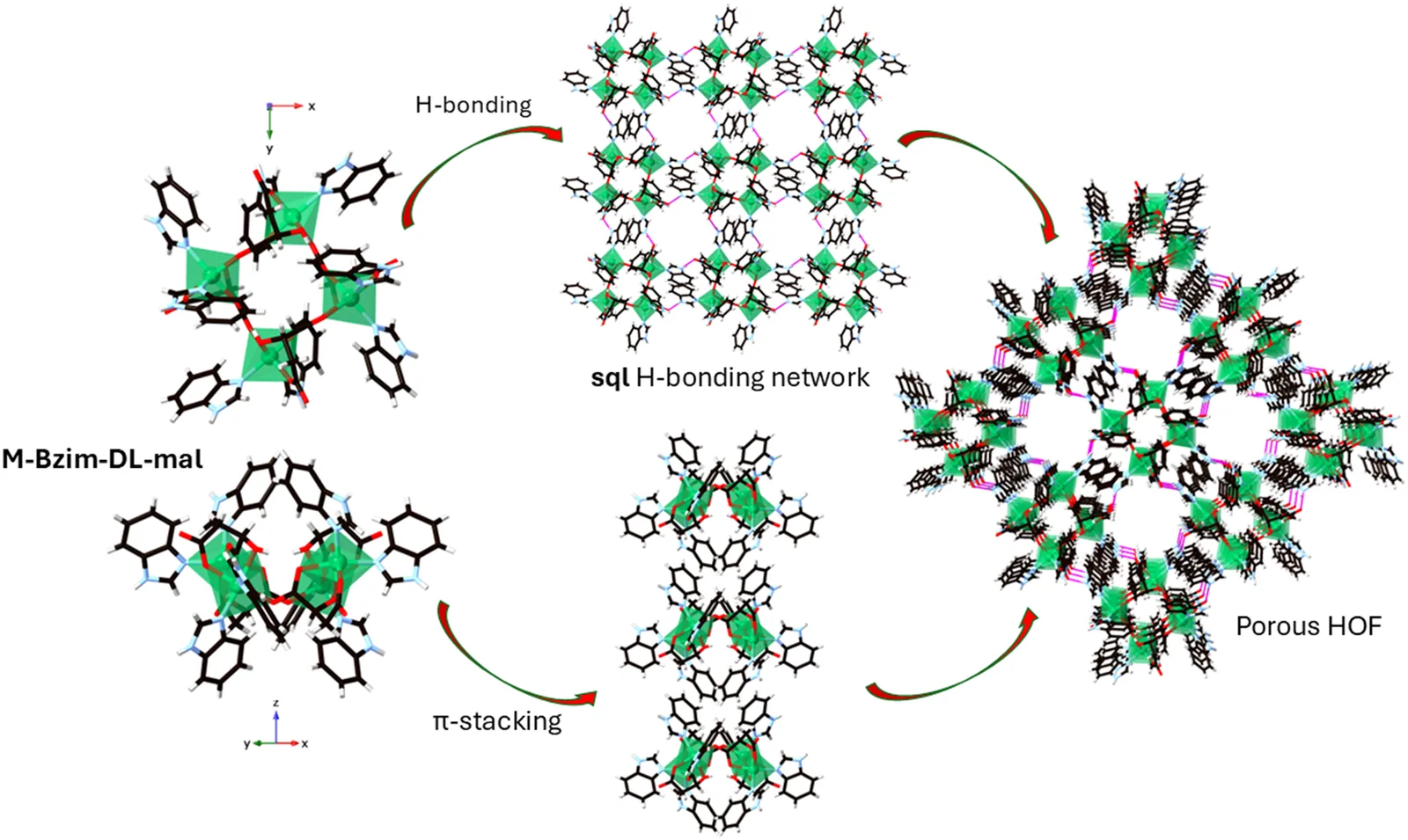
Tetranuclear M-Bzim-dl-mal unit (left) and the network of hydrogen bonding (magenta) and π–π interactions (centre) to arrange into HOF with 1D porous channels.

The crystal structures of the isostructural HOFs differ in their solvation and how the structures were refined. Both **Fe-Bzim-dl-mal** and **Co-Bzim-dl-mal** were able to have solvent modelled in their respective channels. The PLATON SQUEEZE^42^ routine was used on all three structures to deal with the remaining electron density. **Co-Bzim-dl-mal** was found to have 8 lattice water molecules and 4 lattice ethanol molecules modelled in the structure per tetranuclear complex. The SQUEEZE routine removed 60 electrons from the unit cell which we attribute to an additional three water molecules per tetranuclear complex. **Fe-Bzim-dl-mal** was found to have 4 lattice ethanol molecules per tetranuclear complex. The SQUEEZE routine removed 161 electrons from the unit cell which we attribute to an additional lattice ethanol molecule and 5 lattice water molecules per tetranuclear complex. Solvent molecules could not be found in the channels of **Ni-Bzim-dl-mal**. The SQUEEZE routine removed 515 electrons from the unit cell which we attribute to three lattice ethanol molecules and 18 lattice water molecules per tetranuclear complex. The previously reported structure of **Co-Bzim-dl-mal** is a different solvate, with 20 lattice water molecules in the solvent accessible channels of the HOF per tetranuclear complex.^32^ The TGA thermograms of both the **Co-Bzim-dl-mal** and **Fe-Bzim-dl-mal** (Fig. S6.3–S6.4) reveal no discrete events below the final measured temperature of 500 °C indicating gradual solvent removal from the channels before thermal decomposition. The TGA thermogram of **Ni-Bzim-dl-mal** (Fig. S6.5) reveals 8.5% weight loss by 100 °C after which mass loss plateaus until 210 °C where an onset of constant weight loss begins. The discrepancy between the expected weight loss of 26.2% with the complete removal of the solvate and the 8.5% weight loss observed can be accounted for as the onset begins at room temperature before being recorded on the TGA thermogram.

As **Ni-Bzim-dl-mal** has the potential to remain porous upon removal of the solvent occupying the channels it was investigated for its sorption properties. To prepare the material approximately 100 mg was solvent exchanged with dry methanol (10 mL) over the course of 3 days, with the solvent being replaced with fresh methanol every 24 hours. Variable temperature powder X-ray diffraction (VT-PXRD) revealed retention of the crystalline phase of **Ni-Bzim-dl-mal** up to 80 °C where a phase change occurred consistent with loss of crystallinity. As such an activation temperature of 60 °C was chosen for both water vapor and gas adsorption measurements. The adsorption isotherm of **Ni-Bzim-dl-mal** indicates no uptake of N_2_ at 77 K (Fig. S9.1). However, the 195 K CO_2_ and C_2_H_2_ isotherms (Fig. S9.2) reveal uptake of 15.3 cm^3^ g^−1^ and 13.0 cm^3^ g^−1^ with a type II and III adsorption isotherms, respectively. Subsequent 273 K and 298 K CO_2_ isotherms reveal uptakes of 10.8 cm^3^ g^−1^ and 8.2 cm^3^ g^−1^, respectively. The water sorption isotherm collected at 298 K (Fig. S10.1) reveals uptake of 12.06 wt% with large hysteresis during desorption. Full desorption was not achieved, leaving 2.75 wt% of water at 0% relative humidity (RH).

## CSD analysis of mal coordination compounds

Beyond the structures of the mal compounds discussed above, mining of the MOF subset of the Cambridge Structural Database (CSD) was conducted to provide insight into the role of *gauche-* and *trans*-mal conformers upon resulting coordination networks.^43^ In the MOF subset of the CSD,^44^ there are 80 unique mal-containing structures, of which 45 (56.3%) are homochiral (Fig. 5), employing enantiopure mal to explore the bulk optical, magnetic and separation capabilities of these materials.^45^ In addition, from these 80 reported mal coordination polymers, there are 97 crystallographically unique mal fragments. As with the structures reported above, the conformation of these mal anions can be determined by the C_1_–C_2_–C_3_–C_4_ bond torsion angle. Analysis of the mal torsion angles revealed, as expected, two groups: 43 mal anions (44.3%) in the *gauche*-conformation, and 52 (53.6%) in the *trans*-conformation. There are two structures (2.1%) with mal linkers having torsion angles just outside the expected *trans* range (180–150°), at 144.805° and 147.368° (Refcodes: FODZII, PACLIP).^46,47^ The *eclipsed* conformation in these coordination complexes could be due to steric effects and hydrogen bonding interactions with the adjacent coordination polymers in the structures.

**Fig. 5:**
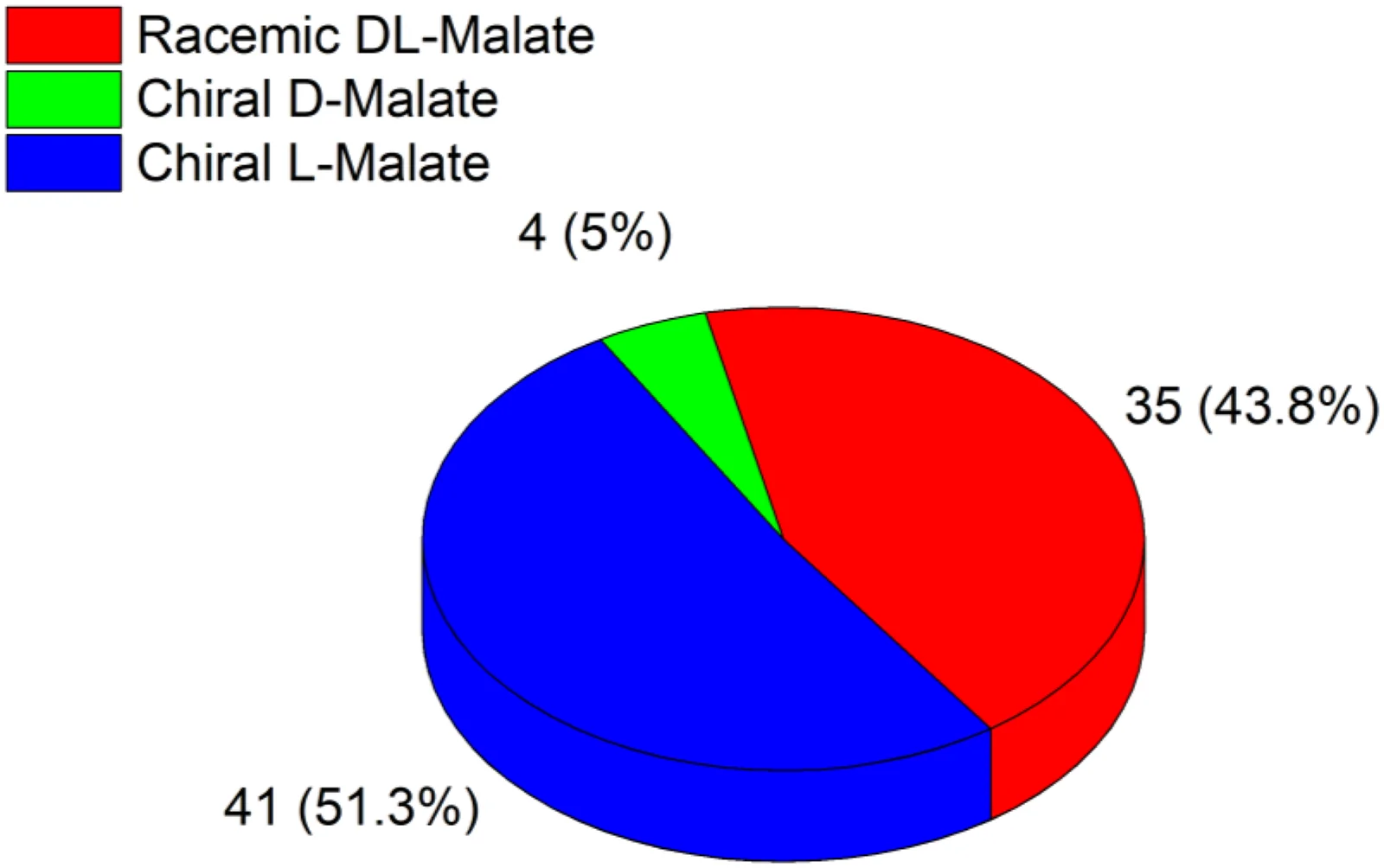
Number and proportion of reported malate coordination polymers employing homochiral d-malate, l-malate or a racemic dl-malate mixture.

To determine whether the α- and β-carboxylate moieties chelate to the same metal ion, the distance between the β-carboxylate oxygen atoms and metal ions coordinated to the α-carboxylate was examined. This reveals that 10 *gauche*-mal structures do not adopt the tridentate chelate coordination mode; instead, these linkers serve as bent linkers. **Mg-Aqua-dl-mal** is a new example of this type, with mal adopting the *gauche* conformer for the β-carboxylate to hydrogen-bond to aqua ligands instead of connecting RBBs.

Further analysis of these structures revealed that, for the *gauche*-conformer, owing to its tridentate coordination mode, it is restricted to forming 1D RBBs and 2D layer structures as all coordinated metal ions are connected through carboxylate moieties. For divalent metals, *gauche*-mal anions form RBB structures through monodentate coordination by the β-carboxylate. As shown in Fig. 7, these RBBs have two *cis*-coordination sites available for secondary linkers to connect other RBBs, with the alternating position allowing for the secondary linker to connect four RBBs and form a 3D network. The use of d-, l- or dl-mal has little effect on the RBB structure as each enables the same connectivity by other linkers. The *gauche*-malates can also form 2D layer structures by cross linking of RBBs by the α-carboxylate into **sql** networks. These sheets have one available coordination on the metal ion for pillaring that alternates its position on the top and bottom face of the 2D layer. For d- or l-mal the pillared layers have **sql** topology. However, using dl-mal distorts the 2D layer structure and, while the sheets retain **sql** topology, the pillars are offset at alternating angles, changing the topology of the resulting CN from **sqp** to **nov** (Fig. 7).

Notably, there is only one reported example of a *gauche*-mal (Refcode: FAYXEJ-02)^47,48^ sustaining a 3D coordination network which requires the use DL-malate to achieve this level of connectivity. The network is assembled from layers of parallel 1D RBBs where the carboxylate moieties coordinate to the RBBs positioned above, with each enantiomer coordinating to a separate RBB to afford the observed 3D structure. As the *trans*-mal conformer in effect separates the α- and β-carboxylates, it tends to function as a linear dicarboxylate linker. Consequently, compared to the *gauche*-conformation, it more readily forms 2D and 3D networks and offers greater structural diversity. As such, the use of dl-mal over d- or l-mal does not provide access to 3D CNs. However, using dl-mal greatly reduces the likelihood of 1D coordination polymers, with only 15.4% *trans*-mal structures reported use dl-mal as a linker. Thus, like its *gauche*-mal counterparts, d- or l-*trans*-mal has a propensity to form RBB structures, either through α-carboxylate and α-hydroxyl groups or through the β-carboxylate moiety, although *trans*-mal overall is more likely to form CNs even without secondary linkers.^38,49–51^ This difference in function as linker means that if control over which conformation mal adopts can be exerted then it should allow for the crystal engineering of mal coordination polymers.

Analysing the data of our CSD mining reveals two possible strategies to bias which conformation mal adopts. The first is the use of ancillary ligands to occupy part of the metal ion's coordination sphere, thereby preventing mal from tridentate coordination, either due to unavailable coordination sites or through steric interactions between ligands around the metal ion.^46,47,54,55^ The second strategy involves the type of metal ion. The CSD survey reveals that there are no reported *gauche*-mal complexes with monovalent metal ions. In addition, there is only one reported *trans*-mal complex with a trivalent metal ion (Refcode: WOYBAQ).^56^ Conversely, divalent metal ions exist with both *trans*- and *gauche*-conformers and, as shown in Fig. 6, divalent transition metals, particularly Co(ii) and Ni(ii), favour *gauche*-mal, accounting for 60% and 92.9% of the reported complexes, respectively. Harder s-block divalent metal ions favour *trans*-mal complexes, accounting for 100% and 66.6% of reported Mg(ii) and Ca(ii) complexes, respectively. A similar trend is also observed in the dl-mal crystal structures reported herein, with the transition metal Fe(ii), Co(ii) and Ni(ii) cations adopting *gauche*-mal to form tetranuclear complexes while s-block Mg(ii) and Ca(ii) as well as Mn(ii) cations tend to favour *trans*-mal. Welter *et al.* also reported comparable behaviour in their synthesis of *gauche*- or *trans*-mal coordination polymers with Co(ii) and Ni(ii) or Mn(ii) and Fe(ii) metal ions, respectively, under the same hydrothermal conditions.^38^

**Fig. 6:**
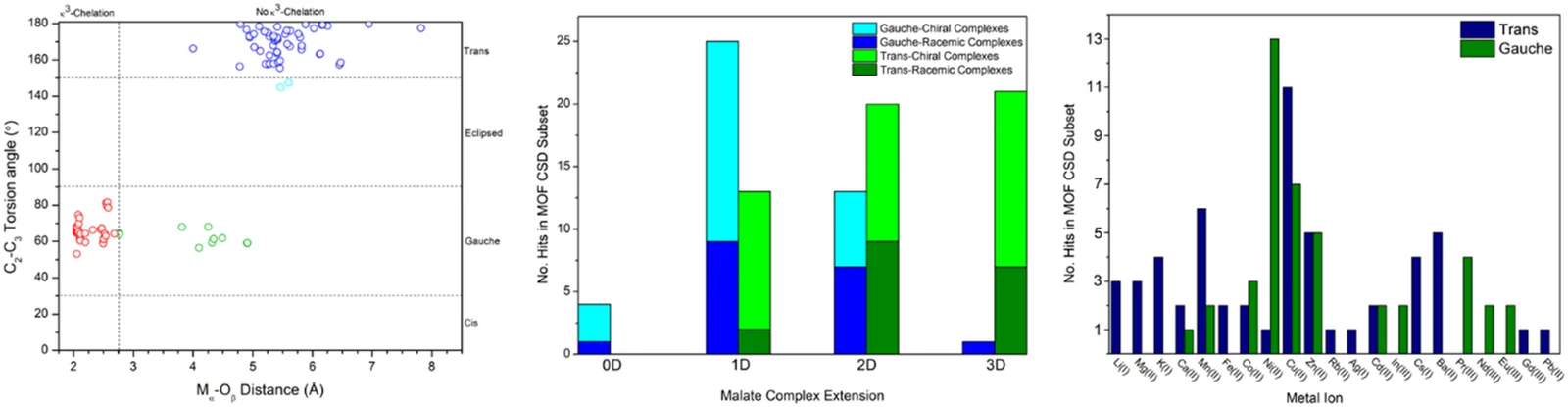
Scatter plot of C1–C2–C3–C4 bond torsion angle and α-coordinated metal ion and β carboxylate distance (left), histogram of malate complex dimension extension *gauche* and *trans* conformers and the proportion that used chiral or racemic malate (centre), histogram of metal ions used for the formation of coordination complexes with *gauche* and *trans* conformer malate (right).

**Fig. 7:**
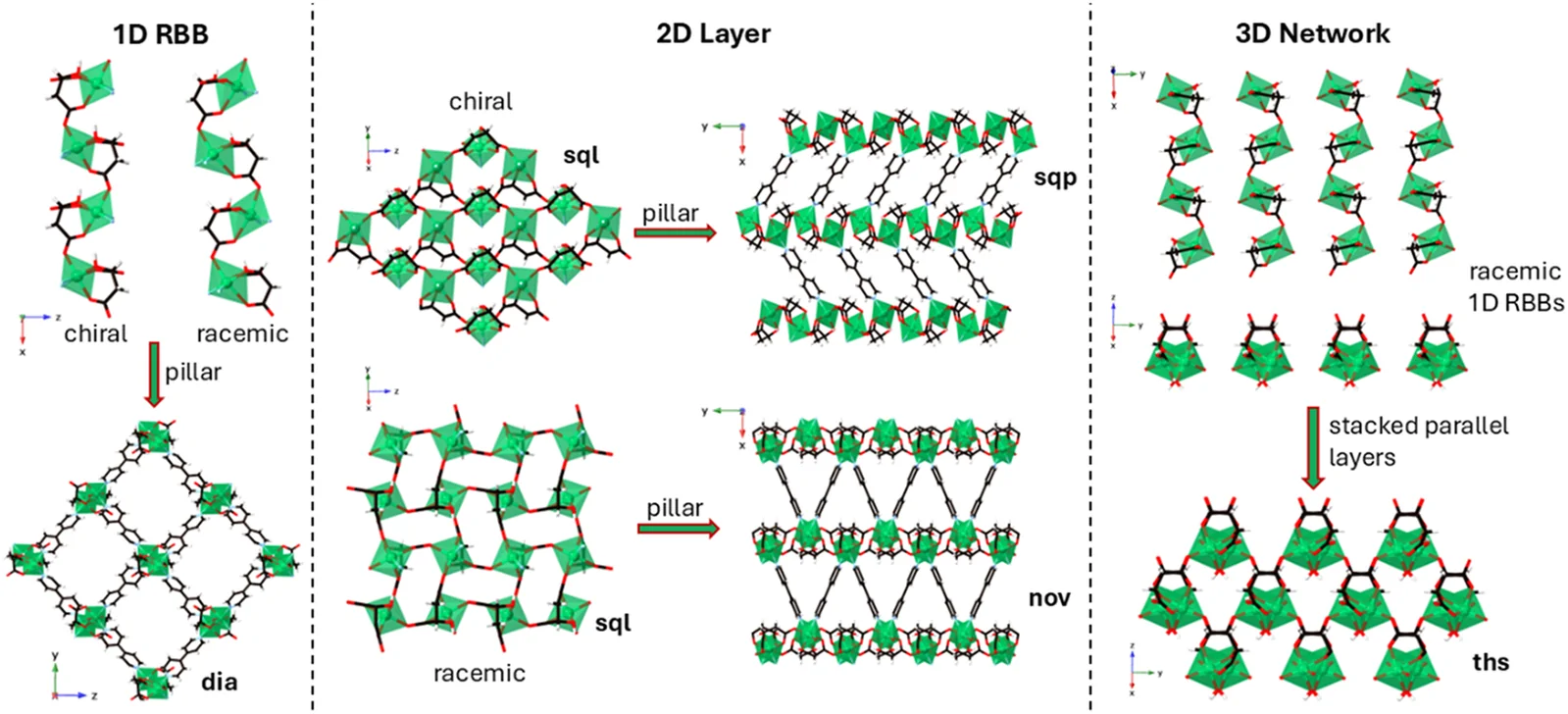
*Gauche*-malate, racemic and chiral 1D RBB Ni(ii) structures, and the resulting **dia** framework from linked RBBs (left),^52^ chiral and racemic *gauche*-malate 2D layer Ni(ii) structures and the resulting pillared frameworks (centre),^53^ 3D Ni(ii) coordination network built from racemic *gauche* malate RBBs (right).^47,48^

## Conclusions

The crystal structures reported herein, and the CSD survey of mal comprising coordination polymers add to our understanding of the ability of mal to serve as a linker in coordination polymers. This study provides three crystal engineering insights: (i) the *gauche*-conformer reliably forms RBB or 2D layer structures that can be further connected by secondary linkers whereas the *trans*-conformer tends to form 3D networks; (ii) the choice of using homochiral or racemic mixtures of mal can be used to target the dimensionality of the resulting coordination polymer, but as only chiral and racemic mal complexes are reported in the MOF subset of the CSD, further study could address how altering the enantiomeric excess beyond a racemic mixture could affect the dimensionality of resulting coordination polymers and reveal further crystal engineering strategies (iii) the chemistry of the metal ion chosen for coordination by the mal linker appears to bias it to adopt either its *trans*- or *gauche*-conformer; monovalent and possibly trivalent metal ions favour *trans*-mal whereas divalent cations allow both conformer, but some divalent ions such as Ni(ii) could bias towards formation of *gauche*-mal. This research therefore suggests that some degree of control over mal anions can be exercised while using it as a sustainable biomass-derived linker ligand.

## Author contributions

Nicholas B. Page: material preparation, investigation, formal analysis, writing – original draft; Michael J. Zaworotko: supervision, funding acquisition, writing – review & editing.

## Conflicts of interest

There are no conflicts to declare.

## Supplementary Material

CE-028-D6CE00715E-s001

## Data Availability

The data supporting the findings of this study are available in the supplementary information (SI) or from the authors upon request. Supplementary information: synthesis details, CSD Survey, crystallographic data, thermogravimetric analysis data, differential scanning calorimetry data, FT-IR data, adsorption measurements. See DOI: https://doi.org/10.1039/d6ce00715e. CCDC 2580112–2580117 contain the supplementary crystallographic data for this paper.^57*a*–*f*^
