# Supplementary material for: The crystal engineering potential and challenge of dl-malic acid, a chemical chameleon for coordination polymers
Source: CrystEngComm. 2026 Oct 19. Online ahead of print. doi: 10.1039/d6ce00715e (PMC13712407; doi:10.1039/d6ce00715e)
Supplement: Supplementary file 1 [file CE-OLF-D6CE00715E-s001.pdf]

Supplementary Information (SI) for CrystEngComm.
This journal is © The Royal Society of Chemistry

Supplementary Information

The crystal engineering potential of malic acid; a sustainable chameleon linker for varied coordination polymers.

Nicholas B. Page [a] and Michael J. Zaworotko*[a]

[a]Department of Chemical Sciences, Bernal Institute, University of Limerick, V94 T9PX, Republic of Ireland

E-mail: xtal@ul.ie

Table of Contents

S1. General experimental 2
S2 CSD Survey 2
S4.1 Crystallographic Tables 34
S4. Powder X-ray Diffraction 35
S5. Variable Temperature PXRD 38
S6. Thermogravimetric analysis 39
S7. Differential Scanning Calorimetry (DSC) 42
S8. Fourier transform infrared 45
S9. Gas Adsorption 48
S10. Water Vapor Sorption Isotherms 49
S11. References 49

S1. General experimental

S1.1 Materials

All reagents and solvents were used as received from commercial sources without further purification.

S1.2 Synthesis

All reagents and solvents are commercially available and were used without further purification. As detailed below, all six MOM were prepared following the same synthesis procedure using a method adapted for the reported synthesis of **[$Co_4(Bzim)_8$(D-mal)$_2$(L-mal)$_2$]·20H_2O.**[1]

Synthesis of [$Mg_2(H_2O)_6$(D-mal)(L-mal)] (Mg-Aqua-DL-mal). $Mg(NO_3)_2·6H_2O$ (544.8 mg, 2.125 mmol), DL-malic acid (286.3 mg, 2.135 mmol) and Na_2CO_3 (245.8 mg, 2.319 mmol) were dissolved in 4 mL DI H_2O and Bzim (508.1 mg, 4.301 mmol) was dissolved in 5 mL EtOH. The two solutions were mixed and left to stand for two days to yield colourless crystals of **Mg-Aqua-DL-mal** (72.9 mg, 59% based on $Mg(NO_3)_2·6H_2O$).

Synthesis of [$Ca_2(H_2O)_8$(D-mal)(L-mal)] (Ca-Aqua-DL-mal). $Ca(OAc)_2·H_2O$ (250.0 mg, 1.419 mmol), DL-malic acid (134.3 mg, 1.002 mmol) and Na_2CO_3 (102.1 mg, 0.963 mmol) were dissolved in 10 mL DI H_2O and Bzim (248.5 mg, 2.104 mmol) was dissolved in 5 mL EtOH. The two solutions were mixed and left to stand for one day to yield a mixture of colourless crystals of **Ca-Aqua-DL-mal** along with uncharacterised microcrystalline and amorphous materials as revealed by the powder X-ray diffractogram of the bulk material (Fig. S4.2). This limited our ability to further analyse the bulk solid.

Synthesis of [$Mn_2(H_2O)_4$(D-mal)(L-mal)]·2H_2O (Mn-Aqua-DL-mal). $Mn(OAc)_2·4H_2O$ (238.8 mg, 0.974 mmol), DL-malic acid (132.7 mg, 0.989 mmol) and Na_2CO_3 (100.7 mg, 0.950 mmol) were dissolved in 10 mL DI H_2O and Bzim (241.4 mg, 2.043 mmol) was dissolved in 5 mL EtOH. The solutions were mixed and left to stand for two days to yield pink crystals of **Mn-Aqua-DL-mal** (156.0 mg, 67% based on $Mn(OAc)_2·4H_2O$).

Synthesis of [$Fe_4(Bzim)_8$(D-mal)$_2$(L-mal)$_2$]·4EtOH (Fe-Bzim-DL-mal). $FeSO_4·7H_2O$ (584.5 mg, 2.102 mmol), DL-malic acid (265.0 mg, 1.976 mmol) and Na_2CO_3 (202.8 mg, 1.913 mmol) were dissolved in 10 mL DI H_2O and Bzim (481.6 mg, 4.077 mmol) was dissolved in 5 mL EtOH. The two solutions were mixed and left to stand for two days to yield yellow crystals of **Fe- Bzim-DL-mal** (678.3 mg, 73% based on DL-malic acid). Note: the production of CO_2 by the Na_2CO_3 was used to create a low oxygen environment in the synthesis vial to prevent the oxidation of the Fe(II) ions.

Synthesis of [$Co_4(Bzim)_8$(D-mal)$_2$(L-mal)$_2$]·8H_2O·4EtOH (Co-Bzim-DL-mal). $Co(OAc)_2·4H_2O$ (255.3 mg, 1.025 mmol), DL-malic acid (135.3 mg, 1.009 mmol) and Na_2CO_3 (103.0 mg, 0.97 mmol) were dissolved in 10 mL DI H_2O and Bzim (242.0 mg, 2.048 mmol) was dissolved in 5 mL EtOH. The two solutions were mixed and left to stand for two days to yield purple crystals of **Co-Bzim-DL-mal** (289.1 mg, 59% based on DL-malic acid).

Synthesis of [$Ni_4(Bzim)_8$(D-mal)$_2$(L-mal)$_2$]·16H_2O (Ni-Bzim-DL-mal). $Ni(OAc)_2·4H_2O$ (276.6 mg, 1.564 mmol), DL-malic acid (136.8 mg, 1.020 mmol) and Na_2CO_3 (106.1 mg, 1.001 mmol) were dissolved in 10 mL DI H_2O and Bzim (249.3 mg, 2.110 mmol) was dissolved in 5 mL EtOH. The two solutions were mixed and left to stand for two days to yield green crystals of **Ni-Bzim -DL-mal** (324.5 mg, 64% based on DL-malic acid)

S2 CSD Survey

The Cambridge Structural Database[2] (CSD, v.6.01; database updates: April 2026) was accessed using ConQuest.[3] Crystal structure models were visualized using Mercury and images were created using the Mercury for SI figures and CrystalMaker programs for paper figures.[4]

Table S2.1: Summery of the space group, chemical composition, inorganic structure, malate coordination mode, malate complex structure, and secondary linkers, as reported in the literature.

CSD Code and Reference	Space Group	Formula	Inorganic Structure	Malate-Fragment Enantiomer	C_2–C_3 Torsion Angle (°)	Coordination Mode	Malate Connectivity Extension	Secondary-Linker/ Full Connectivity
ANIYUS[5]	*P*2$_1$	[Cu(II)(L-Hmal)$_2$]·2H_2O	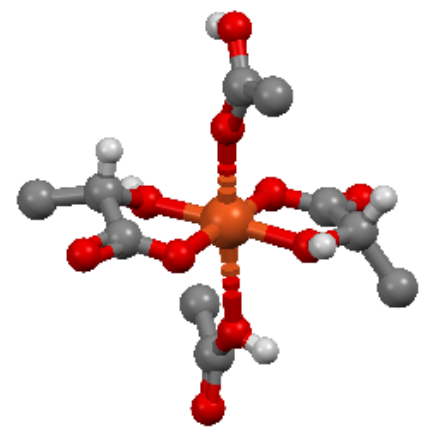 **Mononuclear**	L-Hmal:1	164.31	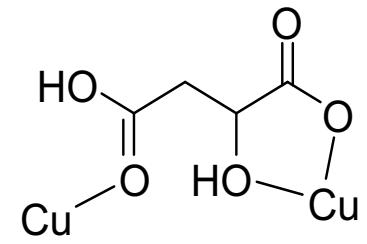 (κ^2)-(κ^1)-μ$_2$	**2D Sheet**	N/A
				L-Hmal:2	172.273	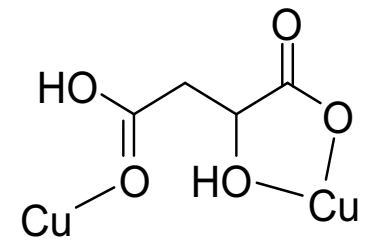 (κ^2)-(κ^1)-μ$_2$		
AYOVES[6]	*P*6$_5$	[Cu(II)$_2$(D-mal)$_2$(4,4'bipy)H_2O]·3H_2O	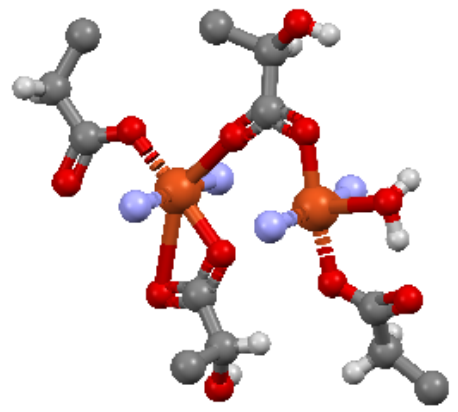 **Dinuclear**	D-mal:1	59.142	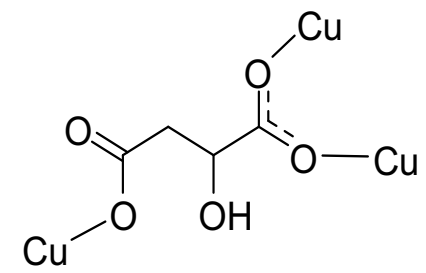 (κ^1) -(κ^1) -(κ^1)-μ$_3$	**1D Polymer**	4,4'-bipyridine 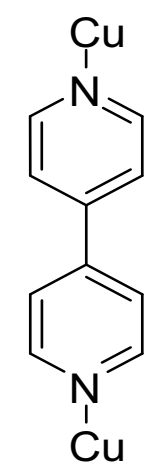 **3D Network**
				D-mal:2	157.366	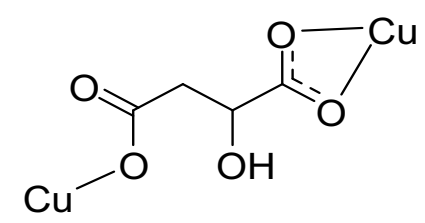 (κ^2)-(κ^1)-μ$_2$		
AYUCOP[6]	*P*6$_1$	[Cu(II)$_2$(L-mal)$_2$(4,4'bipy)H_2O]·3H_2O	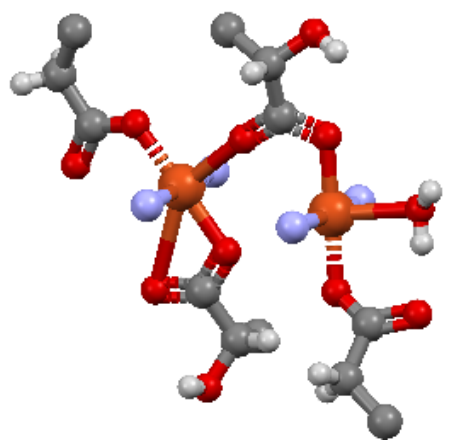 **Dinuclear**	L-mal:1	59.009	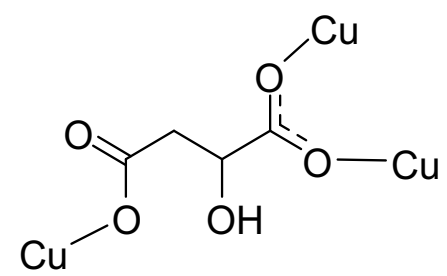 (κ^1) -(κ^1) -(κ^1)-μ$_3$	**1D Polymer**	4,4'-bipyridine 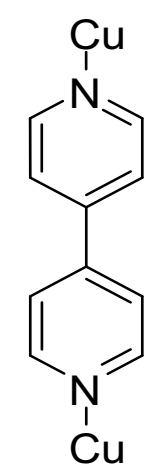 **3D Network**
				L-mal:2	158.578	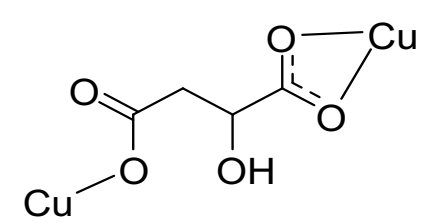 (κ^2)-(κ^1)-μ$_2$		

BAFCES[7]	*Pbca*	[Cd(II)(DL-mal)H_2O]	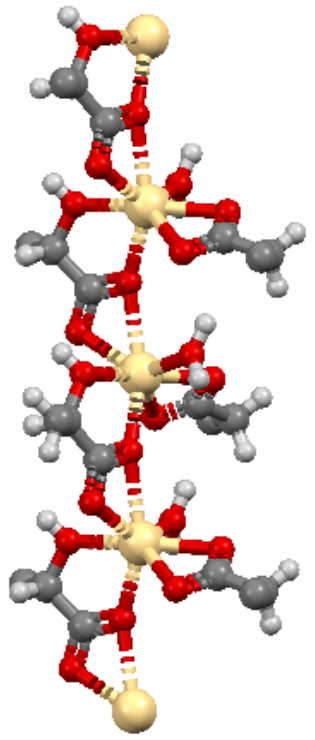 **1D RBB**	DL-mal:1	68.085	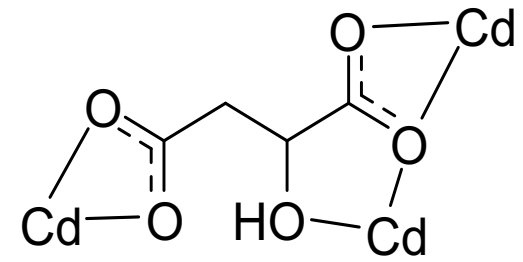 (κ^2) -(κ^2)-(κ^2)-μ_3	**2D Sheet**	N/A
BESXED[8]	*P*2$_1$	[Cs(I)(L-mal)H_2O]	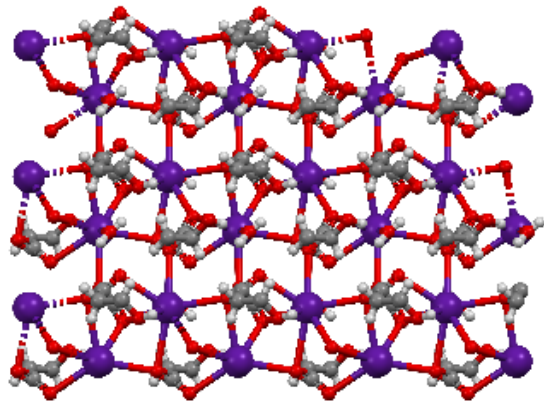 **2D Layer**	L-mal:1	166.271	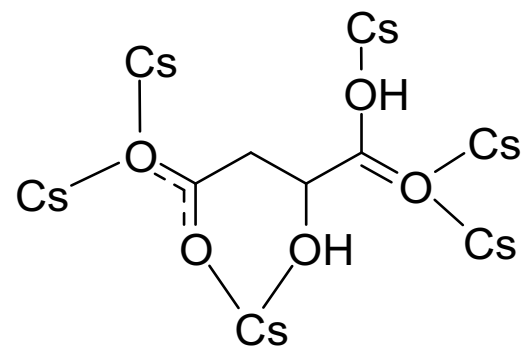 (κ^1)- (κ^1)-(κ^1)-(κ^2)-(κ^1)-(κ^1)-μ_6	**3D Network**	N/A
BESXIH[8]	*P*2$_1$	[Cu(II)(L-mal)$_2$]	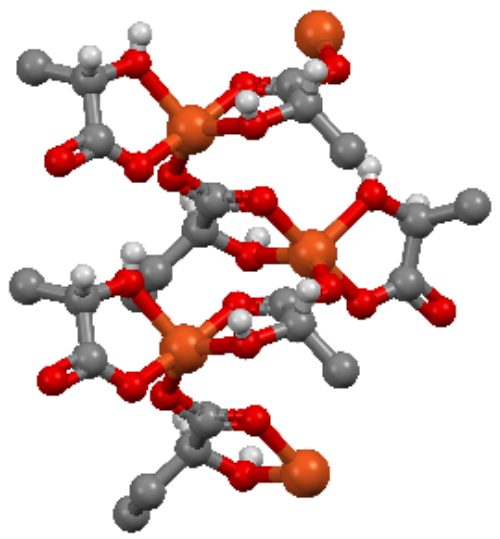 **1D RBB**	L-mal:1	172.736	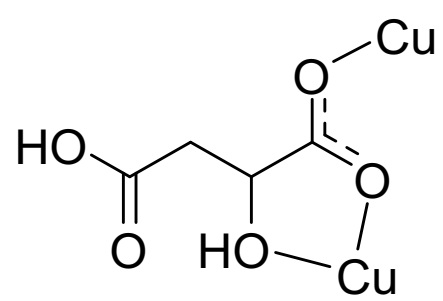 (κ^2)-(κ^1)-μ_2	**1D Polymer**	N/A
				L-mal:2	67.983	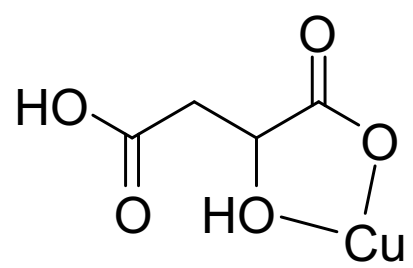 κ^2		

BUHNUN[9]	*Pcab*	[Mn(II)(DL-mal)$(H_2O)_2$]$\cdot H_2O$	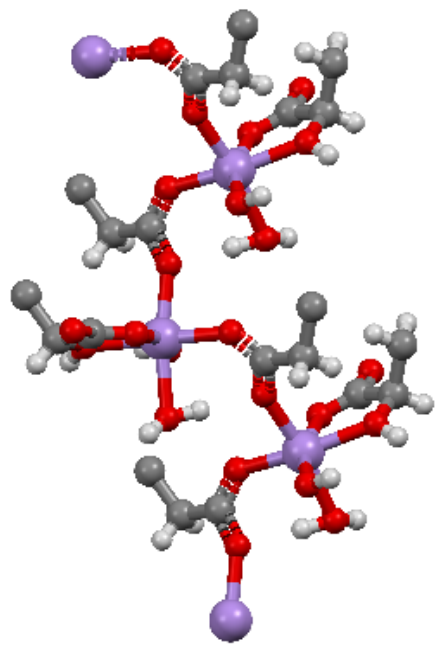 **1D RBB**	DL-mal:1	171.18	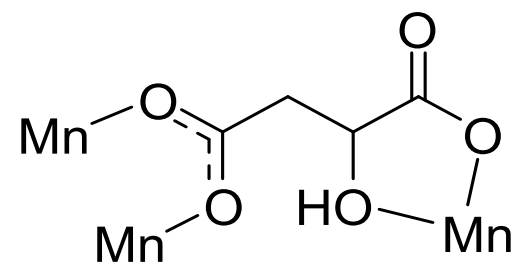 (κ^2)-(κ^1)-(κ^1)-μ_3	**2D Sheet**	N/A	
CEVYIM[10]	*P*2$_1$/*c*	[Zn(II)(DL-mal)(2,2'bipy)]$\cdot 2H_2O$	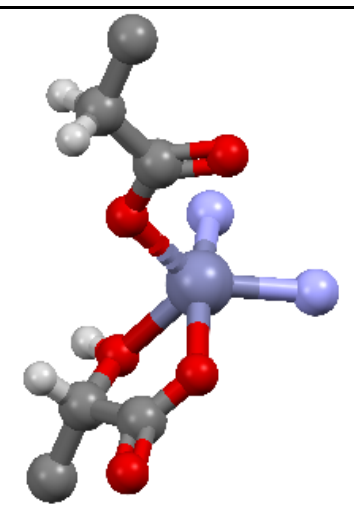 **Mononuclear**	DL-mal:1	59.338	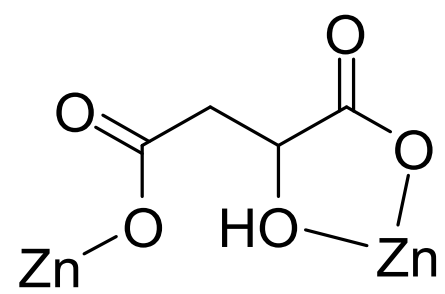 (κ^2)-(κ^1)-μ_2	**1D Polymer**	N/A	
CIMZII[11]	*P*2$_1$2$_1$2$_1$	[Zn(II)(L-mal)H_2O]	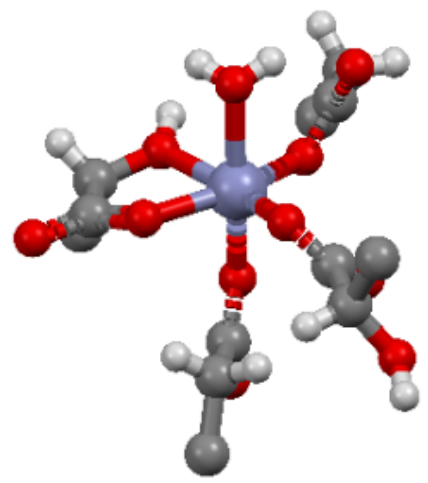 **Mononuclear**	L-mal:1	157.558	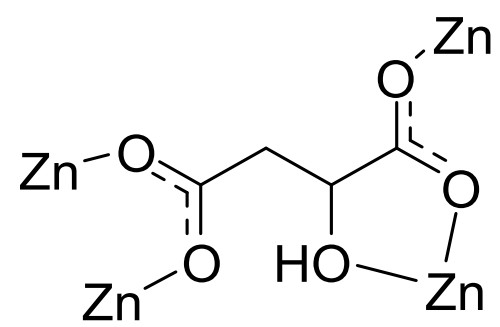 (κ^2)-(κ^1)-(κ^1)-(κ^1)-μ_4	**3D Network**	N/A	

CIQXIK[11]	$P2_1$	[Co(II)/Ni(II)(L-mal)$(H_2O)_2$]·H_2O	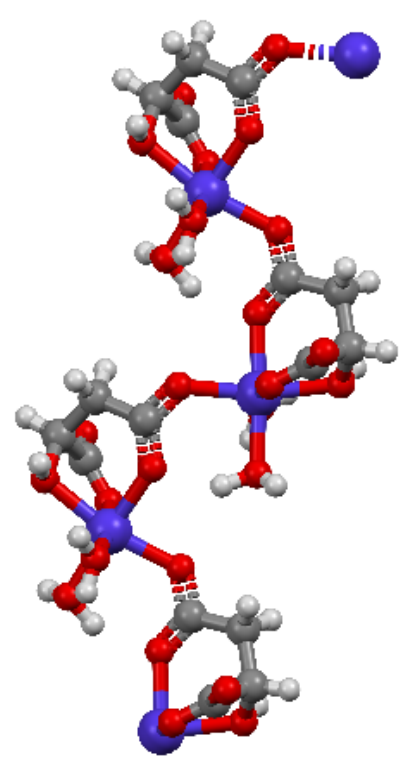 **1D RBB**	L-mal:1	64.841	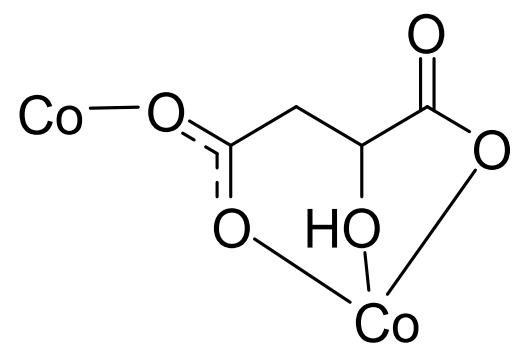 (κ3)-(κ1)-μ2	**1D Polymer**	N/A
COCLUC	$P2_12_12_1$	[Co(II)(L-mal)(MeOH)(H_2O)]	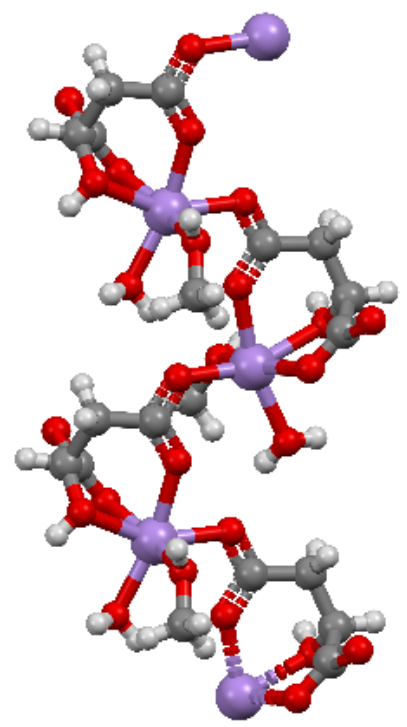 **1D RBB**	L-mal:1	59.531	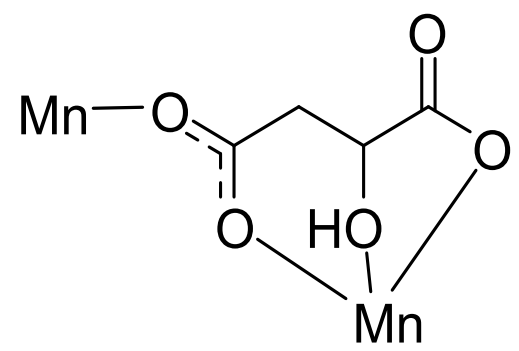 (κ3)-(κ1)-μ2	**1D Polymer**	N/A
				L-mal:2	64.361	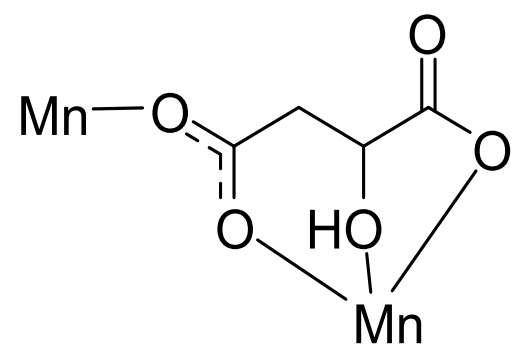 (κ3)-(κ1)-μ2		
COCWIE[12]	$P2_12_12$	[Ni(II)$_2$(D-mal)$_2$(4,4'bipy)]	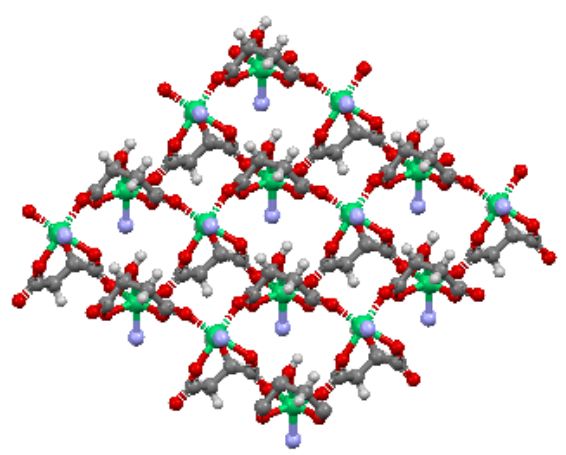 **2D Layer**	D-mal:1	67.098	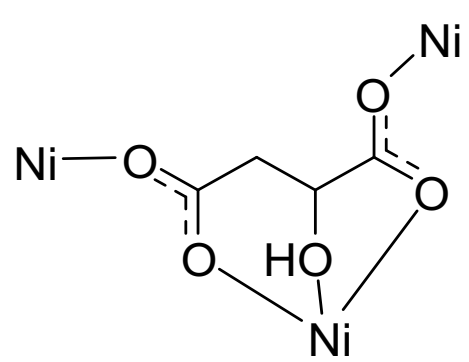 (κ1)-(κ3)-(κ1)-μ3	**2D Sheet**	4,4'-bipyridine 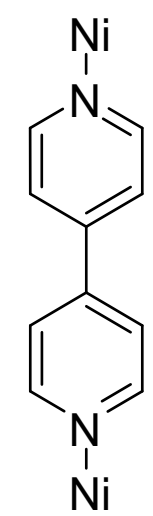 **3D Network**

CODFEH[13]	*P*4$_3$	[Mn(II)(L-mal)(H_2O)$_2$]·H_2O	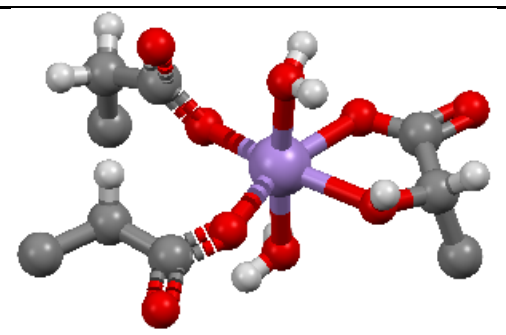 **Mononuclear**	L-mal:1	164.856	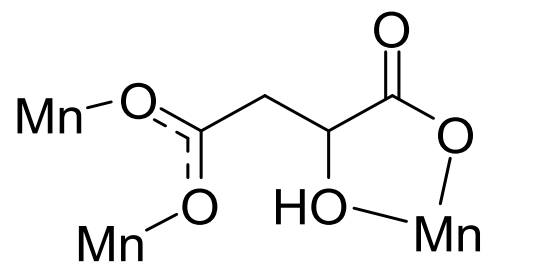 (κ^2)-(κ^1)-(κ^1)-μ$_3$	**3D Network**	N/A
COMALT[14]	*P*2$_1$	[Co(II)(L-mal)(H_2O)$_2$]·H_2O	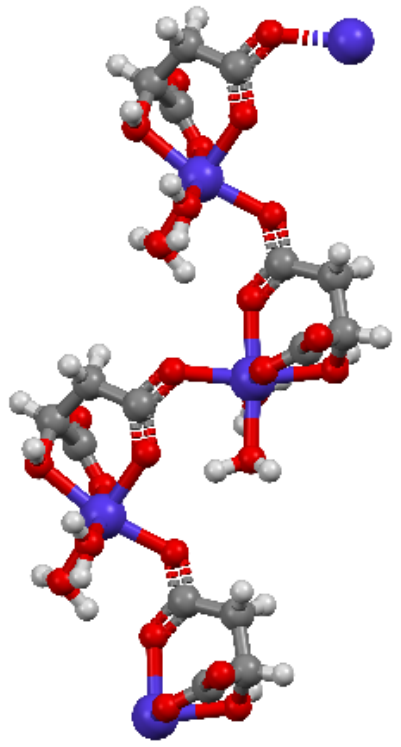 **1D RBB**	L-mal:1	64.854	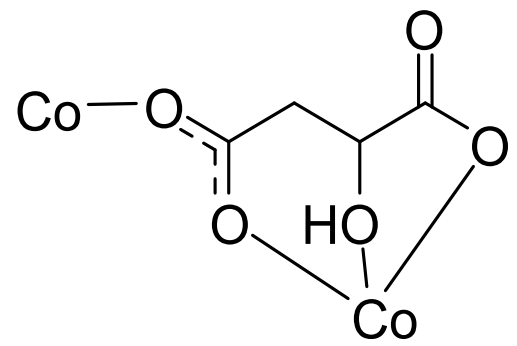 (κ^3)-(κ^1)-μ$_2$	**1D Polymer**	N/A
CUHMAM[15]	*P*112$_1$	[Cu(II)(L-Hmal)$_2$]·2H_2O	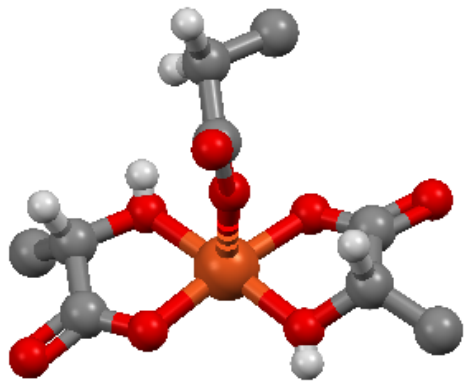 **Mononuclear**	L-Hmal:1	163.69	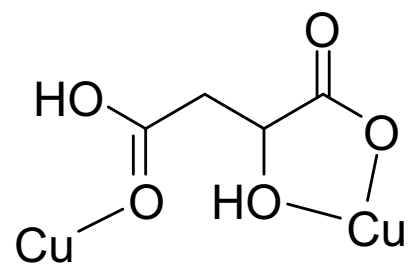 (κ^2)-(κ^1)-μ$_2$	**1D Polymer**	N/A
				L-Hmal:2	172.89	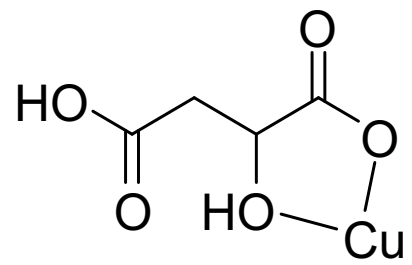 κ^2		

DONHEX [16]	$P2_12_12_1$	[Cd(II)(L-mal)(2,6-di(1H-imidazol-1-yl)pyridine)(H_2O)]	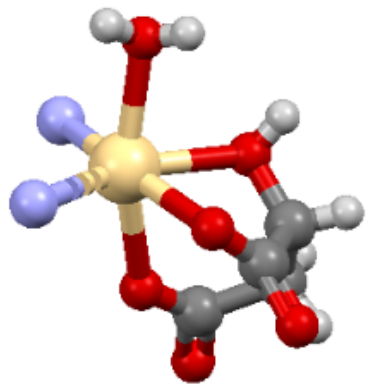 **Mononuclear**	L-mal:1	58.872	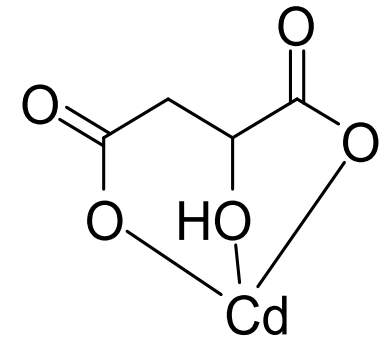 κ³	**0D Complex**	2,6-di(1H-imidazol-1-yl)pyridine) 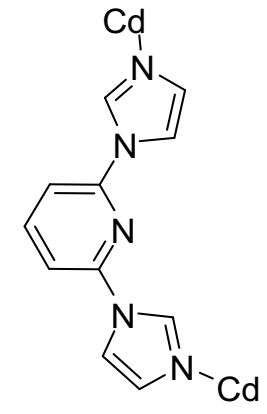 **1D Polymer**
DONHIB [16]	$P2_12_12_1$	[Cd(II)(L-mal)(2,6-di(1H-imidazol-1-yl)pyridine))(H_2O)]	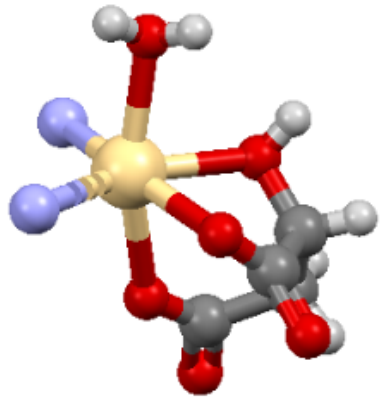 **Mononuclear**	L-mal:1	59.419	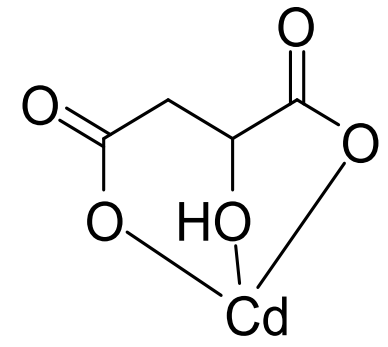 κ³	**0D Complex**	2,6-di(1H-imidazol-1-yl)pyridine) 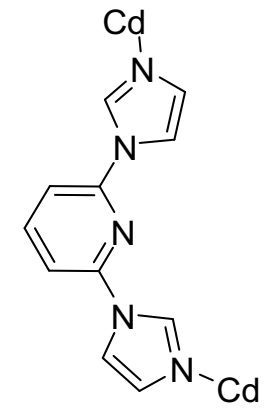 **1D Polymer**
DORFOG [17]	$P4_3$	[Mg(II)(L-mal)(H_2O)$_2$]·H_2O	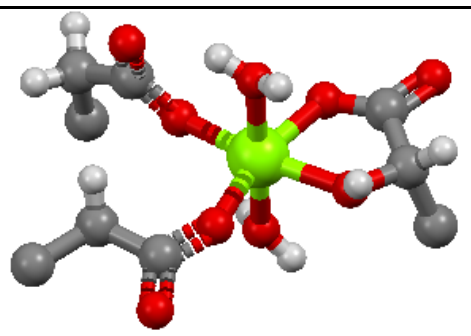 **Mononuclear**	L-mal:1	167.007	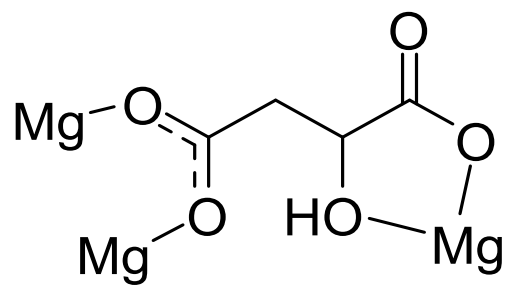 (κ²)-(κ¹)-(κ¹)-μ₃	**3D Network**	N/A
DUFSOM [18]	$P2_12_12$	[Cu(II)(L-mal)(N-isopropyl-2-methyl-1,2-propanediamine)]·5H_2O	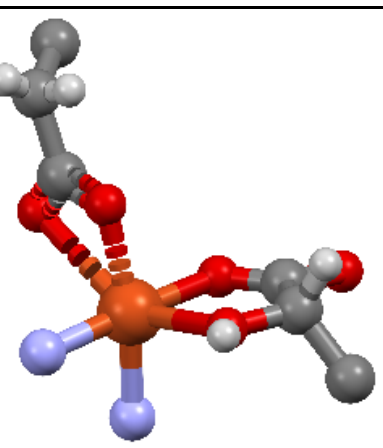 **Mononuclear**	L-mal:1	173.023	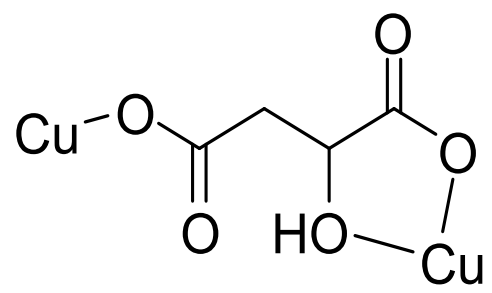 (κ²)-(κ¹)-μ₂	**1D Polymer**	N/A

EDAKIE[19]	*Cc*	[K(I)(DL-mal)(H_2O)]	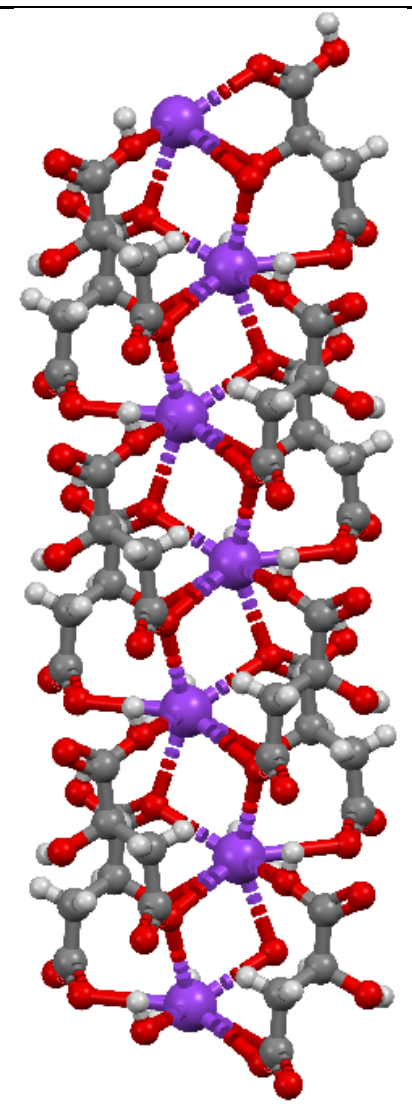 **1D RBB**	DL-mal:1	178.072	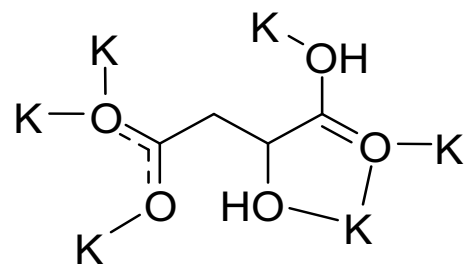 (κ^2)-(κ^1)-(κ^1)-(κ^1)-(κ^1)-(κ^1)-μ_6	**3D Network**	N/A
EGOKOB[20]	$P2_1$	[Mn(II)$_2$(L-mal)(succinate)(4,4'bipy)(H_2O)$_2$]·4,4'bipy	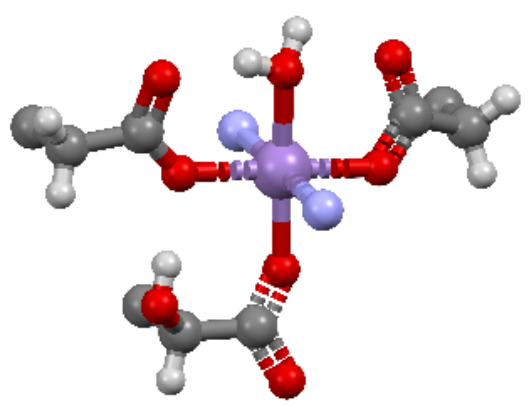 **Mononuclear**	L-mal:1	167.438	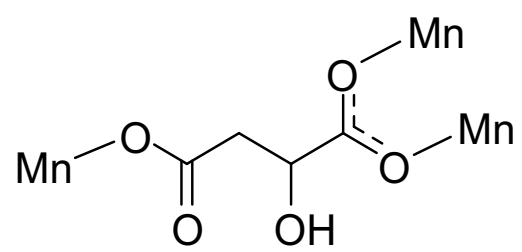 (κ^1)-(κ^1)-(κ^1)-μ_3	**1D Polymer**	4,4'-bipyridine 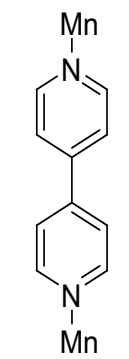 succinate 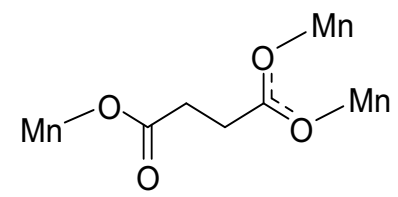 **3D Network**

EGOKUH[20]	*P*$2_12_12_1$	[Mn(II)(L-mal)(4,4'bipy)H_2O]· $5H_2O$	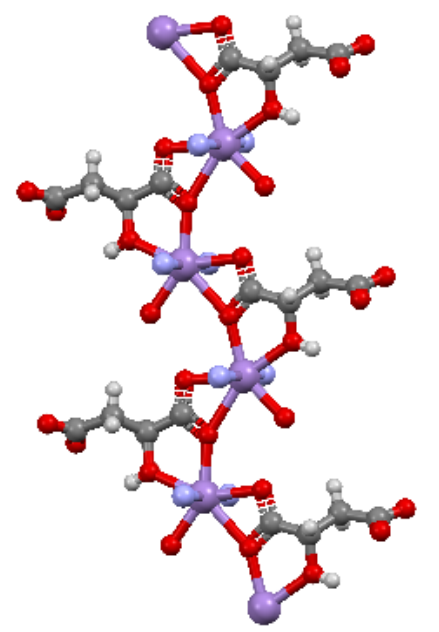<br>**1D RBB**	L-mal:1	170.689	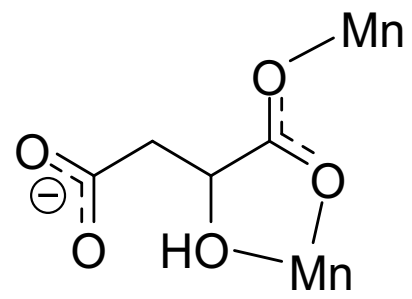<br>(κ²)-(κ¹)-μ₂	**1D Polymer**	4,4'-bipyridine<br>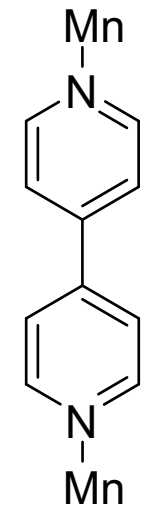<br>**2D Sheet**
EVODAW[21]	*P*2_1/*c*	[In(III)(D-mal)(L-mal)]·NH_4 H_2O	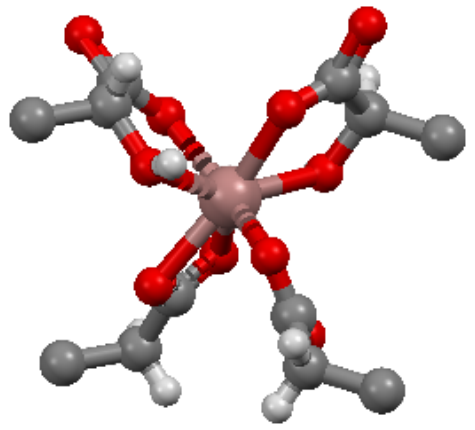<br>**Mononuclear**	L-mal:1	56.41	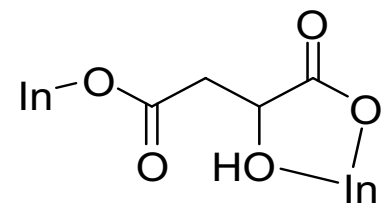<br>(κ²)-(κ¹)-μ₂	**1D Polymer**	N/A
				D-mal:2	56.41	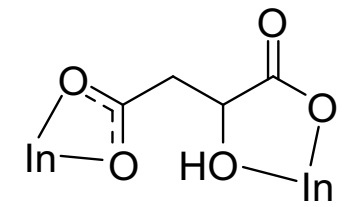<br>(κ²)-(κ²)-μ₂		
FADCIY[22]	*Pbca*	[Zn(II)(DL-mal)(Im)(H_2O)]· $2H_2O$	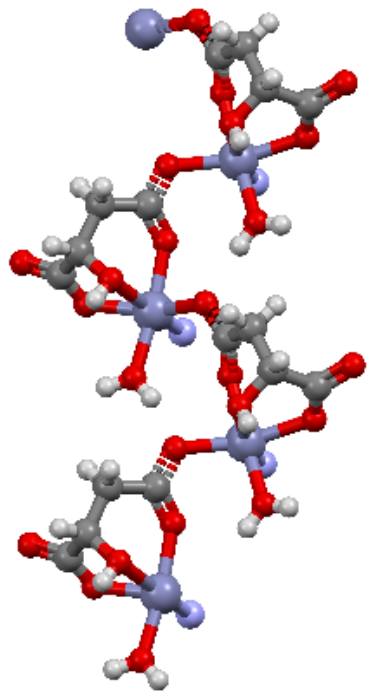<br>**1D RBB**	DL-mal:1	74.656	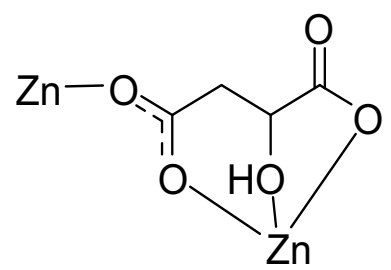<br>(κ³)-(κ¹)-μ₂	**1D Polymer**	N/A

FAYXEJ[23]	*Cc*	[Ni(II)(DL-mal)(H_2O)]·H_2O	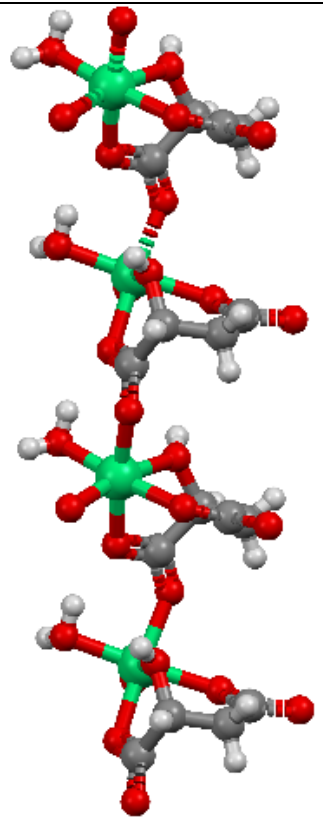 **1D RBB**	L-mal:1	73.022	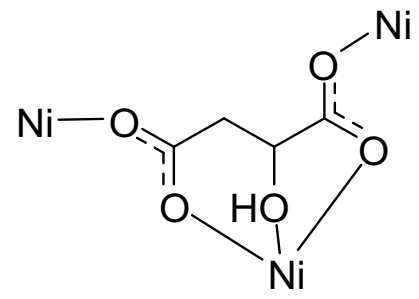 (κ^1)-(κ^3)-(κ^1)-μ_3	**3D Network**	N/A
FODZII[24]	*Pna*2$_1$	[Ni(II)(L-mal)(1,10 phenanthroline)]	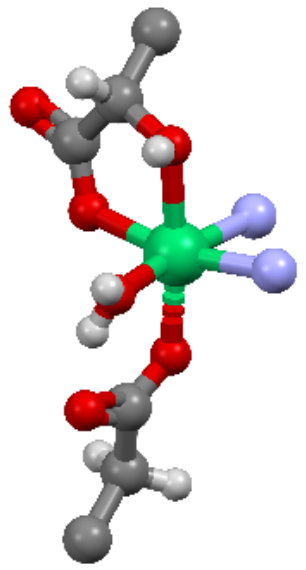 **Mononuclear**	L-mal:1	144.805	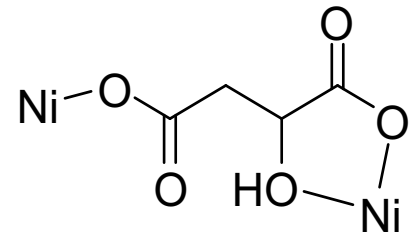 (κ^2)-(κ^1)-μ_2	**1D Polymer**	N/A
FOXDAY[25]	*P*2$_1$/*n*	[Co(II)(DL-mal)(H_2O)$_2$]·H_2O	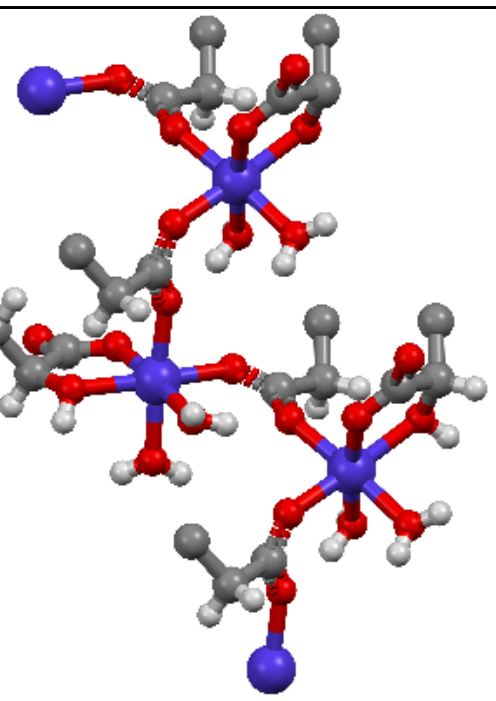 **1D RBB**	DL-mal:1	171.271	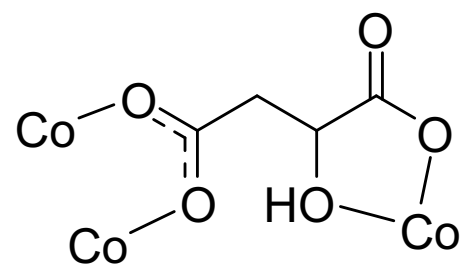 (κ^2)-(κ^1)-(κ^1)-μ_3	**2D Sheet**	N/A

FUHYUD[26]	*P*2$_1$/*n*	[Zn(II)(DL-mal)(H_2O)$_2$]·H_2O	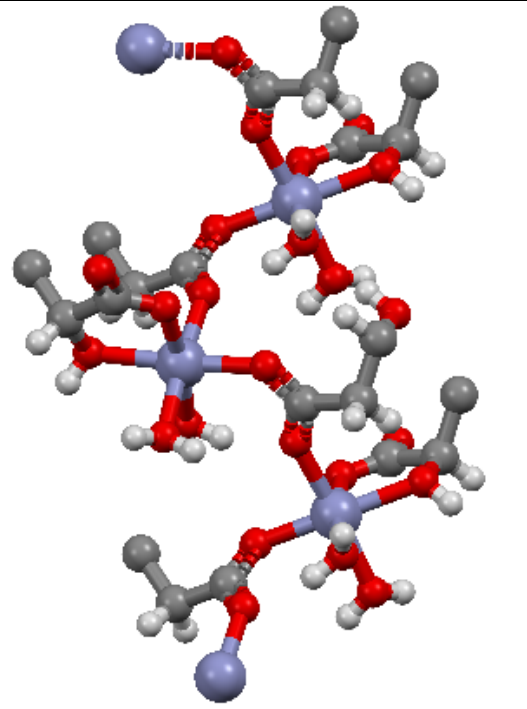<br>**1D RBB**	DL-mal:1	170.525	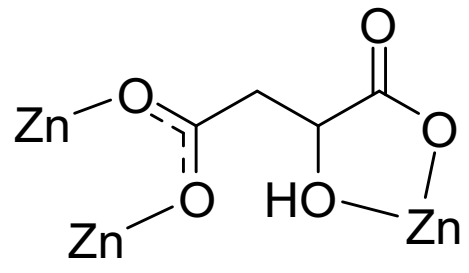<br>(κ^2)-(κ^1)-(κ^1)-μ_3	**2D Sheet**	N/A
GIXMOR[27]	P4$_3$2$_1$2	[Cu(II)(L-mal)(4,4'bipy)H_2O]· H_2O	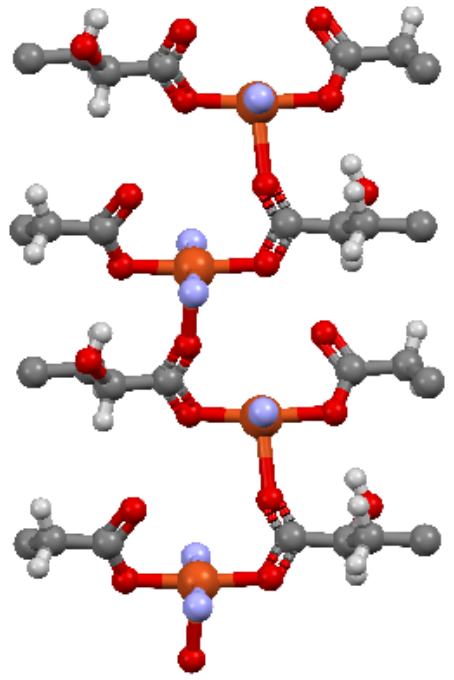<br>**1D RBB**	L-mal:1	179.251	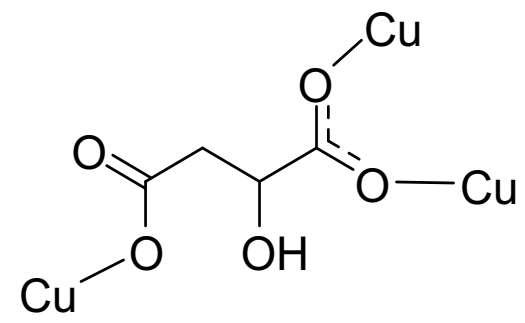<br>(κ^1) -(κ^1) -(κ^1)-μ_3	**2D Sheet**	4,4'-bipyridine<br>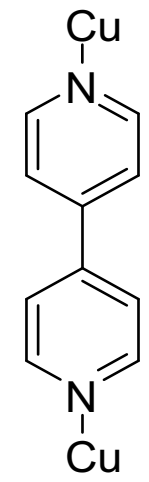<br>**3D Network**
GIXMUX[27]	*P*2$_1$2$_1$2$_1$	[Cu(II)(L-mal)(4,4'bpe)]·2H_2O	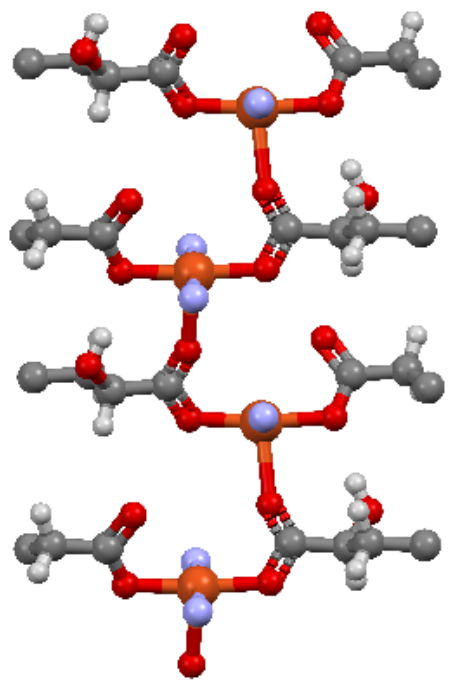<br>**1D RBB**	L-mal:1	179.216	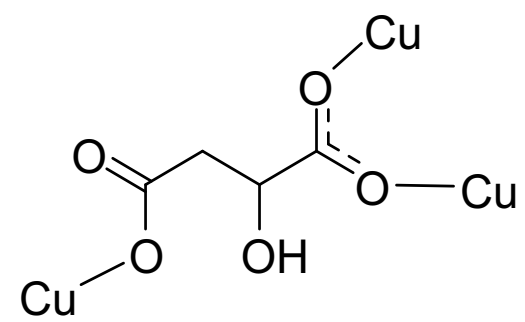<br>(κ^1) -(κ^1) -(κ^1)-μ_3	**2D Sheet**	trans-1,2-bis(4-pyridyl)ethylene<br>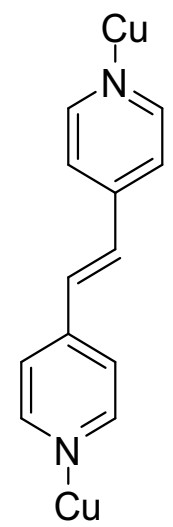<br>**3D Network**
				L-mal:2	178.691	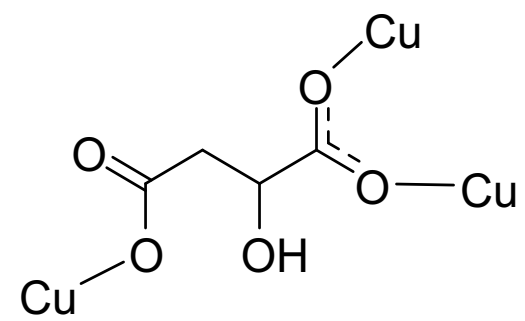<br>(κ^1) -(κ^1) -(κ^1)-μ_3		

GIXNAE[27]	$P2_12_12_1$	[Ni(II)(L-mal)(4,4'bipy)]·5MeOH	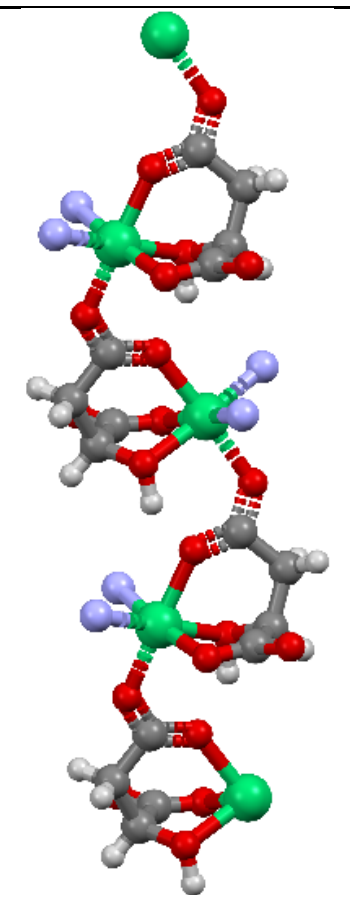 **1D RBB**	L-mal:1	61.121	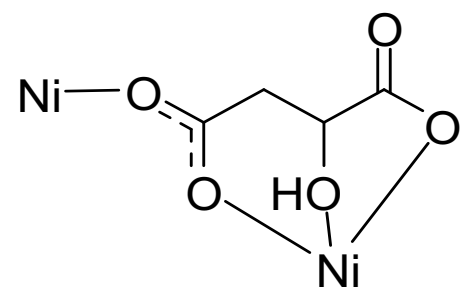 (κ^3)-(κ^1)-μ_2	**1D Polymer**	4,4'-bipyridine 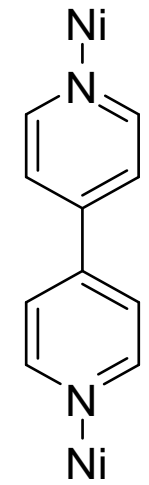 **3D Network**
GUTYIH[28]	$P2_1/n$	[Ag(I)$_2$(DL-mal)(3-methylpyridine)$_4$]	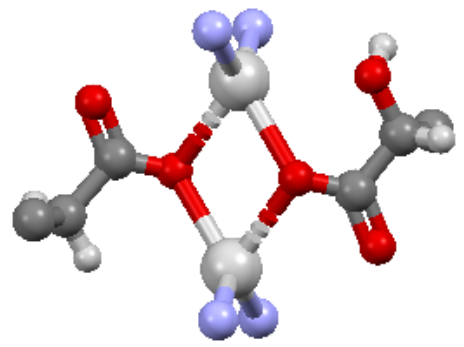 **Dinuclear**	DL-mal:1	179.669	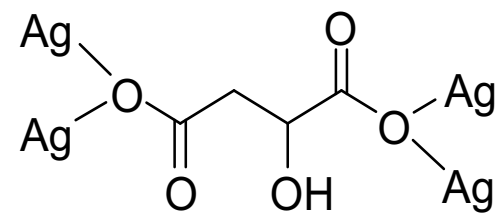 (κ^1)-(κ^1)-(κ^1)-(κ^1)-μ_4	**1D Polymer**	N/A

HESXOT[29]	*P*$2_12_12_1$	[K(I)$_2$(L-H$_2$mal)(L-Hmal)$_2$]	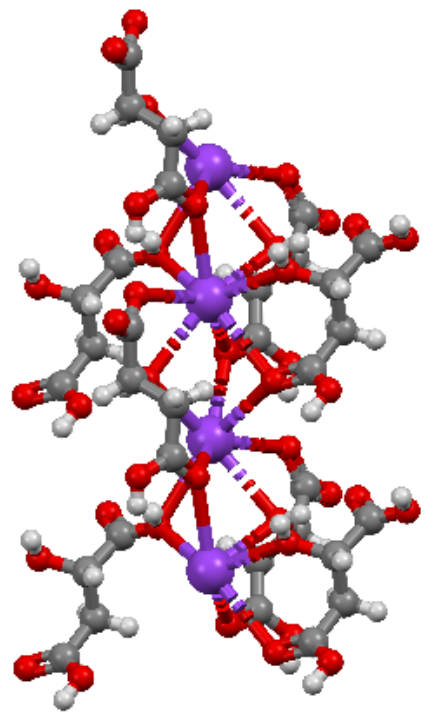<br>**1D RBB**	L-H$_2$mal:1	176.605	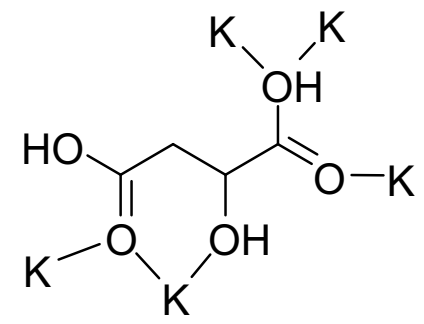<br>(κ^1)-(κ^1)-(κ^1)-(κ^2)-(κ^1)-μ_5	**2D Sheet**	N/A
				L-Hmal:2	167.938	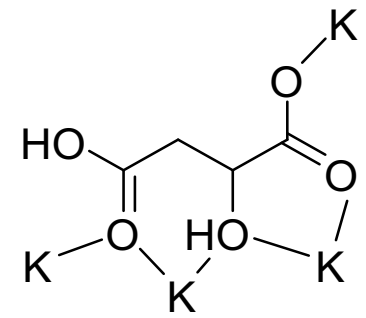<br>(κ^1)-(κ^2)-(κ^2)-(κ^1)-μ_4		
				L-Hmal:3	162.518	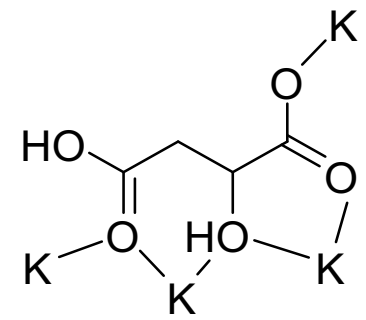<br>(κ^1)-(κ^2)-(κ^2)-(κ^1)-μ_4		
HILGAO[30]	*Pbca*	[Mg(II)(DL-mal)(H$_2$O)]·2H$_2$O	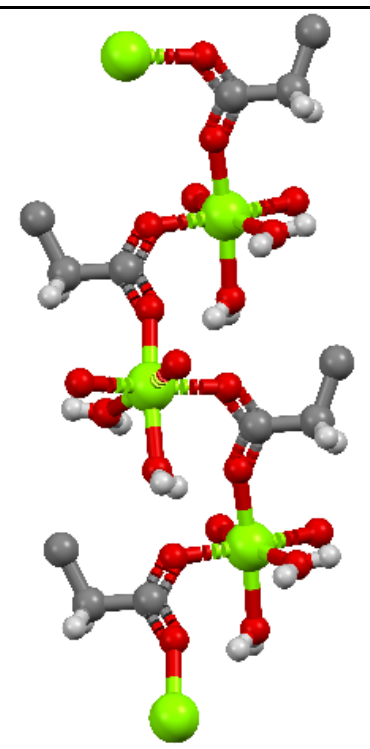<br>**1D RBB**	DL-mal:1	174.895	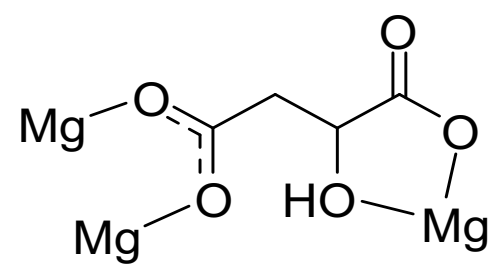<br>(κ^2)-(κ^1)-(κ^1)-μ_3	**2D Sheet**	N/A

HOLWEL[31]	*P*-1	[Ca(II)(DL-mal)(H_2O)$_2$]·H_2O	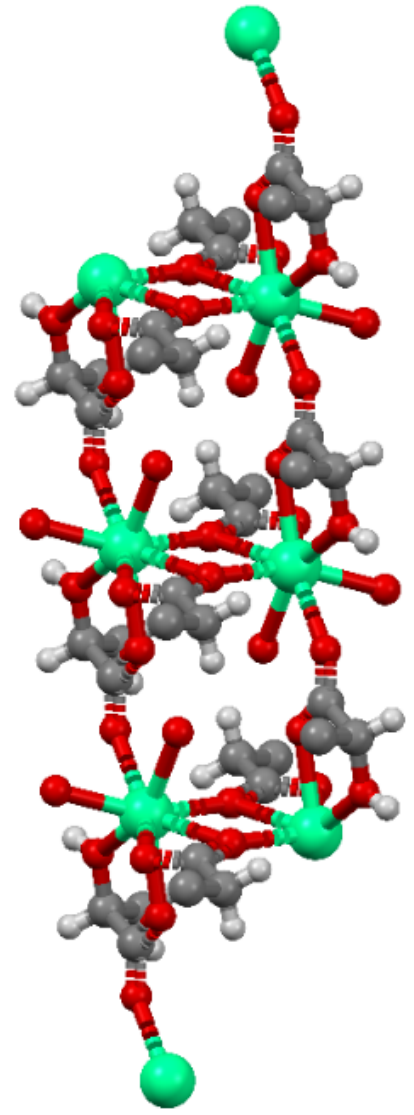 **1D RBB**	DL-mal:1	174.191	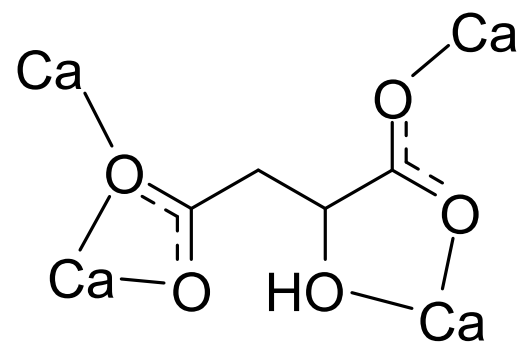 (κ^2)-(κ^1)-(κ^2)-(κ^1)-μ$_4$	**2D Sheet**	N/A
IDATUE[32]	*Fdd*2	[Zn(II)(D-mal)(4,4'bipy)]·2H_2O	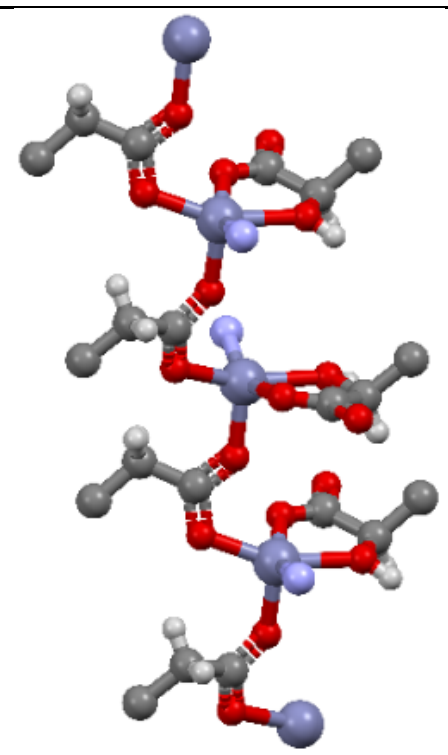 **1D RBB**	D-mal:1	159.518	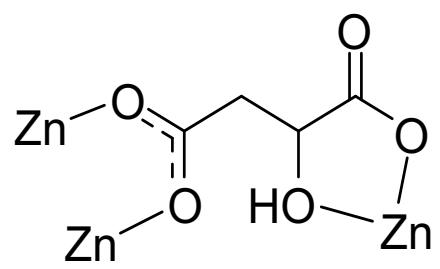 (κ^2)-(κ^1)-(κ^1)-μ$_3$	**2D Sheet**	N/A

IWETAF[33]	$P2_1$	$[Ca(II)(L\text{-}mal)(H_2O)_3]$	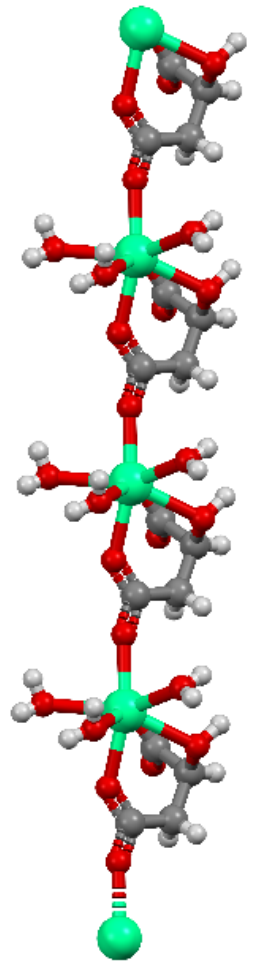 **1D RBB**	L-mal:1	66.428	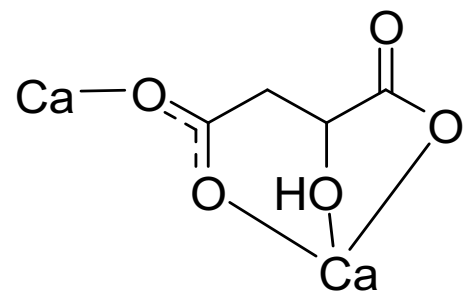 (κ^3)-(κ^1)-μ_2	**1D Polymer**	N/A
KOGHEU[34]	$P2_12_12_1$	$[Cd(II)(L\text{-}mal)]$	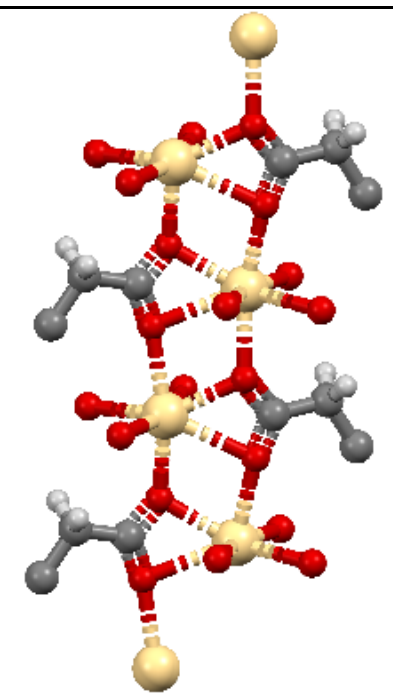 **1D RBB**	L-mal:1	158.839	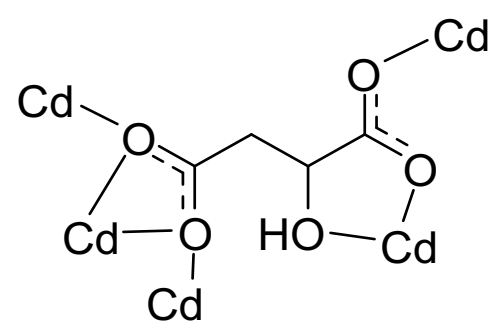 (κ^1)-(κ^2)-(κ^1)-(κ^2)-(κ^1)-μ_5	**3D Network**	N/A

KUVGAN[35]	$P2_12_12_1$	$[Mg(II)(L\text{-}mal)(H_2O)]$	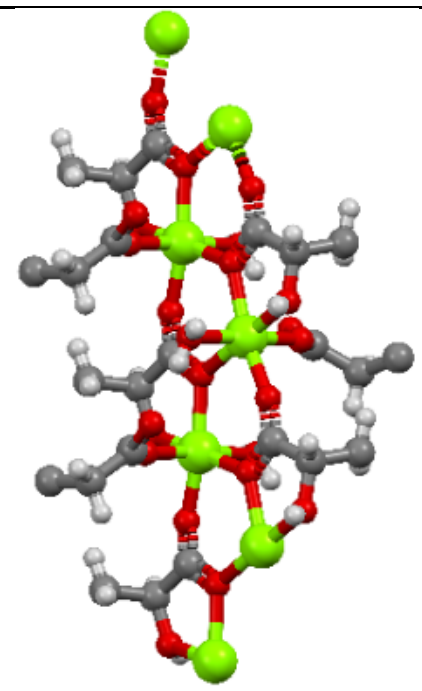 1D RBB	L-mal:1	174.117	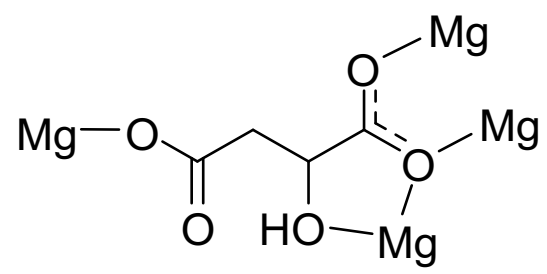 (κ^1)-(κ^1)-(κ^2)-(κ^1)-μ_4	**3D Network**	N/A
KUWWEI[36]	$P2_12_12$	$[Ni(II)_2(D\text{-}mal)_2)(4,4'bipy)]\cdot Xe$	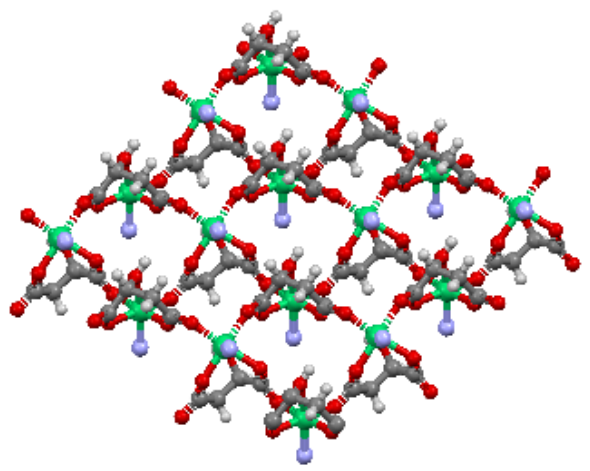 2D Layer	D-mal:1	68.049	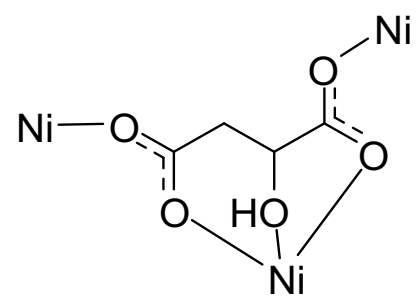 (κ^1)-(κ^3)-(κ^1)-μ_3	**2D Sheet**	4,4'-bipyridine 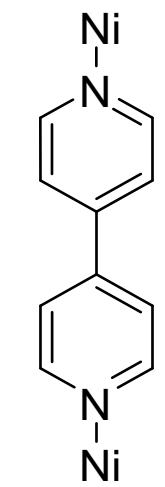 **3D Network**
KUWWIM[36]	$P2_1/c$	$[Ni(II)_2(DL\text{-}mal)_2)(4,4'bipy)]\cdot 2Xe$	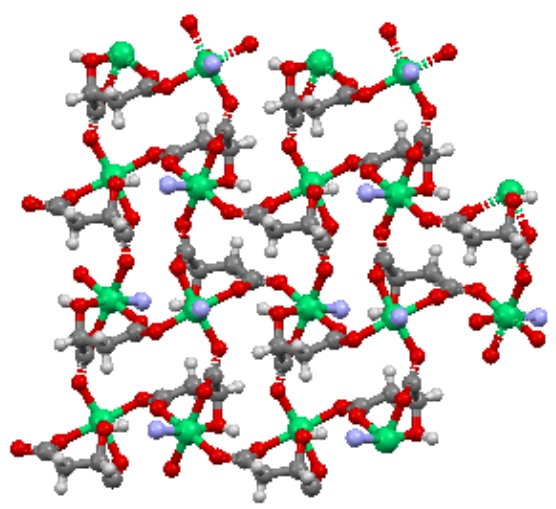 2D Layer	DL-mal:1	69.817	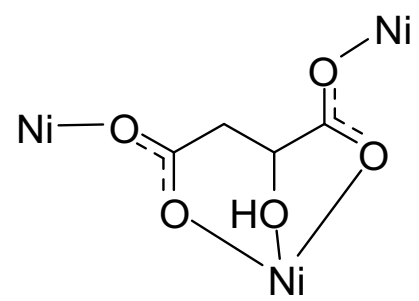 (κ^1)-(κ^3)-(κ^1)-μ_3	**2D Sheet**	4,4'-bipyridine 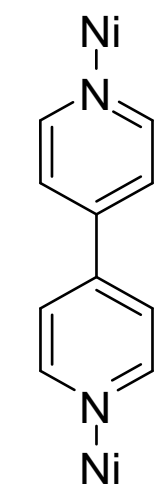 **3D Network**

LEHCIL[37]	*P*-1	[Eu(III)$_2$(D-mal)(L-mal)(fume)(1, 10 phen)$_2$]·H_2O	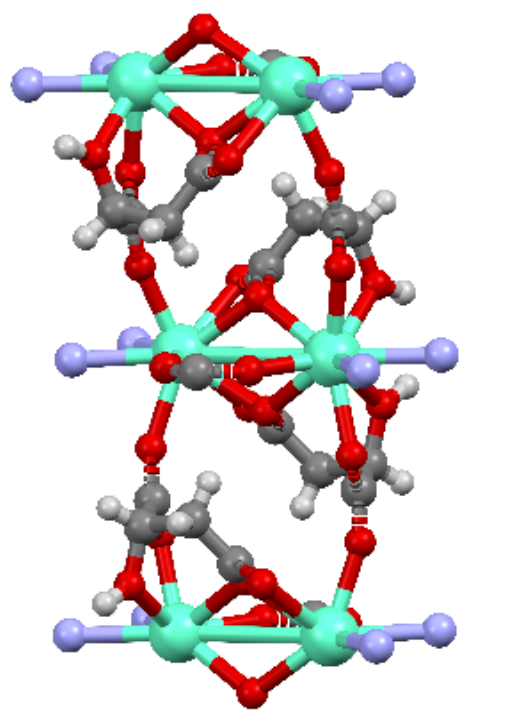<br>**1D RBB**	D-mal:1	81.11	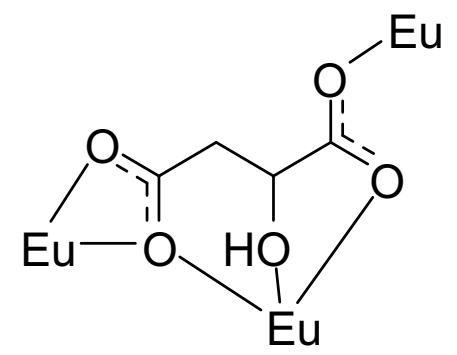<br>(κ1)-(κ3)-(κ2)-μ3	**1D Polymer**	Fumarate<br>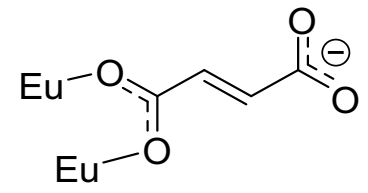<br>**1D Polymer**
				L-mal:1	79.657	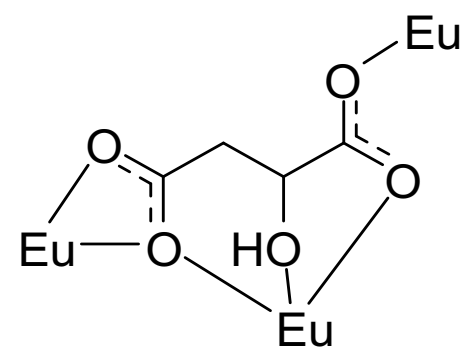<br>(κ1)-(κ3)-(κ2)-μ3		
LEHCOR[37]	*P*-1	[Pr(III)$_2$(D-mal)(L-mal)(fume)(1, 10 phen)$_2$]·H_2O	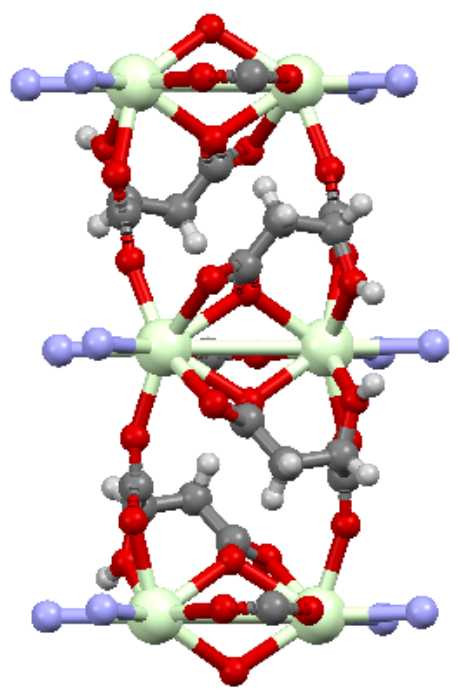<br>**1D RBB**	D-mal:1	81.665	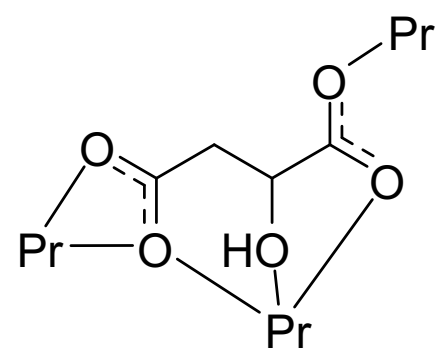<br>(κ1)-(κ3)-(κ2)-μ3	**1D Polymer**	Fumarate<br>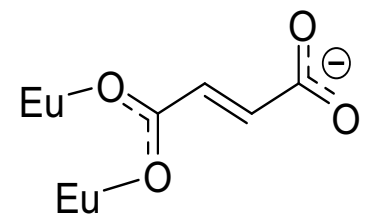<br>**1D Polymer**
				L-mal:1	78.619	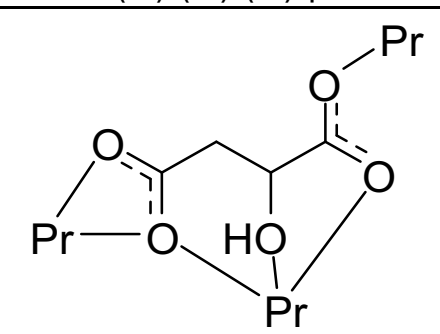<br>(κ1)-(κ3)-(κ2)-μ3		

LIKBAJ[38]	$Pccn$	[Cu(II)(DL-mal)(bispyridylpropane)]·6H_2O	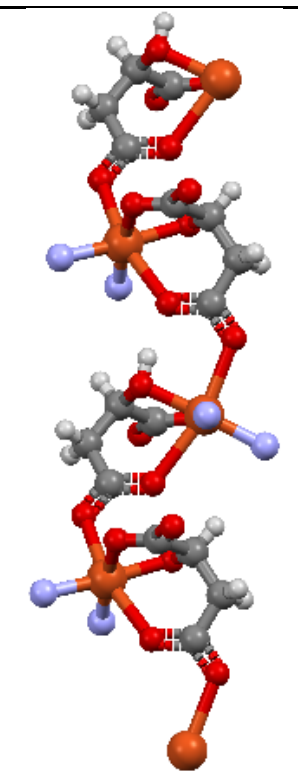 **1D RBB**	DL-mal:1	61.134	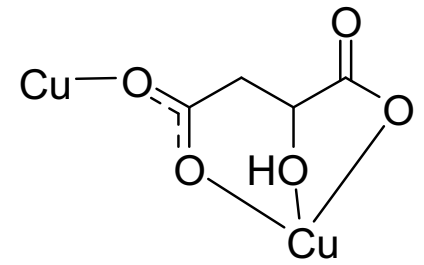 (κ³)-(κ¹)-μ₂	**1D Polymer**	bis(1,3-bis(4-pyridyl)propane 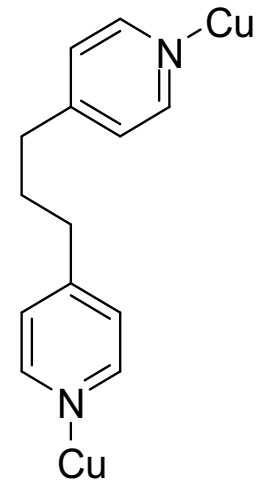 **3D Network**
MALAMN[39]	$P2_12_12_1$	[Mn(II)(L-mal)(H_2O)$_2$]· H_2O	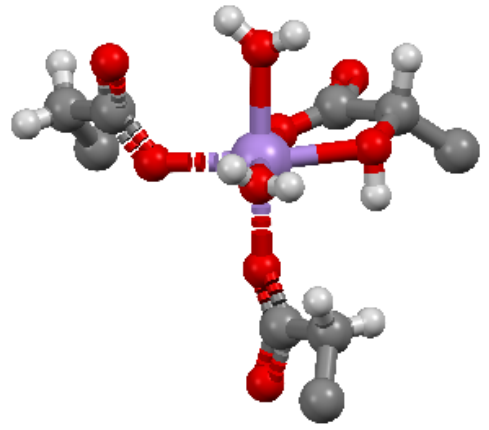 **Mononuclear**	L-mal:1	172.176	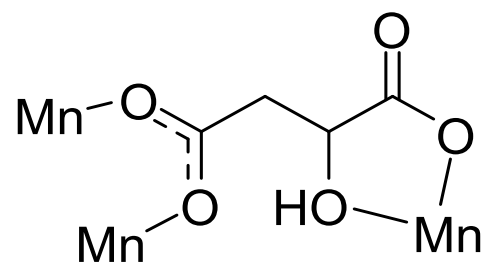 (κ²)-(κ¹)-(κ¹)-μ₃	**3D Network**	N/A
MALAZN[40]	$P2_1$	[Zn(II)(L-mal)(H_2O)$_2$]· H_2O	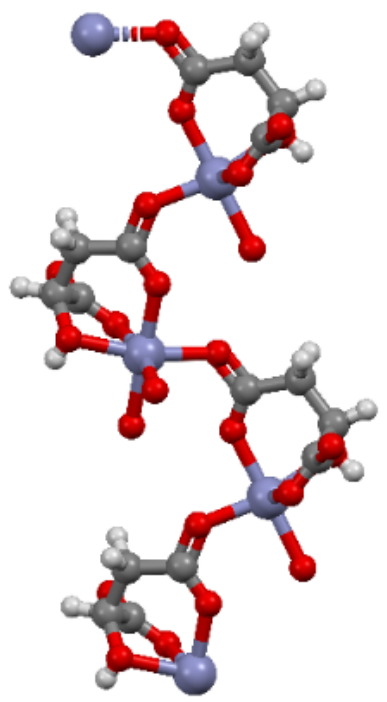 **1D RBB**	L-mal:1	65.259	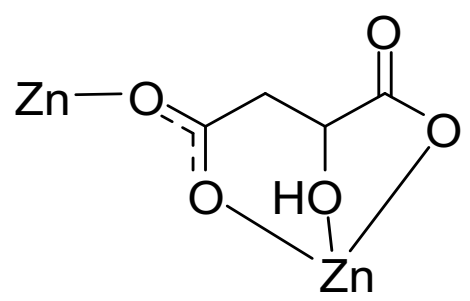 (κ³)-(κ¹)-μ₂	**1D Polymer**	N/A

MEFMEQ [41]	$P2_1/n$	[Cu(II)(DL-mal)(bis(4-pyridyl)disulfide)]·$6H_2O$	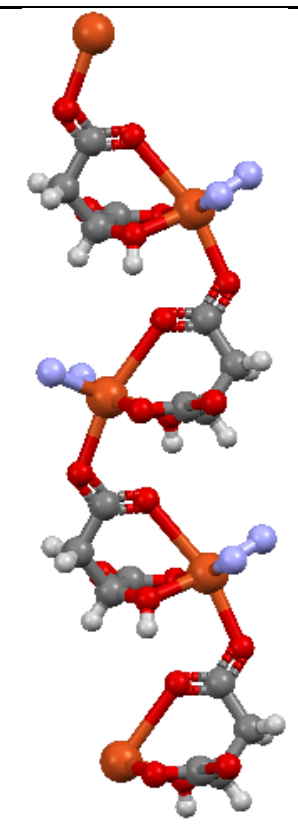<br>**1D RBB**	DL-mal:1	64.283	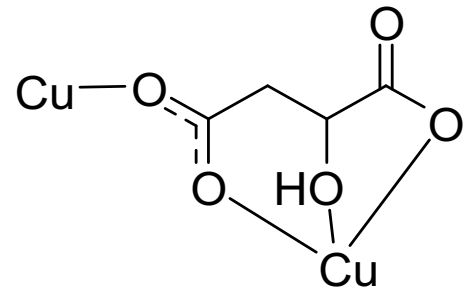<br>(κ^3)-(κ^1)-μ$_2$	**1D Polymer**	bis(4-pyridyl)disulfide<br>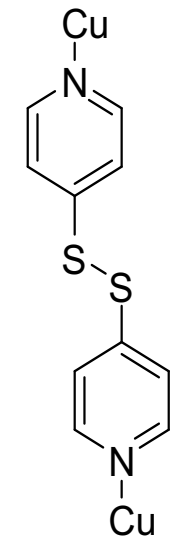<br>**2D Sheet**
MEYDUP [42]	$P2_1/c$	[Li(I)$_2$(DL-mal)(H_2O)$_2$]	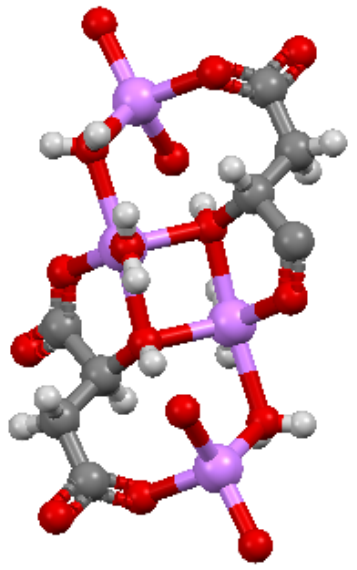<br>**Tetranuclear**	DL-mal:1	156.406	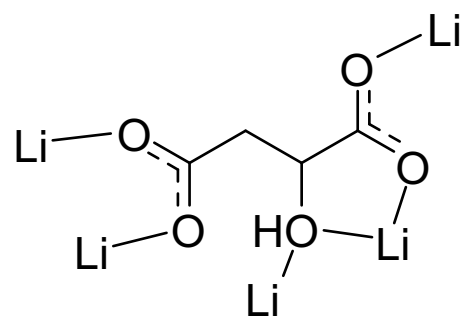<br>(κ^1)-(κ^2)-(κ^1)-(κ^1)-(κ^1)-μ$_5$	**3D Network**	Aqua<br>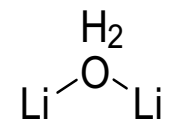<br>**3D Network**
MEYFEB [42]	$P2_1/n$	[Cd(II)(DL-mal)(H_2O)$_2$]·H_2O	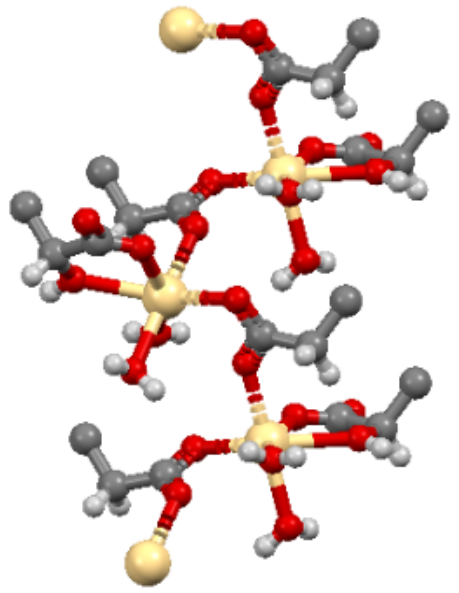<br>**1D RBB**	DL-mal:1	163.412	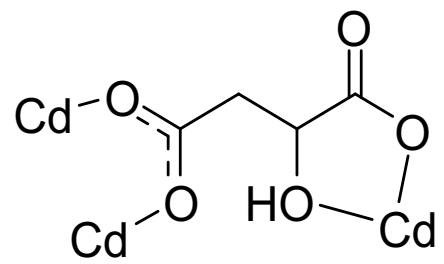<br>(κ^2)-(κ^1)-(κ^1)-μ$_3$	**2D Sheet**	N/A

MEYFIF[42]	*P*-1	[Ba(II)$_2$(D-mal)(L-mal)(H$_2$O)$_5$]	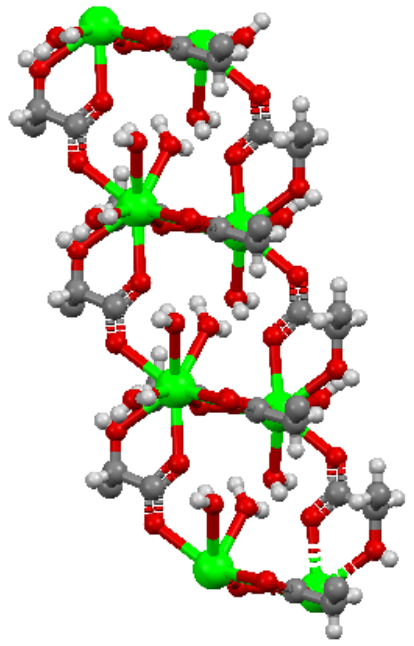 **1D RBB**	D-mal:1	176.088	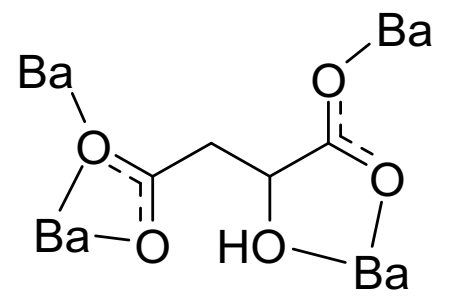 (κ^2)-(κ^1)-(κ^2)-(κ^1)-μ_4	**2D Sheet**	N/A
				L-mal:2	174.225	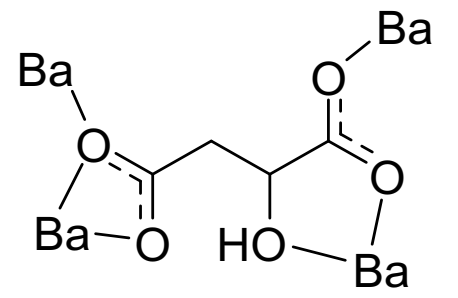 (κ^2)-(κ^1)-(κ^2)-(κ^1)-μ_4		
MEYFOL[42]	*P*-1	[Ba(II)$_2$(D-mal)(L-mal)(H$_2$O)$_2$]	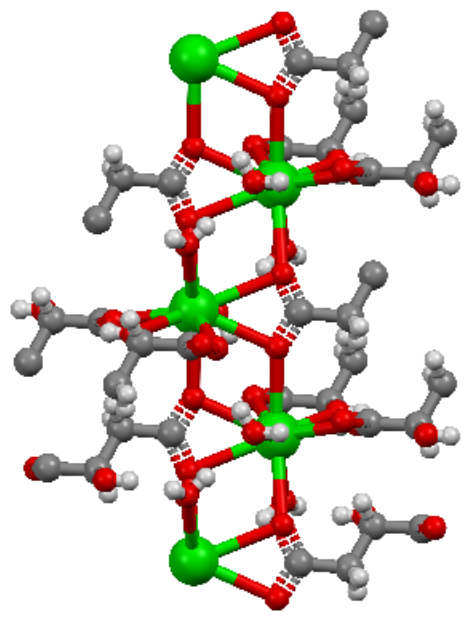 **1D RBB**	DL-mal:1	167.79	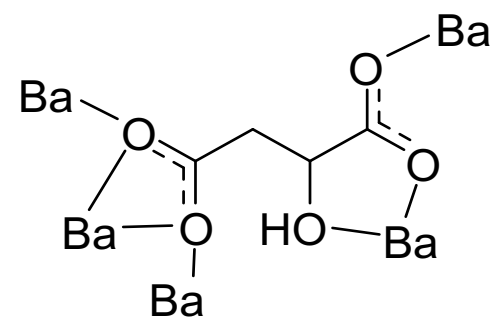 (κ^1)-(κ^2)-(κ^1)-(κ^2)-(κ^1)-μ_5	**3D Network**	N/A
MEYFUR[42]	*Fdd*2	[Rb(I)(DL-Hmal)]	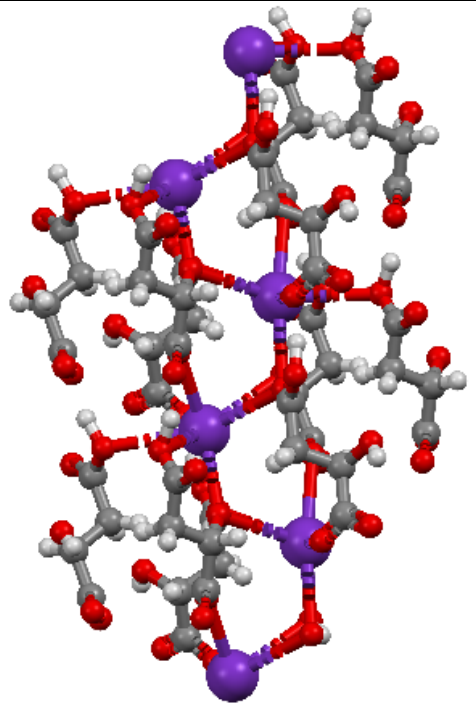 **1D RBB**	DL-mal:1	178.393	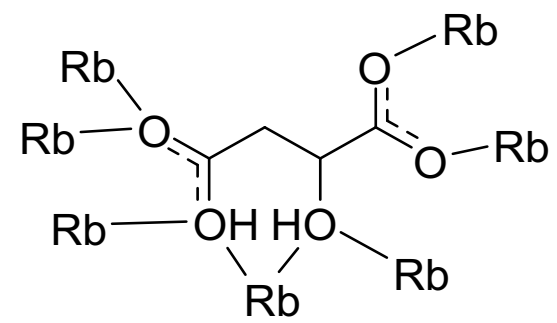 (κ^1)-(κ^1)-(κ^1)-(κ^2)-(κ^1)-(κ^1)-(κ^1)-μ_7	**3D Network**	N/A

MEYGAY[42]	*P*2$_1$/*n*	[Cs(I)(DL-Hmal)(H_2O)]	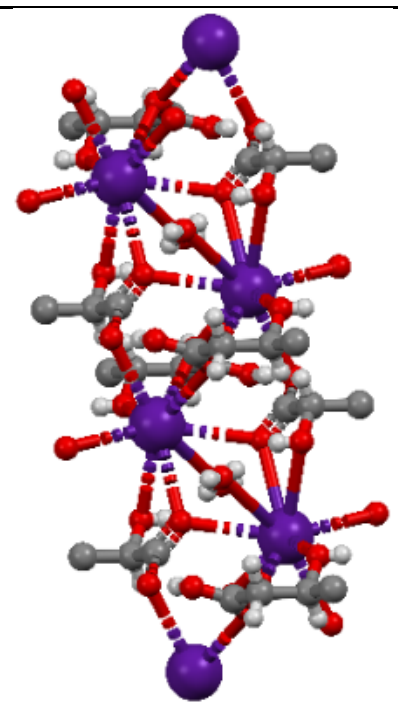 **1D RBB**	DL-mal:1	175.9	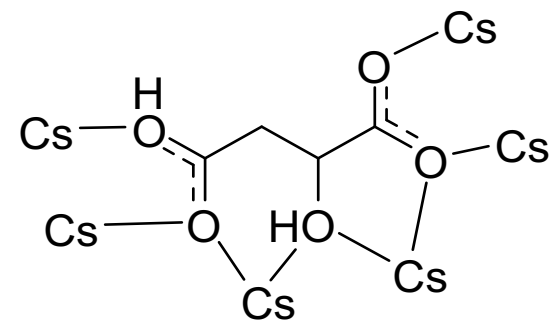 (κ^1)-(κ^1)-(κ^2)-(κ^2)-(κ^1)-(κ^1)-μ_6	**3D Network**	N/A
MEYHAZ[42]	*P*-1	[Cs(I)(DL-Hmal)(DL-H_2mal)]	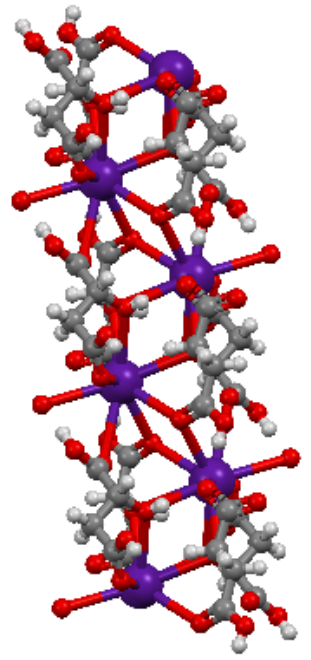 **1D RBB**	DL-H_2mal:1	177.443	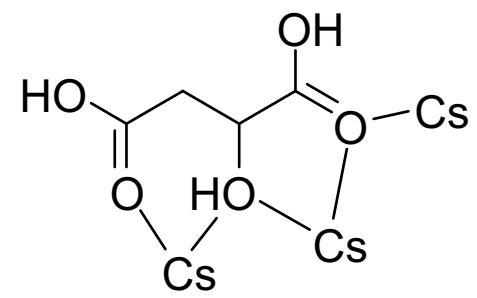 (κ^1)-(κ^2)-(κ^2)-μ_3	**2D Sheet**	N/A
				DL-Hmal:2	175.513	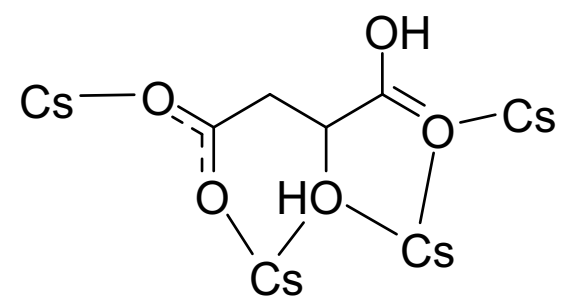 (κ^1)-(κ^2)-(κ^2)-(κ^1)-μ_4		
MIWNAJ[43]	*P*-1	[Pr(III)$_4$(DL-mal)$_4$(OH)$_2$(fumerate)]	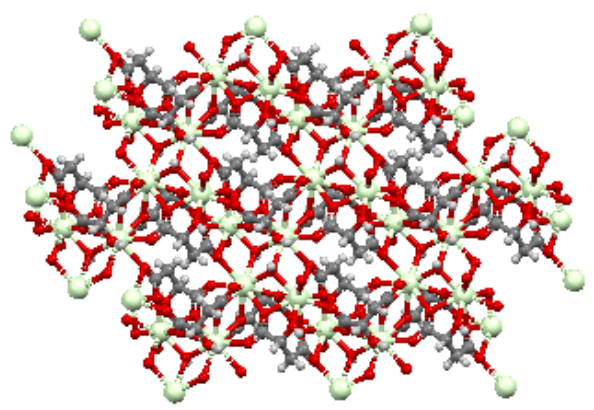 **2D Layer**	DL-mal:1	62.985	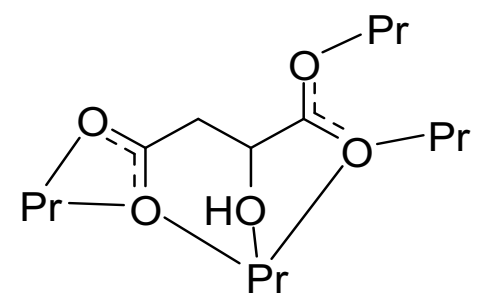 (κ^1)-(κ^1)-(κ^3)-(κ^2)-μ_4	**2D Sheet**	Fumerate 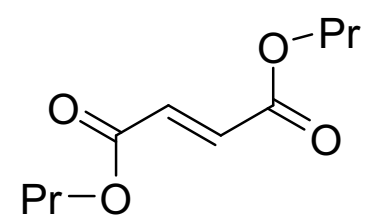 Hydroxide 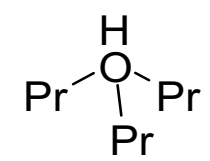 **3D Network**
				DL-mal:2	67.307	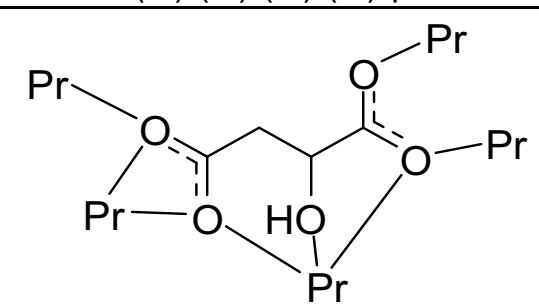 (κ^1)-(κ^1)-(κ^3)-(κ^2)-(κ^1)-μ_5		

MIWNEN [43]	*P*-1	$[Nd(III)_4(DL\text{-}mal)_4(OH)_2(fumerate)]$	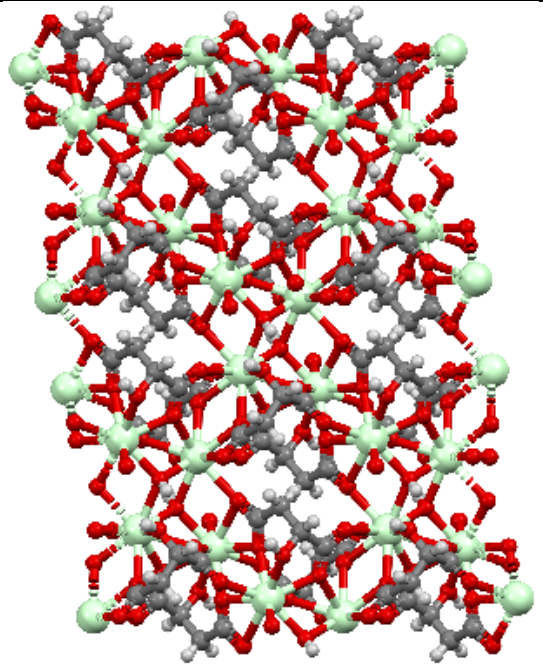<br>**2D Layer**	DL-mal:1	64.217	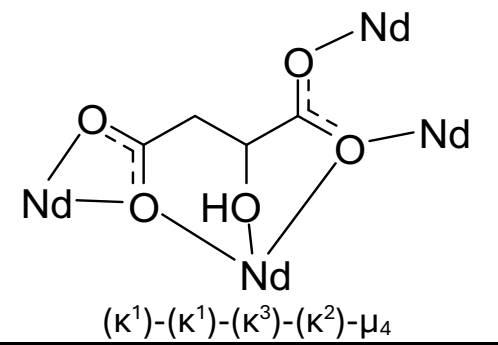<br>(κ¹)-(κ¹)-(κ³)-(κ²)-μ₄	**2D Sheet**	Fumerate<br>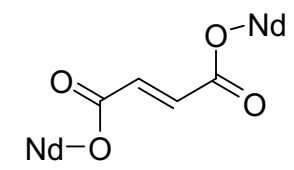<br>Hydroxide<br>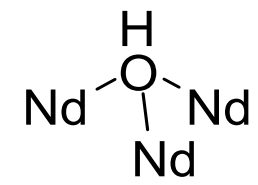<br>**3D Network**
				DL-mal:2	66.666	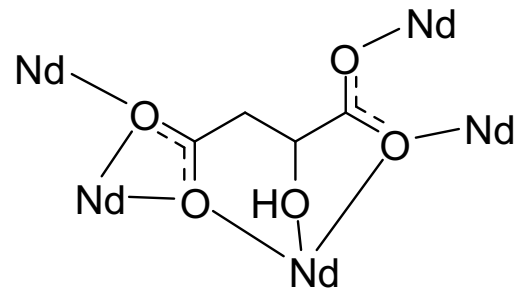<br>(κ¹)-(κ¹)-(κ³)-(κ²)-(κ¹)-μ₅		
OFUYUK[44]	*Fdd*2	$[Cu(II)(DL\text{-}mal)(1,2\text{-}bis(4\text{-}pyridyl)ethane)]\cdot 5H_2O$	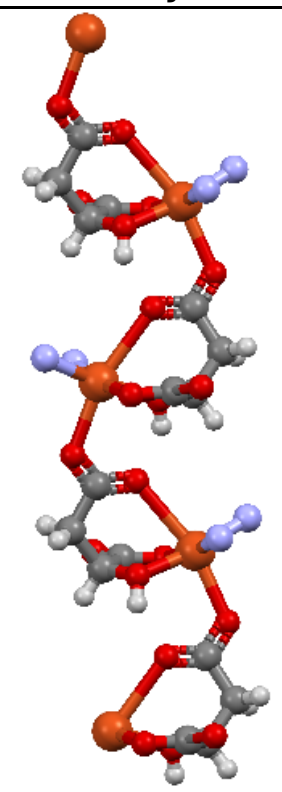<br>**1D RBB**	DL-mal:1	63.91	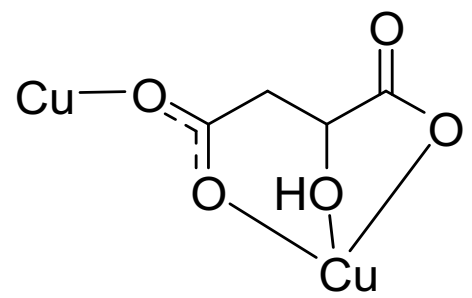<br>(κ³)-(κ¹)-μ₂	**1D Polymer**	1,2-bis(4-pyridyl)ethane<br>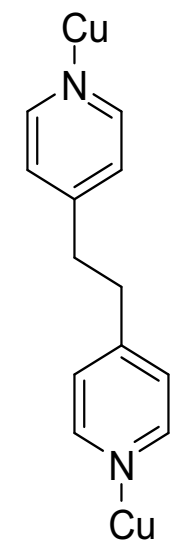<br>**2D Sheet**
PACLIP[45]	*Pna*2₁	$[Zn(II)(DL\text{-}mal)(1,10\ phen)(H_2O)]$	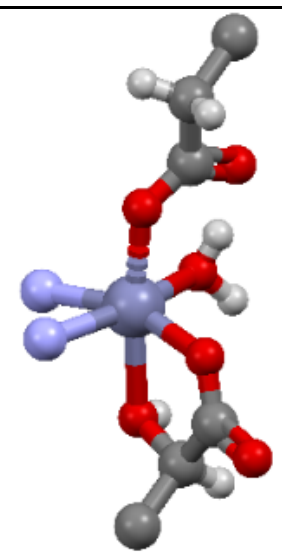<br>**Mononuclear**	DL-mal:1	147.368	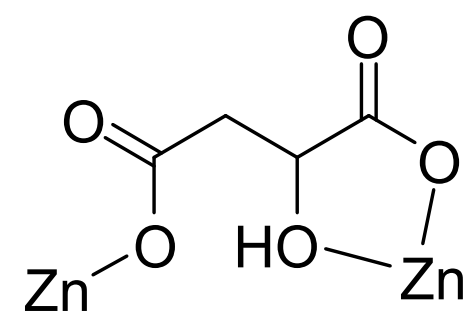<br>(κ²)-(κ¹)-μ₂	**1D Polymer**	N/A

PACLOV[45]	*$P2_1/c$*	[Cu(II)(DL-mal)(2,2'bipy)]·3H_2O	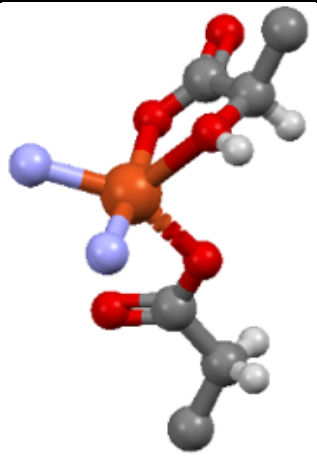 **Mononuclear**	DL-mal:1	61.317	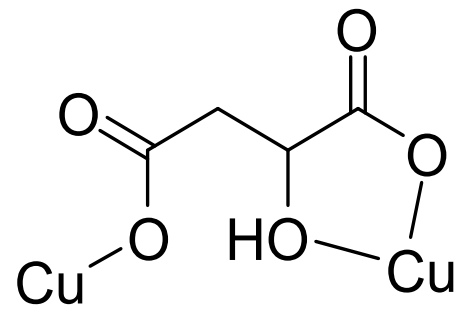 (κ²)-(κ¹)-μ₂	**1D Polymer**	N/A
PIDTOO[46]	*$P2_12_12_1$*	[Cu(II)(L-mal)(2,2'bipy)]·3H_2O	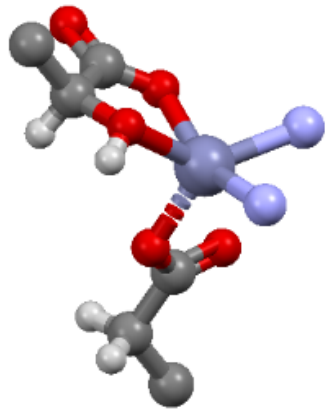 **Mononuclear**	L-mal:1	61.8	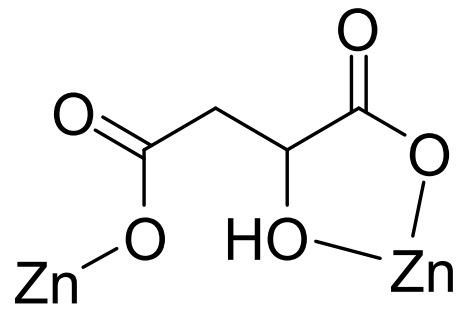 (κ²)-(κ¹)-μ₂	**1D Polymer**	N/A
PIDTUU[46]	*$P2_12_12_1$*	[Zn(II)$_2$(L-mal)$_2$(1,10 phen)$_2$(H_2O)$_2$]	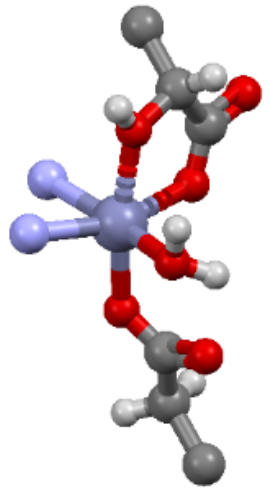 **Mononuclear**	L-mal:1	155.499	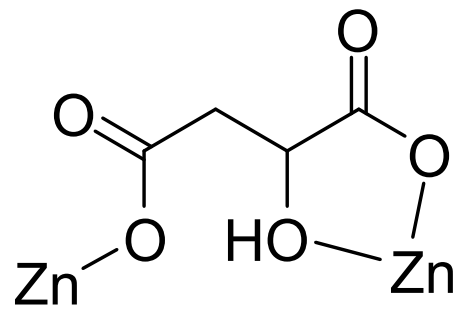 (κ²)-(κ¹)-μ₂	**1D Polymer**	N/A
			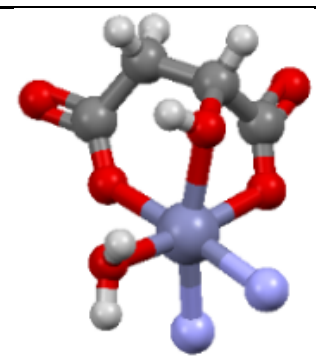 **Mononuclear**	L-mal:2	62.718	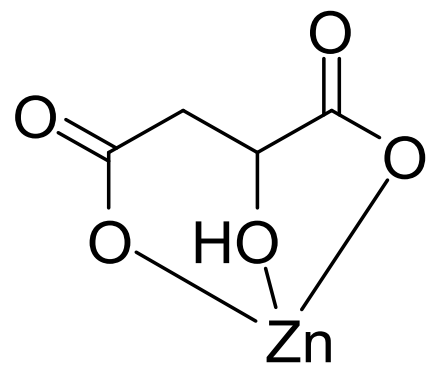 (κ³)	**0D Complex**	N/A

QOCHIA[47]	*P*-1	[Pb(II)(DL-mal)]	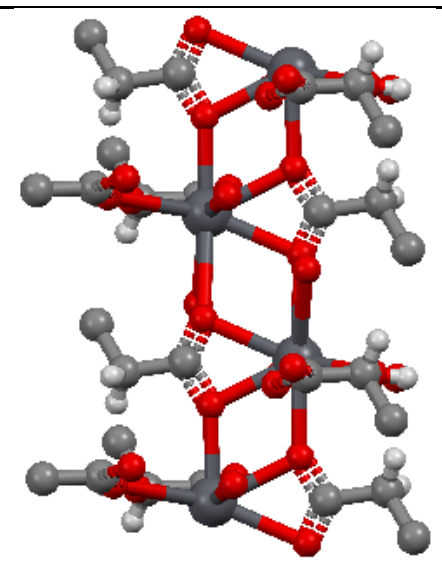<br>**1D RBB**	DL-mal:1	165.774	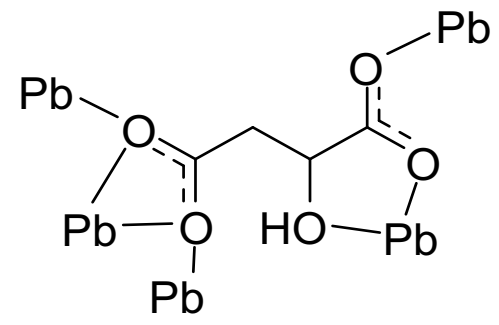<br>(κ^1)-(κ^2)-(κ^1)-(κ^2)-(κ^1)-μ_5	**3D Network**	N/A
QUQSIG[48]	$P2_1/c$	[Ba(II)(DL-mal)]	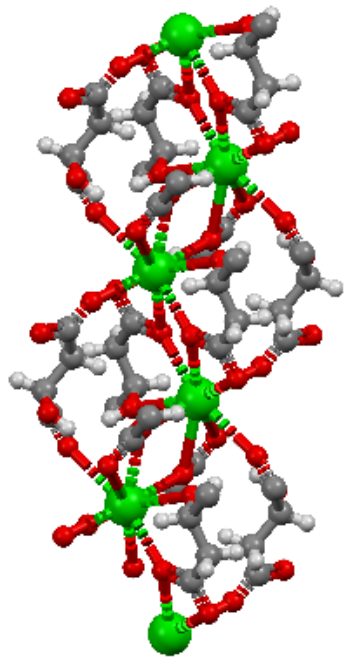<br>**1D RBB**	DL-mal:1	177.364	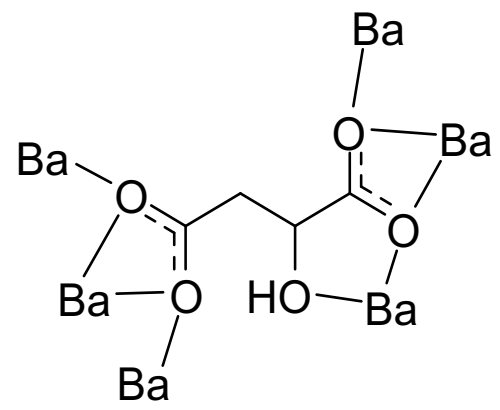<br>(κ^1)-(κ^2)-(κ^1)-(κ^2)-(κ^1)-(κ^1)-μ_6	**3D Network**	N/A
REXBED[49]	*R*3	$[Li(I)_2(L\text{-}mal)]$	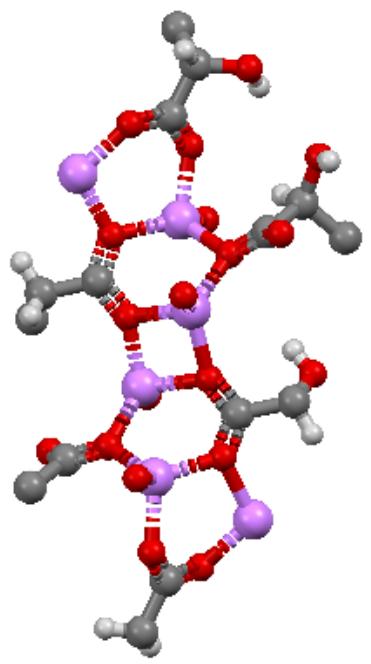<br>1D RBB	L-mal:1	179.76	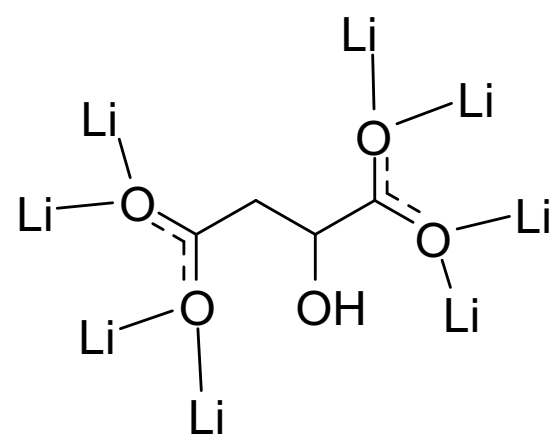<br>(κ^1)-(κ^1)-(κ^1)-(κ^1)-(κ^1)-(κ^1)-(κ^1)-(κ^1)-μ_8	**3D Network**	N/A

ROTPEX[50]	$P2_1/n$	[Ba(II)(DL-mal)$(H_2O)_2$]·H_2O	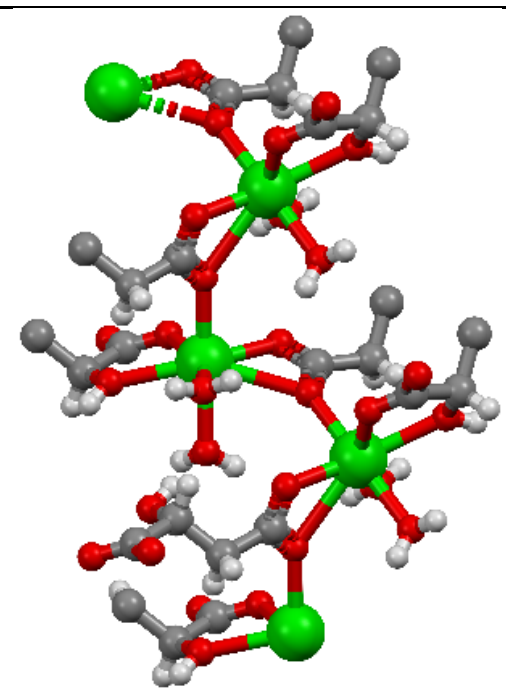 **1D RBB**	DL-mal:1	163.135	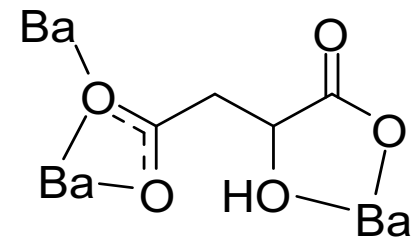 (κ^2)-(κ^2)-(κ^1)-μ_2	**2D Sheet**	N/A
SEJSOQ[51]	$P2_12_12_1$	[Mn(II)(L-mal)(H_2O)]	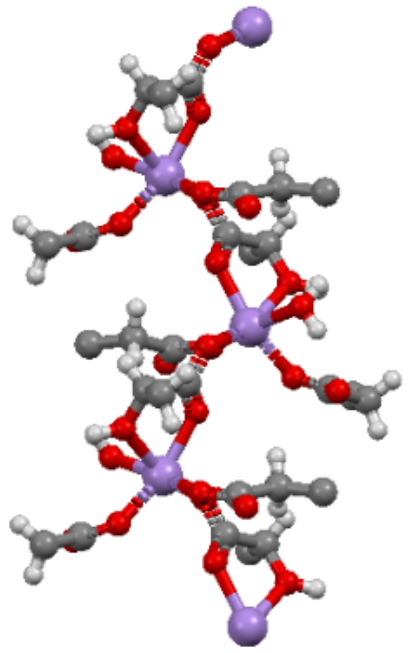 **1D RBB**	L-mal:1	158.006	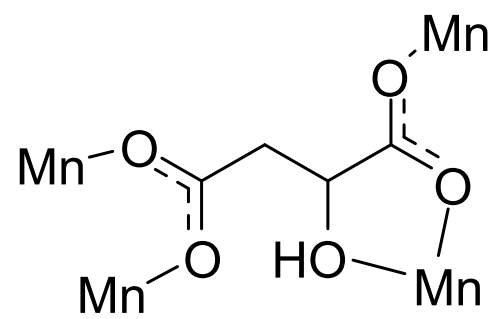 (κ^2)-(κ^1)-(κ^1)-(κ^1)-μ_4	**3D Network**	N/A
SEJSUW[51]	$P2_12_12_1$	[Co(II)(L-mal)(H_2O)]	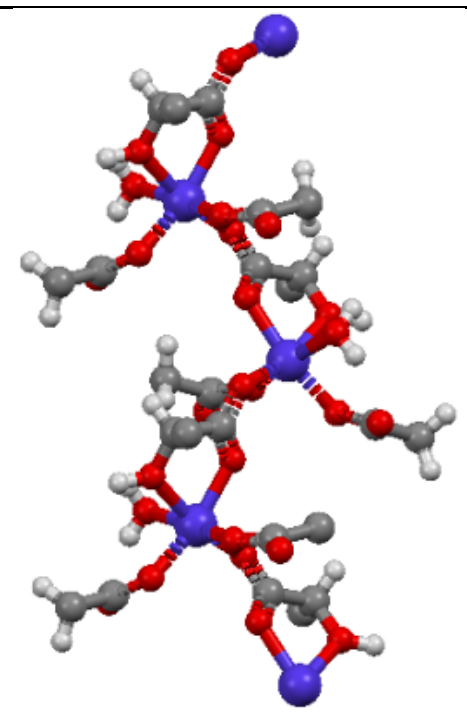 **1D RBB**	L-mal:1	157.935	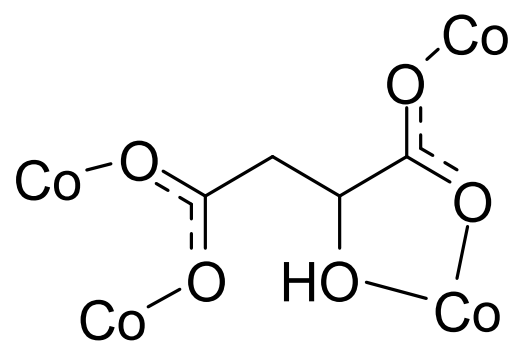 (κ^2)-(κ^1)-(κ^1)-(κ^1)-μ_4	**3D Network**	N/A

SEJTAD[51]	$P2_12_12_1$	[Fe(II)(L-mal)(H_2O)]	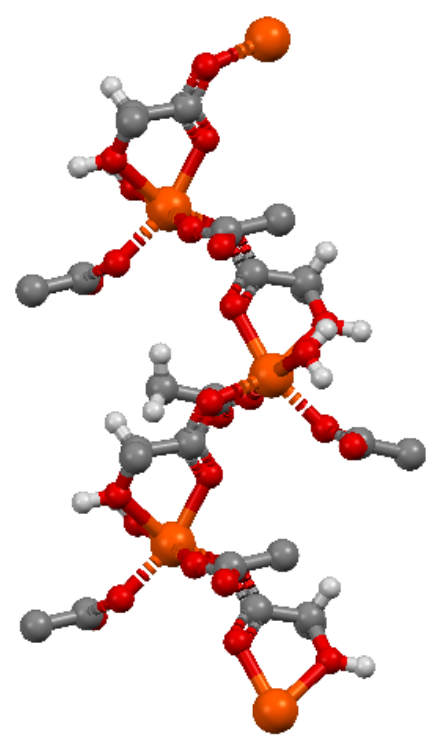<br>**1D RBB**	L-mal:1	157.935	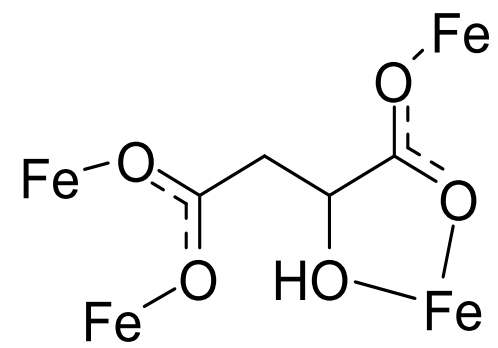<br>(κ^2)-(κ^1)-(κ^1)-(κ^1)-μ_4	**3D Network**	N/A
TECSOJ[52]	$P1$	[Ca(II)$_2$(L-mal)(D,D-tart)(H_2O)$_8$]	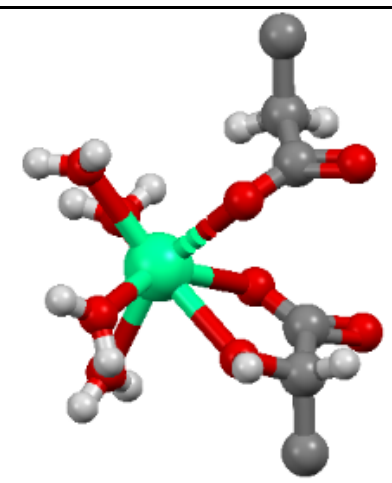<br>**Mononuclear**	L-mal:1	168.693	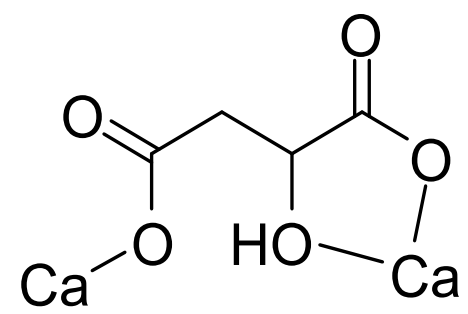<br>(κ^2)-(κ^1)-μ_2	**1D Polymer**	N/A
UGAFIR[53]	$P2_1$	[Ni(II)(L-mal)(H_2O)$_2$]·H_2O	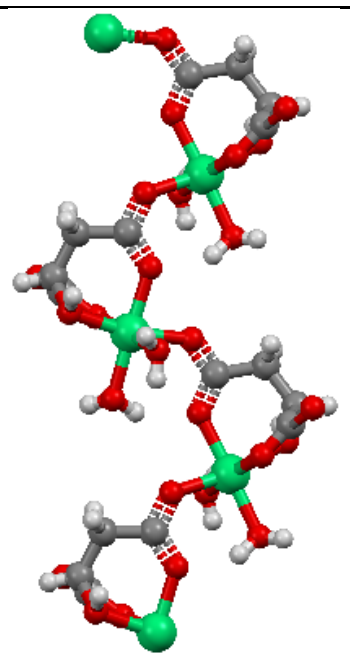<br>**1D RBB**	L-mal:1	64.825	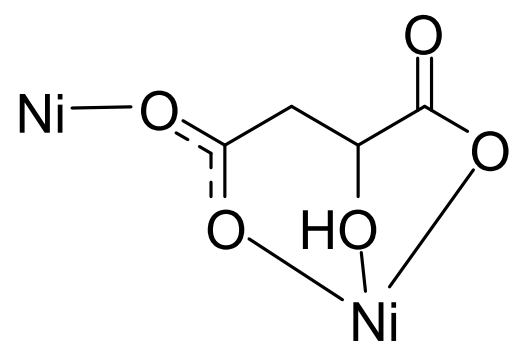<br>(κ^3)-(κ^1)-μ_2	**1D Polymer**	N/A

UZAFAD[54]	*P*-1	$[Ni(II)_3(DL\text{-}mal)_2(fumarate)(tetrakis(Imidazol\text{-}1\text{-}ylmethyl)methane)(H_2O)_2]\cdot 6H_2O$	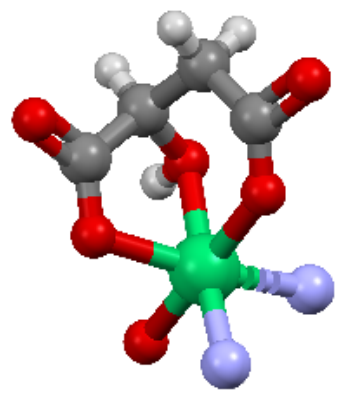 **Mononuclear**	DL-mal:1	60.402	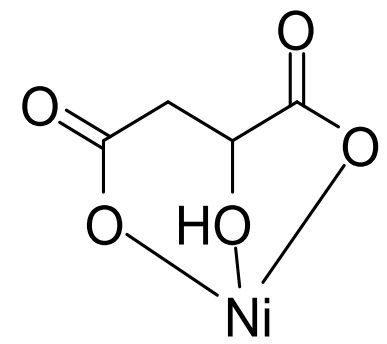 (κ^3)	**0D Complex**	Fumerate 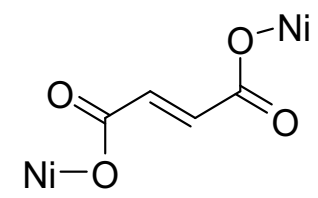 tetrakis(Imidazol-1-ylmethyl)methane 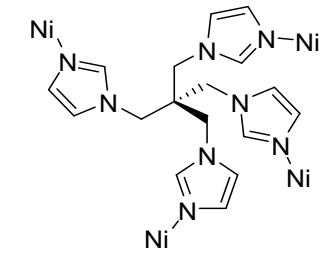 **3D Network**
WIRKER[55]	*Cc*	$[Zn(II)(DL\text{-}mal)(H_2O)]\cdot H_2O$	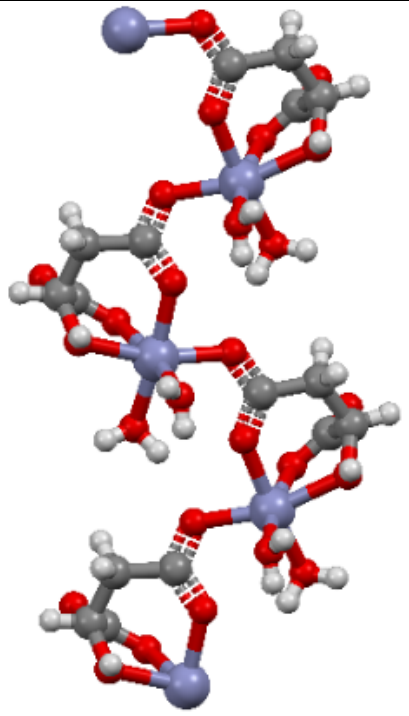 **1D RBB**	DL-mal:1	64.065	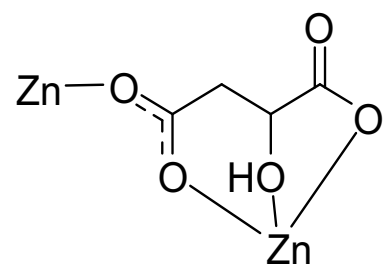 (κ^1)-(κ^3)-(κ^1)-μ_3	**1D Polymer**	N/A
WOYBAQ [56]	*P*-1	$[Gd(III)_2(DL\text{-}mal)_2(ox)(H_2O)_2]\cdot 2H_2O$	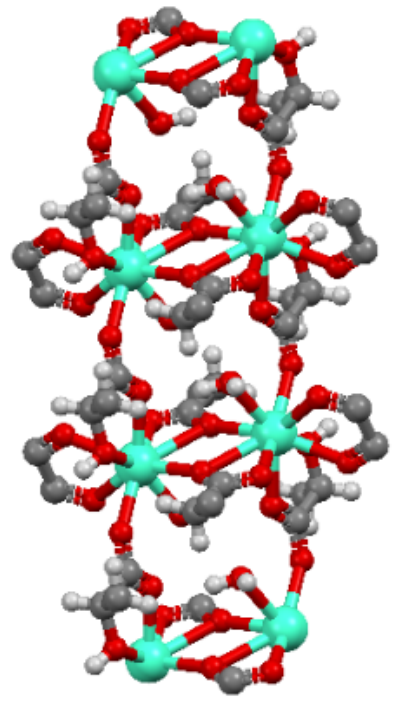 **1D RBB**	DL-mal:1	172.342	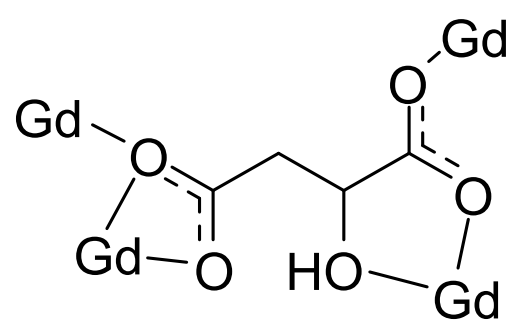 (κ^2)-(κ^1)-(κ^2)-(κ^1)-μ_4	**2D Sheet**	Oxalate 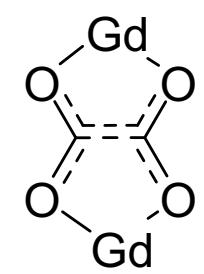 **3D Network**

XEJWUG[57]	$P2_12_12_1$	[Co(II)$_2$(L-mal)$_2$)(4,4'bipy)]·4H_2O	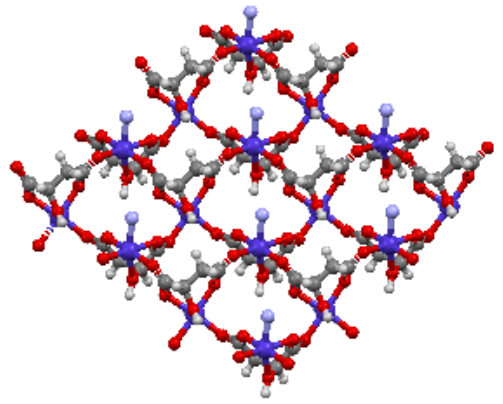 **2D Layer**	L-mal:1	66.929	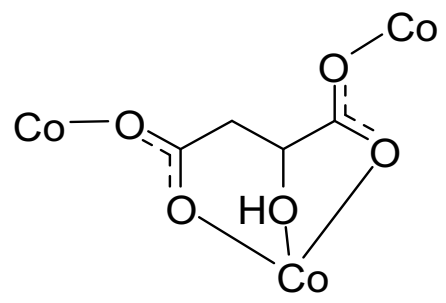 (κ^1)-(κ^3)-(κ^1)-μ$_3$	**2D Sheet**	4,4'-bipyridine 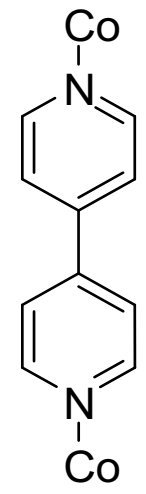 **3D Network**
XEJXAN[57]	$P2_12_12_1$	[Ni(II)$_2$(L-mal)$_2$(4,4'bipy)]	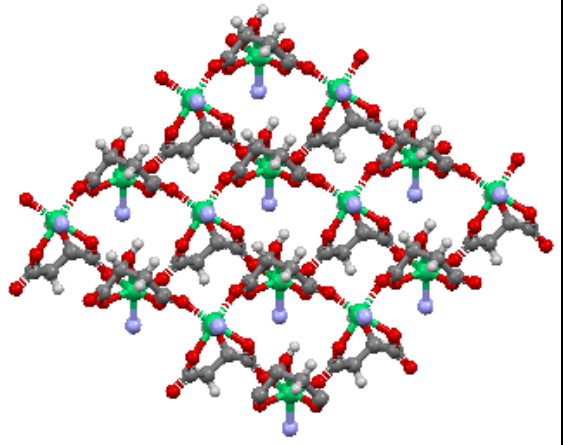 **2D Layer**	L-mal:1	67.098	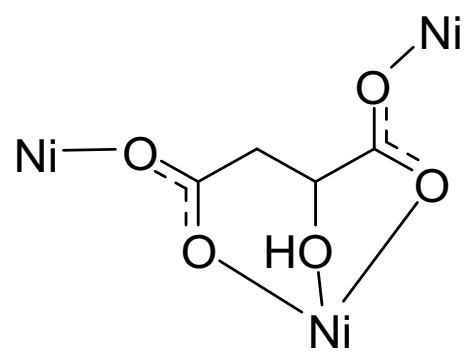 (κ^1)-(κ^3)-(κ^1)-μ$_3$	**2D Sheet**	4,4'-bipyridine 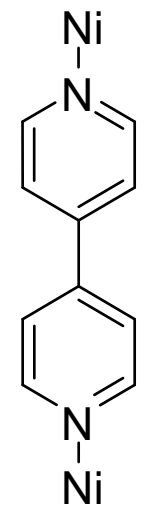 **3D Network**
XEJXER[57]	$P2_12_12_1$	[Ni(II)$_2$(L-mal)$_2$(4,4'-ethene-1,2-diyldipyridine)]·2H_2O	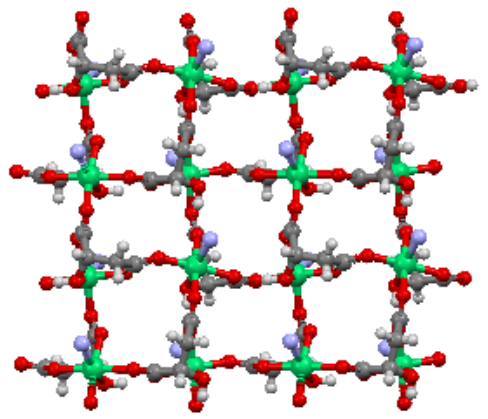 **2D Layer**	L-mal:1	53.171	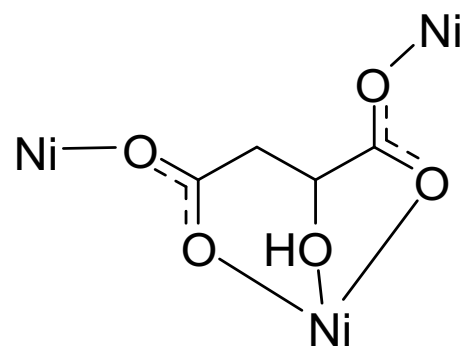 (κ^1)-(κ^3)-(κ^1)-μ$_3$	**2D Sheet**	4,4'-ethene-1,2-diyldipyridine 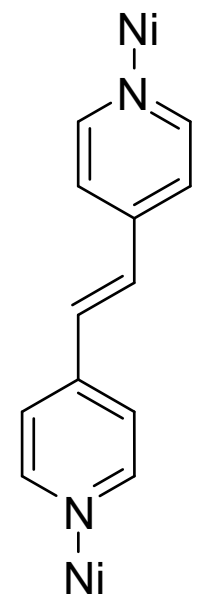 **3D Network**
				L-mal:2	64.137	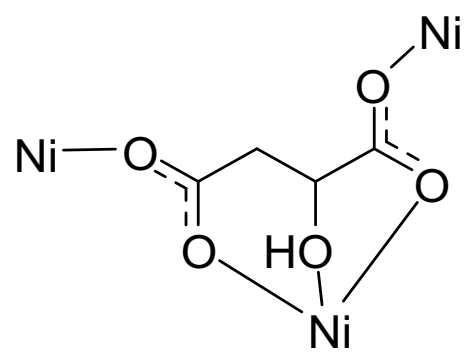 (κ^1)-(κ^3)-(κ^1)-μ$_3$		

XIRFIO[58]	*Pbca*	[Fe(II)(DL-mal)(H_2O)]·$2H_2O$	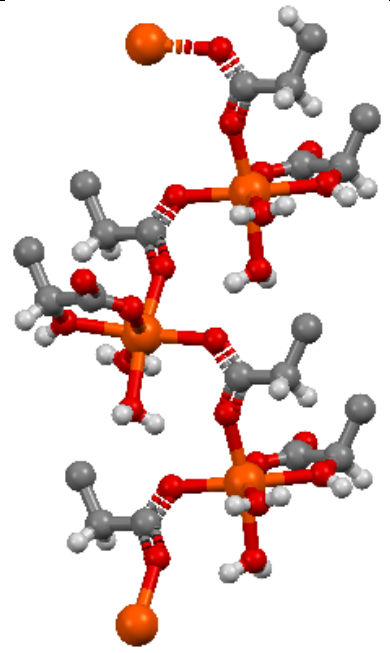 **1D RBB**	L-mal:1	172.178	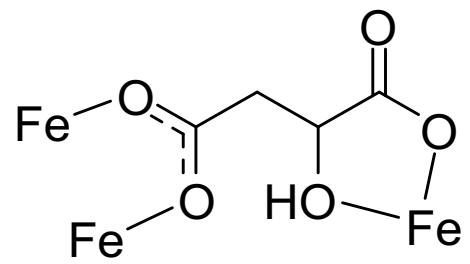 (κ^2)-(κ^1)-(κ^1)-μ_3	**2D Sheet**	N/A
YORJUL[59]	*Fdd*2	[Ni(II)(DL-mal)(1,2-bis(4-pyridyl)ethane)]·$5H_2O$	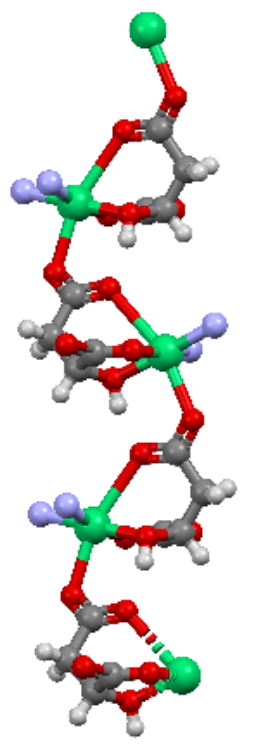 **1D RBB**	DL-mal:1	64.683	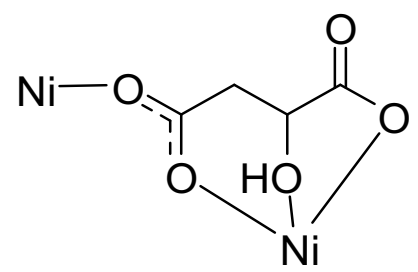 (κ^3)-(κ^1)-μ_2	**1D Polymer**	1,2-bis(4-pyridyl)ethane 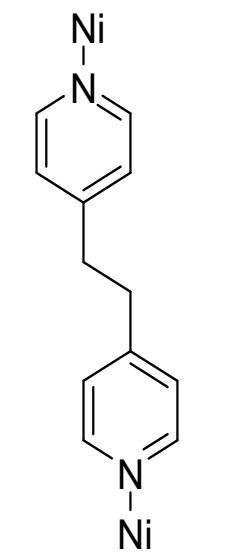 **2D Sheet**
YORKOG[59]	*Pccn*	[Ni(II)(DL-mal)(bispyridylpropane]]·$6H_2O$	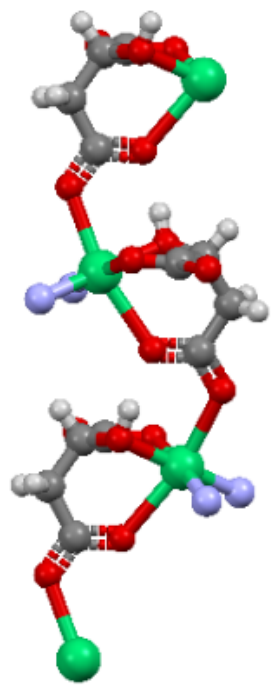 **1D RBB**	DL-mal:1	61.49	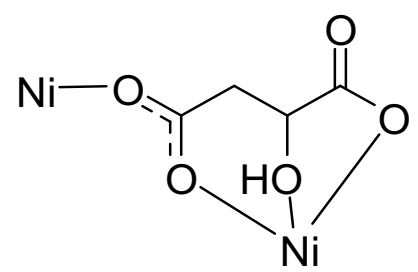 (κ^3)-(κ^1)-μ_2	**1D Polymer**	bis(1,3-bis(4-pyridyl)propane 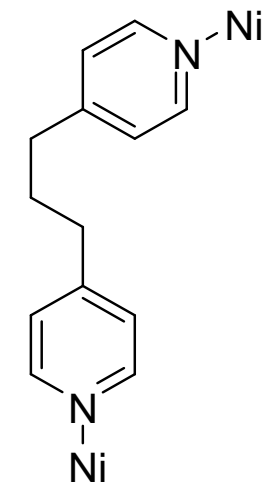 **3D Network**

ZUHRON [60]	$P2_12_12_1$	[Li(I)(L-mal)(H_2O)$_2$](Him)	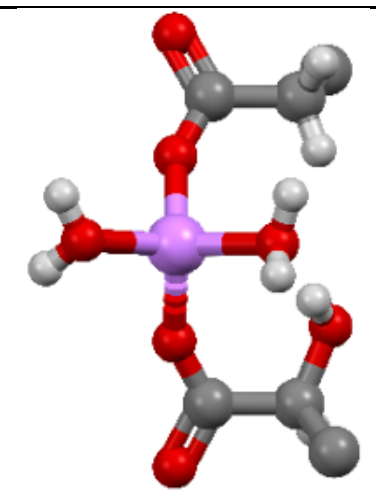 **Mononuclear**	L-mal:1	179.518	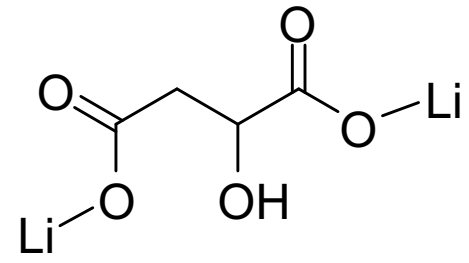 (κ^1)-(κ^1)-μ_2	**1D Polymer**	N/A

S2.1 Hard Soft Acid Base Parameters for Metal Ions

Table S2.2: Ionic radius and hardness parameters for the monovalent, divalent and trivalent metal ions discussed. Values sourced from [61].

Monovalent Metal Ion	Ionic Radius (Å)	Hardness Parameter	Divalent Metal Ion	Ionic Radius (Å)	Hardness Parameter	Trivalent Metal Ion	Ionic Radius (Å)	Hardness Parameter
Li(I)	0.76	0.247	Mg(II)	0.72	0.167	In(III)	0.80	0.179
K(I)	1.38	0.232	Ca(II)	0.99	0.181	Pr(III)	1.01	0.155
Rb(I)	1.52	0.221	Mn(II)	0.80	0.125	Nd(III)	1.00	0.154
Ag(I)	1.00	0.074	Fe(II)	0.65	0.107	Eu(III)	0.95	0.153
Cs(I)	1.67	0.218	Co(II)	0.75	0.130	Gd(III)	0.94	0.150
			Ni(II)	0.69	0.126			
			Cu(II)	0.73	0.104			
			Zn(II)	0.72	0.115			
			Cd(II)	0.97	0.081			
			Ba(II)	1.35	0.183			
			Pb(II)	1.19	0.131			

S2.2 C4 Dicarboxylate Conformation

Torsion angles for the aliphatic backbone of succinate and tartrate was taken from the Cambridge Structural Database[2] (CSD, v.6.01; database updates: April 2026) MOF Subset, accessed using ConQuest.[3]

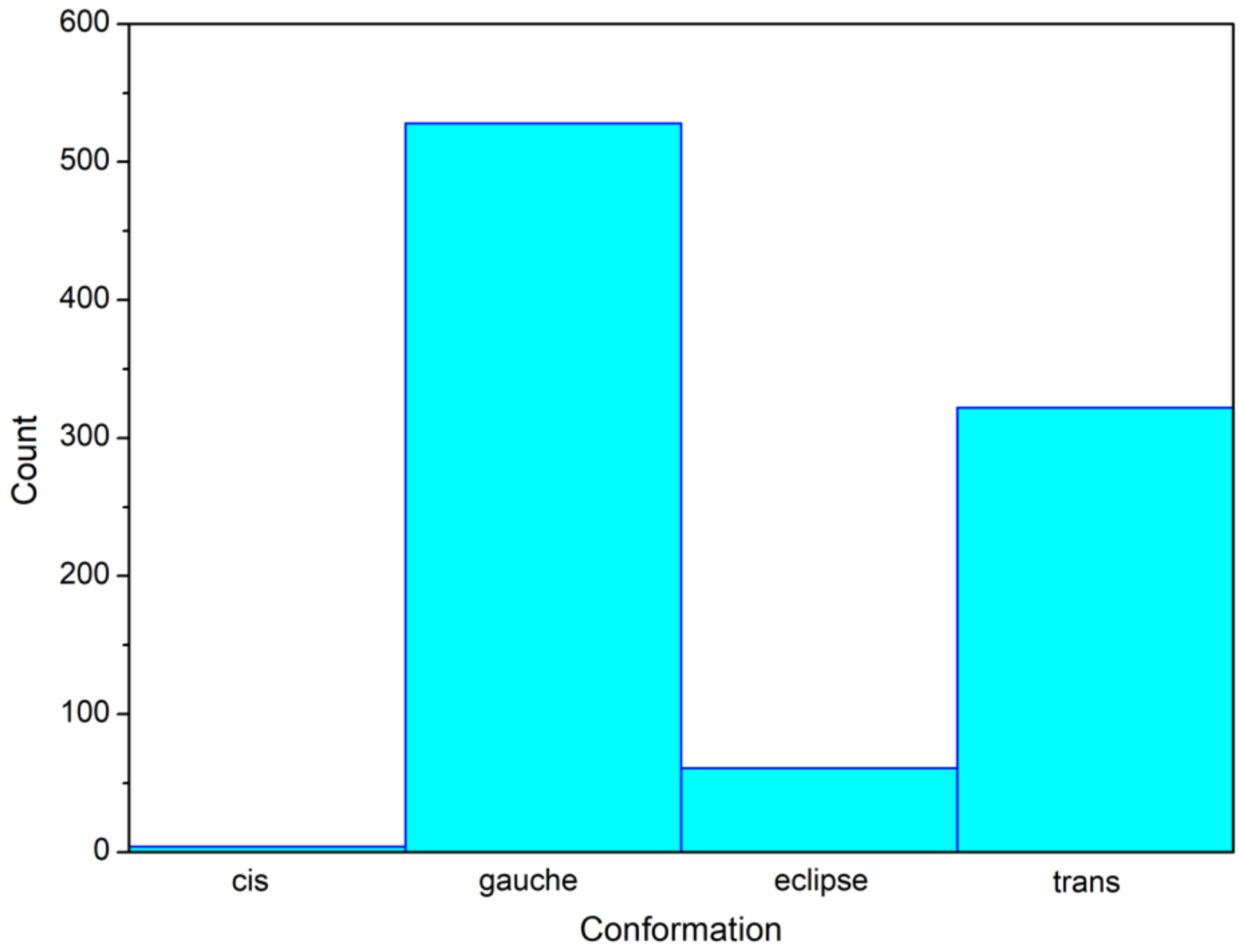

Figure S2.1: Population of the conformation of the aliphatic backbone of succinate.

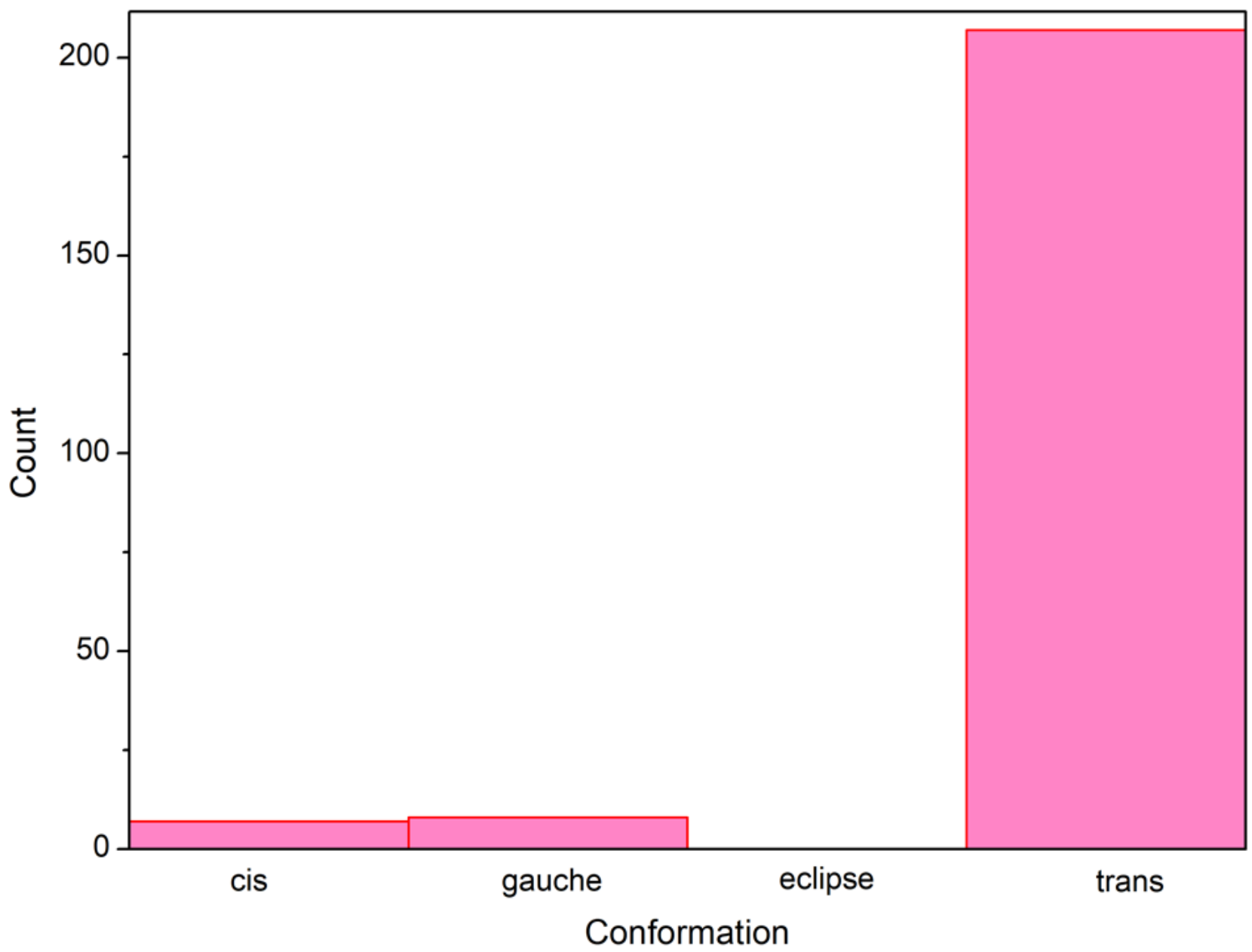

Figure S2.2: Population of the conformation of the aliphatic backbone of tartrate.

S3. Powder X-ray Diffraction

High-quality single crystals of **Mg-Aqua-DL-mal**, **Ca-Aqua-DL-mal**, **Mn-Aqua-DL-mal**, **Fe-Bzim-DL-mal**, **Co-Bzim-DL-mal** and **Ni-Bzim-DL-mal** were chosen for single-crystal X ray diffraction measurements and diffraction data were collected on Bruker Quest diffractometer equipped with a IμSmicrofocus X-ray source (Mo Kα, λ = 0.71073 Å, Cu Kα, λ = 1.54178 Å) and CMOS detector. Data reduction was carried out using the Bruker SAINT3 software. Absorption correction was performed by multi-scan method using in SADABS.[62] Data was indexed, integrated, and scaled in APEX4.[63, 64]

Space groups were determined using XPREP incorporated within APEX4. Structure solution, refinement, and data output were carried out using OLEX 2 with the inbuilt SHELXTL-2018 program.[65, 66] Non-hydrogen atoms were refined anisotropically. Hydrogen atoms were placed using molecular geometry at idealised locations and assigned isotropic thermal parameters depending on the equivalent displacement parameters of their carriers.

S4.1 Crystallographic Tables

	**Mg-Aqua-DL-mal**	**Ca-Aqua-DL-mal**	**Mn-Aqua-DL-mal**
Empirical Formula	$C_4H_{10}MgO_8$	$C_4H_{12}CaO_9$	$C_4H_8MnO_8$
MM(g mol^{-1})	210.43	244.22	239.04
System	Monoclinic	Monoclinic	Orthorhombic
Sp. gr.	*P*2$_1$/*c*	*P*2$_1$/*c*	*Pbca*
a (Å)	9.8211(4)	23.314(4)	14.2154(5)
b (Å)	7.0696(3)	6.6014(8)	8.2679(3
c (Å)	11.8188(4)	14.524(2)	14.6911(5)
β (°)	98.596(2)	119.801(7)	90
Vol. (Å^3)	811.38(6)	1939.7(5)	1726.67(11)
Z, Z′	4, 1	8, 1	8, 1
ρ (g cm^{-3})	1.654	1.6725	1.839
Temp. (K)	295	301	300
Refl. measu.	21446	5480	20657
Refl. indep.	1665	1906	1766
Obs I > 2σ	1330	976	1341
R_{int}	0.1160	0.1192	0.1340
R_1^a (%)	4.36	12.50	4.86
μ (mm^{-1})	2.229	6.391	39.135
Radiation type	Cu *Kα*	Cu *Kα*	Cu *Kα*
$^aR_1 = \sum\|\|F_o\| - \|F_c\|\|/\sum\|F_o\|$			

	**Fe-Bzim-DL-mal**	**Co-Bzim-DL-mal**	**Ni-Bzim-DL-mal**
Empirical Formula	$C_{80}H_{88}Fe_4N_{16}O_{24}$	$C_{80}H_{88}Co_4N_{16}O_{32}$	$C_{72}H_{64}N_{16}Ni_4O_{20}$
MM(g mol^{-1})	1881.06	2037.51	1708.15
System	Tetragonal	Tetragonal	Tetragonal
Sp. gr.	*P*4/*n*	*P*4/*n*	*P*4/*n*
a (Å)	20.3861(9)	20.2504(4)	20.0530(7)
b (Å)	20.3861(9	20.2504(4)	20.0530(7)
c (Å)	11.1943(9)	11.5658(4)	11.6480(6)
β (°)	90	90	90
Vol. (Å^3)	4652.3(6)	4742.9(2)	4683.9(4)
Z, Z′	2, 0.25	2, 0.25	2, 0.25

ρ (g cm^{-3})	1.343	1.427	1.211
Temp. (K)	296	150	296
Refl. measu.	49119	59164	77992
Refl. indep.	4786	5465	4867
Obs I > 2σ	2666	3491	4213
R_{int}	0.1729	0.1729	0.2514
$R_1{}^a$ (%)	6.34	8.36	6.75
μ (mm^{-1})	5.574	0.775	1.469
Radiation type	Cu *Kα*	Mo *Kα*	Cu *Kα*
$^aR_1 = \sum \| F_o\| - \|F_c\| / \sum\|F_o\|$			

S4. Powder X-ray Diffraction

Powder X-ray diffraction (PXRD) experiments were conducted using microcrystalline samples on a PANalytical Empyrean diffractometer (40 kV; 40 mA; CuK$\alpha_{1,2}$ λ = 1.5418 Å) in Bragg–Brentano geometry. Instrument details: reflection transmission spinner configuration, divergence slit = 1/8°, Soller slit = 0.04 rad, Ni filter, PIXcel-1D detector. Data were collected with a step size of 0.0131303 at a count time of 80 s per step over the range 5° < 2θ < 40°. PXRD patterns were also calculated from SCXRD structures using Mercury software.[67]

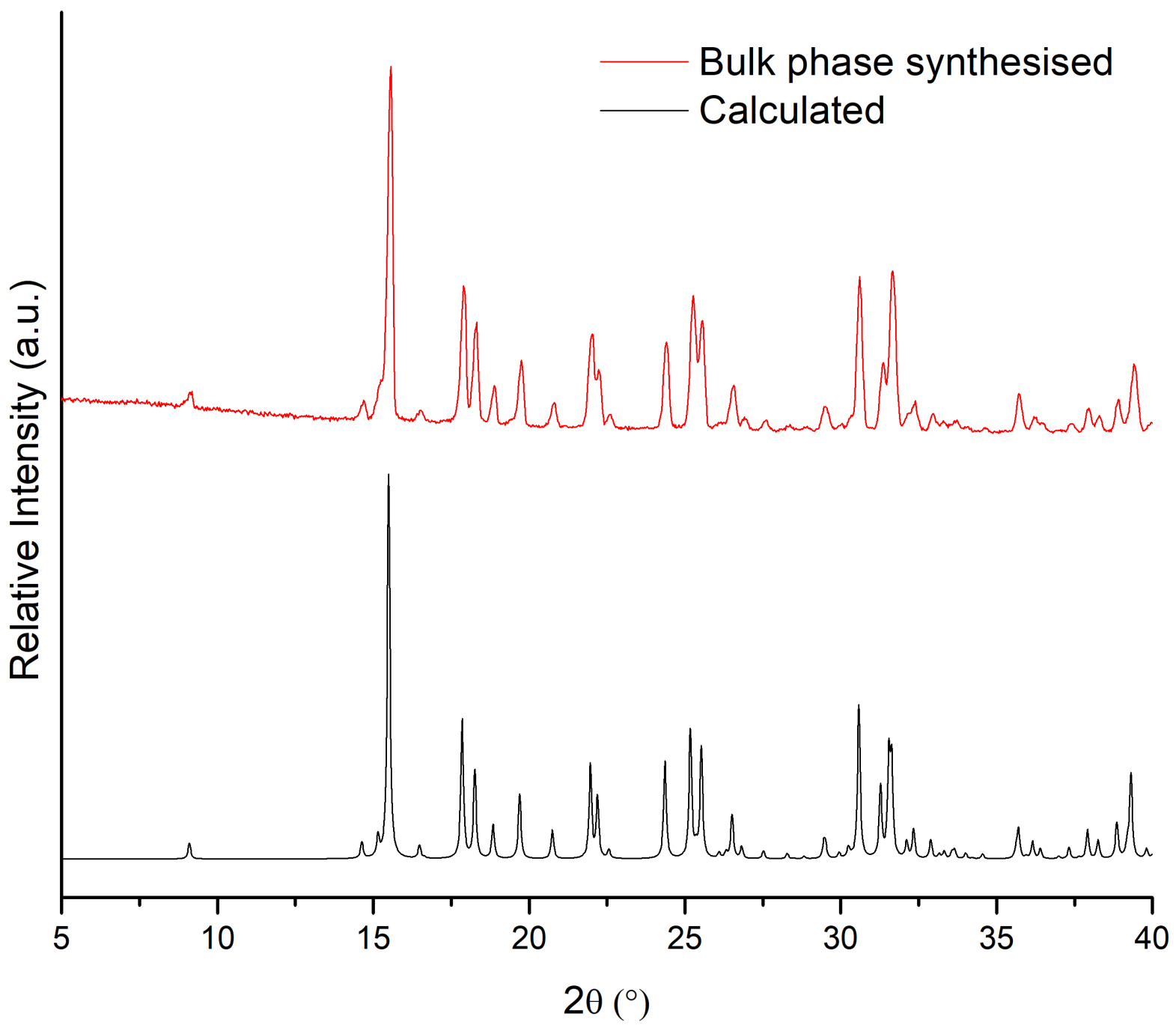

Figure S4.1:PXRD Plot of **Mg-Aqua-DL-mal**.

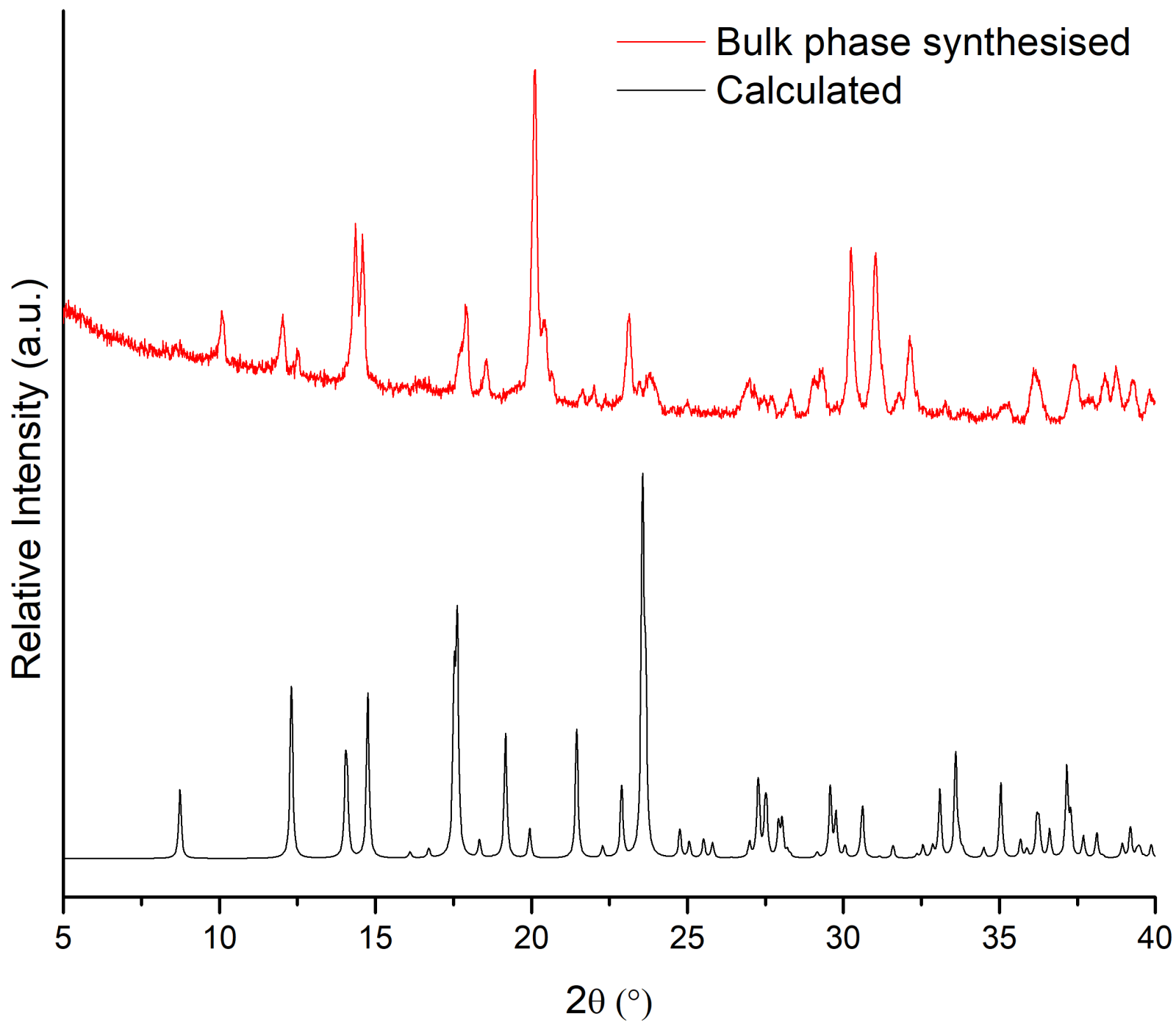

Figure S4.2:PXRD Plot of **Ca-Aqua-DL-mal**.

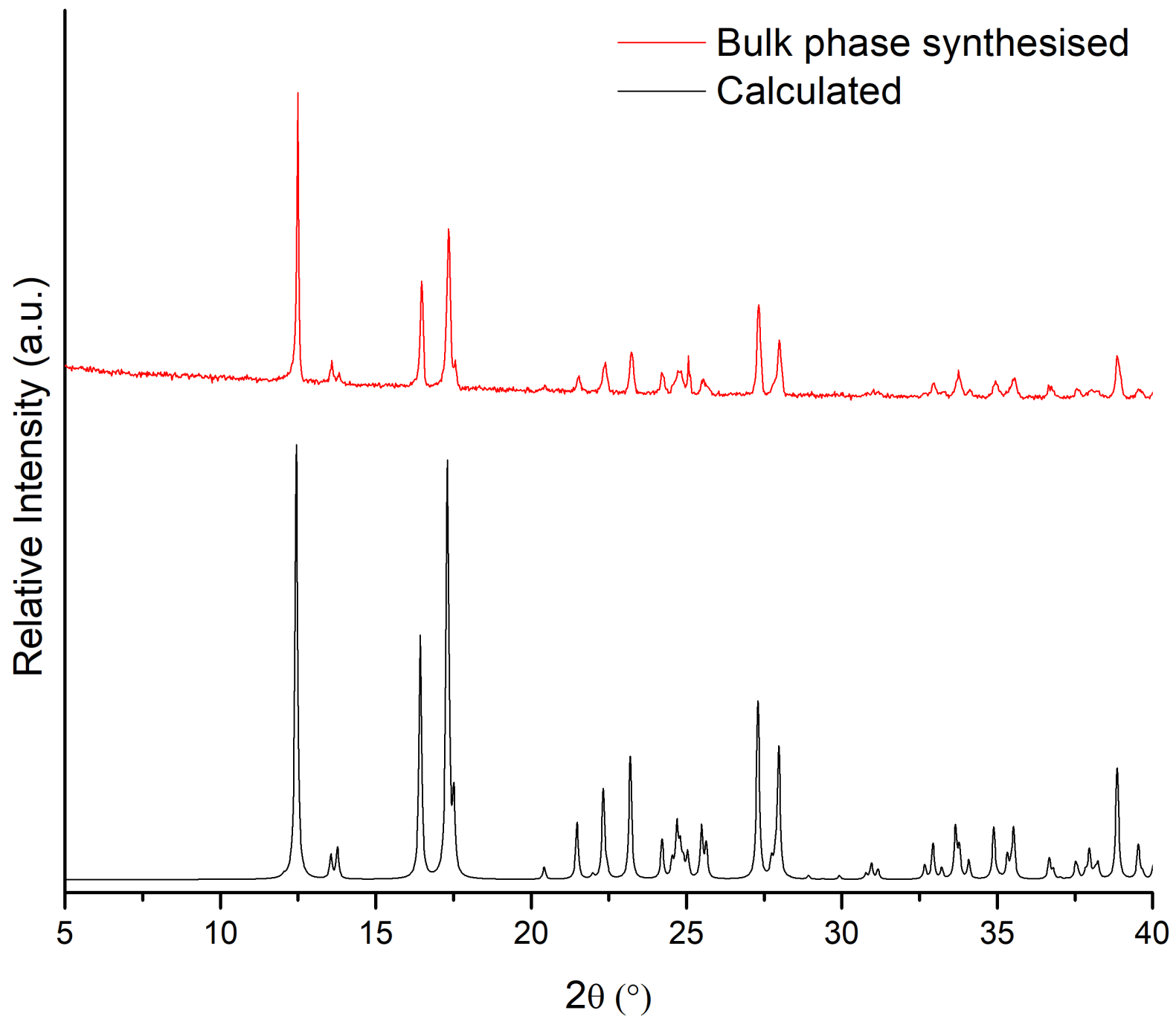

Figure S4.3:PXRD Plot of **Mn-Aqua-DL-mal**.

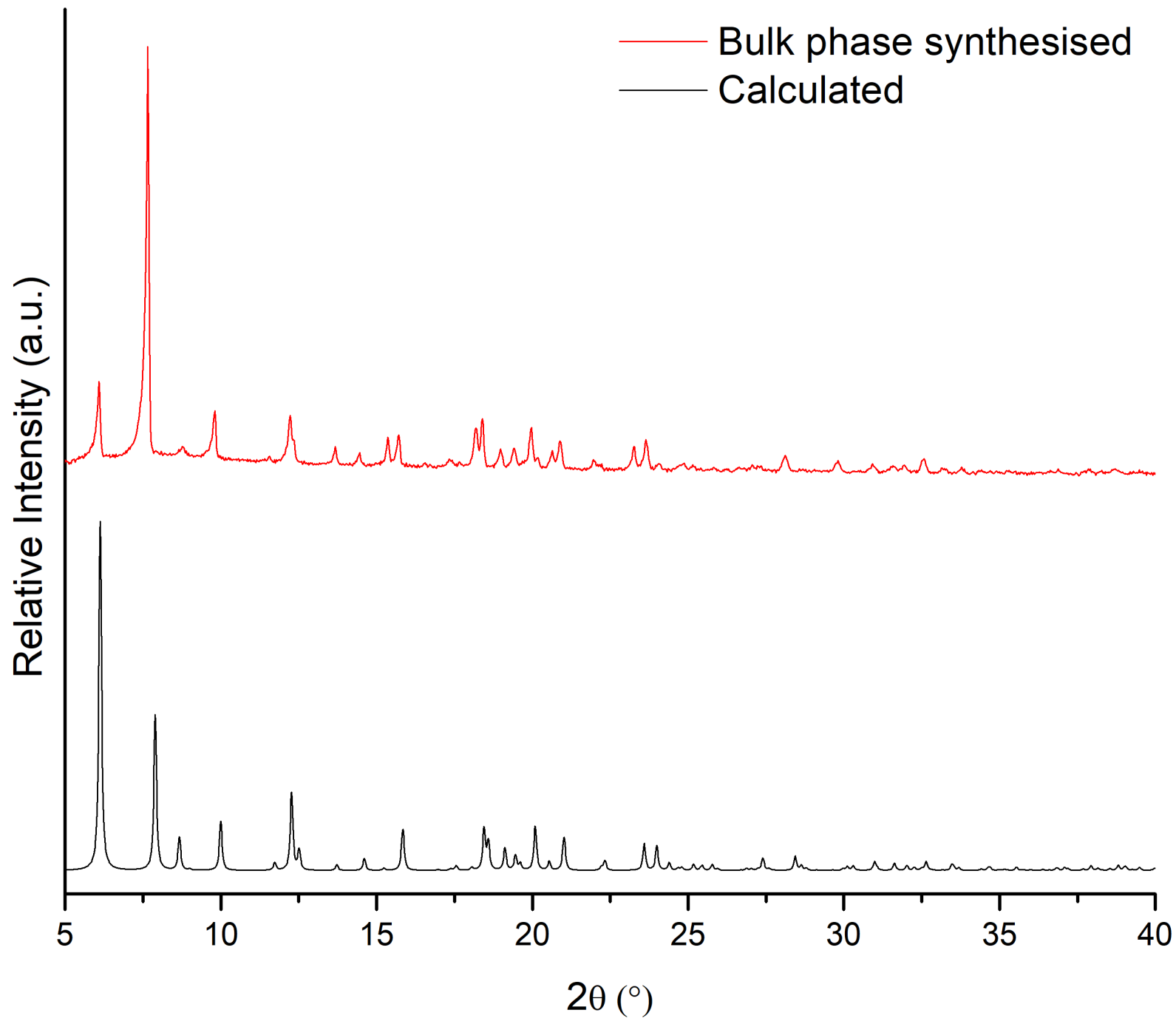

Figure S4.4:PXRD Plot of **Fe-Bzim-DL-mal**.

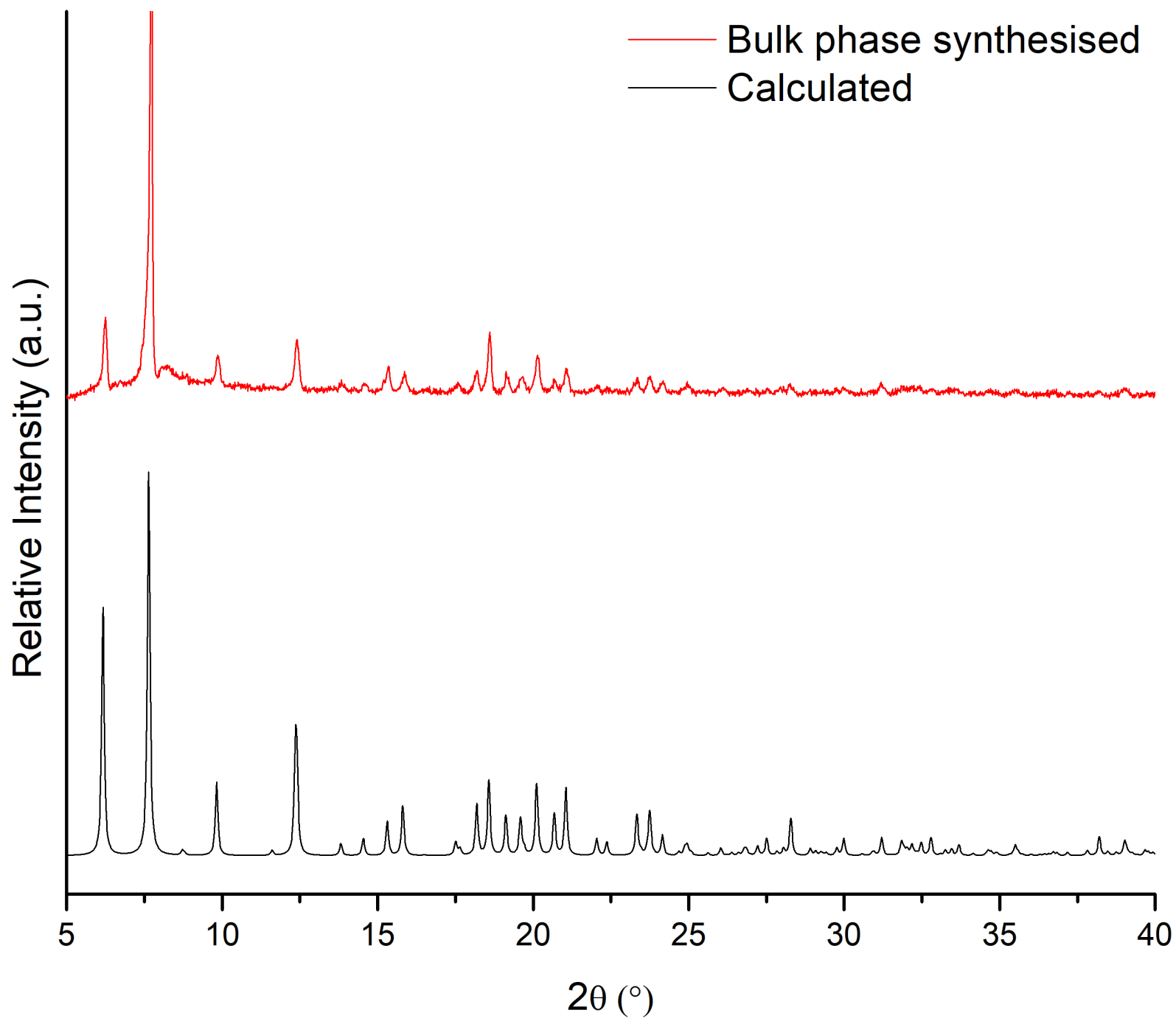

Figure S4.5:PXRD Plot of **Co-Bzim-DL-mal**.

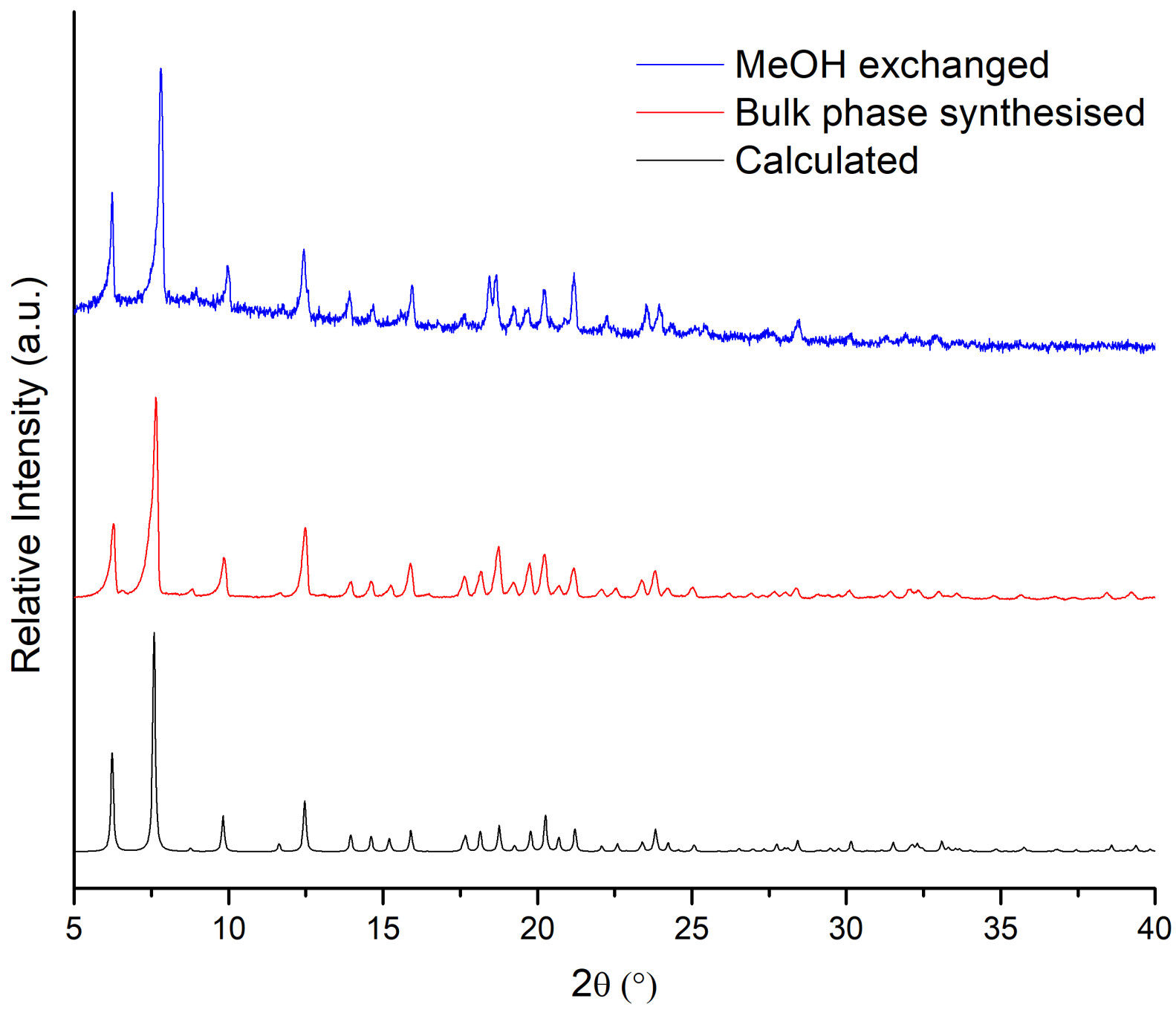

Figure S4.6:PXRD Plot of **Ni-Bzim-DL-mal**.

S5. Variable Temperature PXRD

Diffractograms at different temperatures for Methanol exchanged **Ni-Bzim-DL-mal** were recorded using a PANalytical X'Pert Pro-MPD diffractometer equipped with a PIXcel3D detector operating in scanning line detector mode. Anton Paar TTK 450 stage coupled with the Anton Paar TCU 110 Temperature Control Unit was used to record the variable temperature diffractograms. The diffractometer is outfitted with an Empyrean Cu LFF (long fine focus) HR (9430 033 7300x) tube operated at 40 kV and 40 mA and Cu Kα radiation (λ = 1.54056 Å) was used for diffraction experiments. Continuous scanning mode with the goniometer in the theta-theta orientation was used to collect the data. Incident beam optics included the Fixed Divergences slit, with a 1/4° divergence slit and a Soller slit (0.04 rad). The data was collected from 5°-60° (2θ) with a step-size of 0.0167113° and a scan time of 130 seconds per step. Divergent beam optics included a P7.5 S7 anti-scatter slit, a Soller slit (0.04 rad), and a Ni-β filter. Data were collected on approximately 15 mg of material and after each temperature ramp the sample was allowed 30 minutes to equilibrate before data collection.

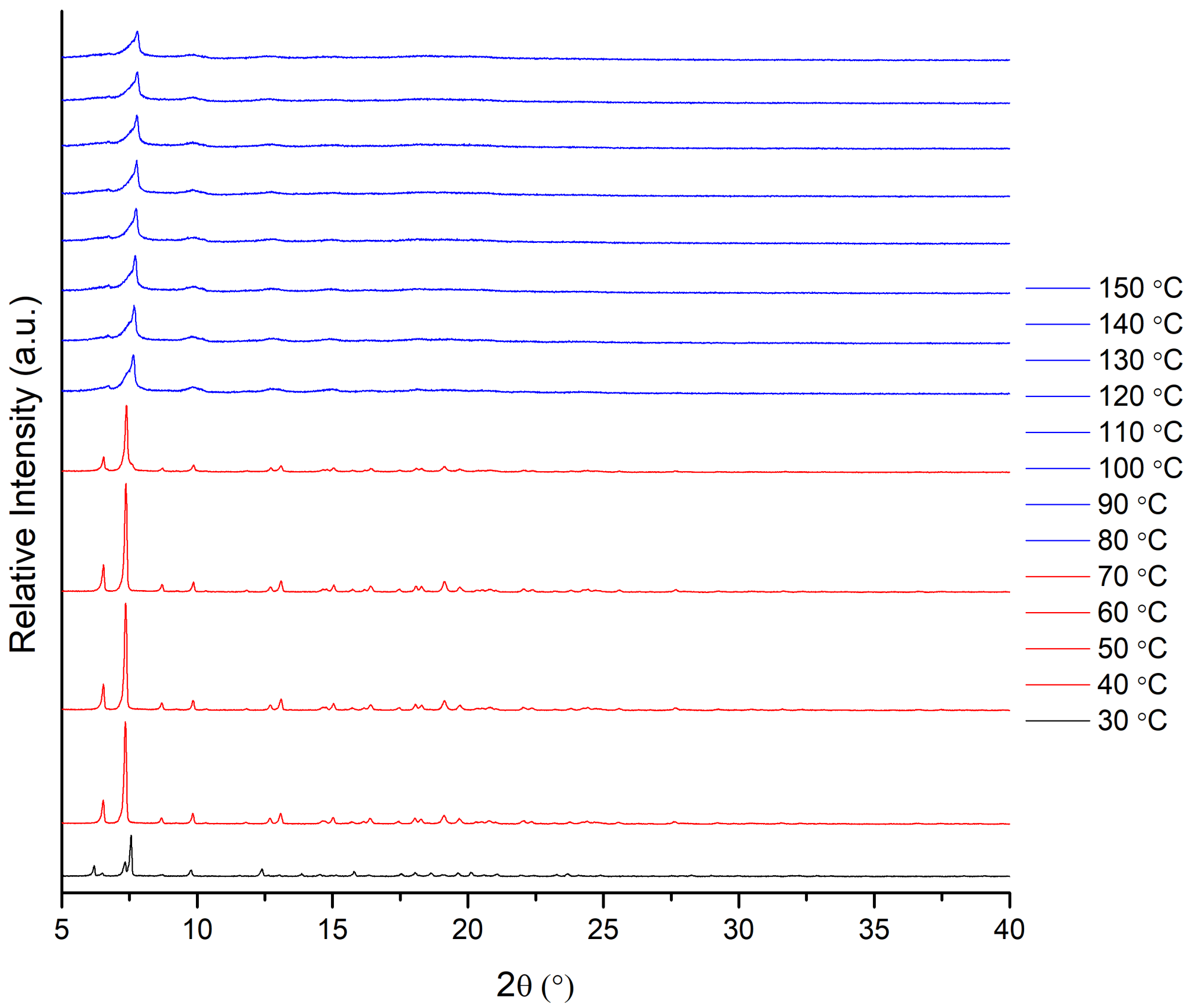

Figure S5.1: VT-PXRD Plot of MeOH exchanged **Ni-Bzim-DL-mal**.

S6. Thermogravimetric analysis

Thermogravimetric analyses were performed under N_2 flow using a TA Instruments Q50 system. A sample was loaded into an aluminium sample pan and heated at 10 °C min^{-1} from room temperature to 500 °C. Methanol exchanged **Ni-Bzim-DL-mal** was instead heated at 1 °C min^{-1} from room temperature to 250 °C.

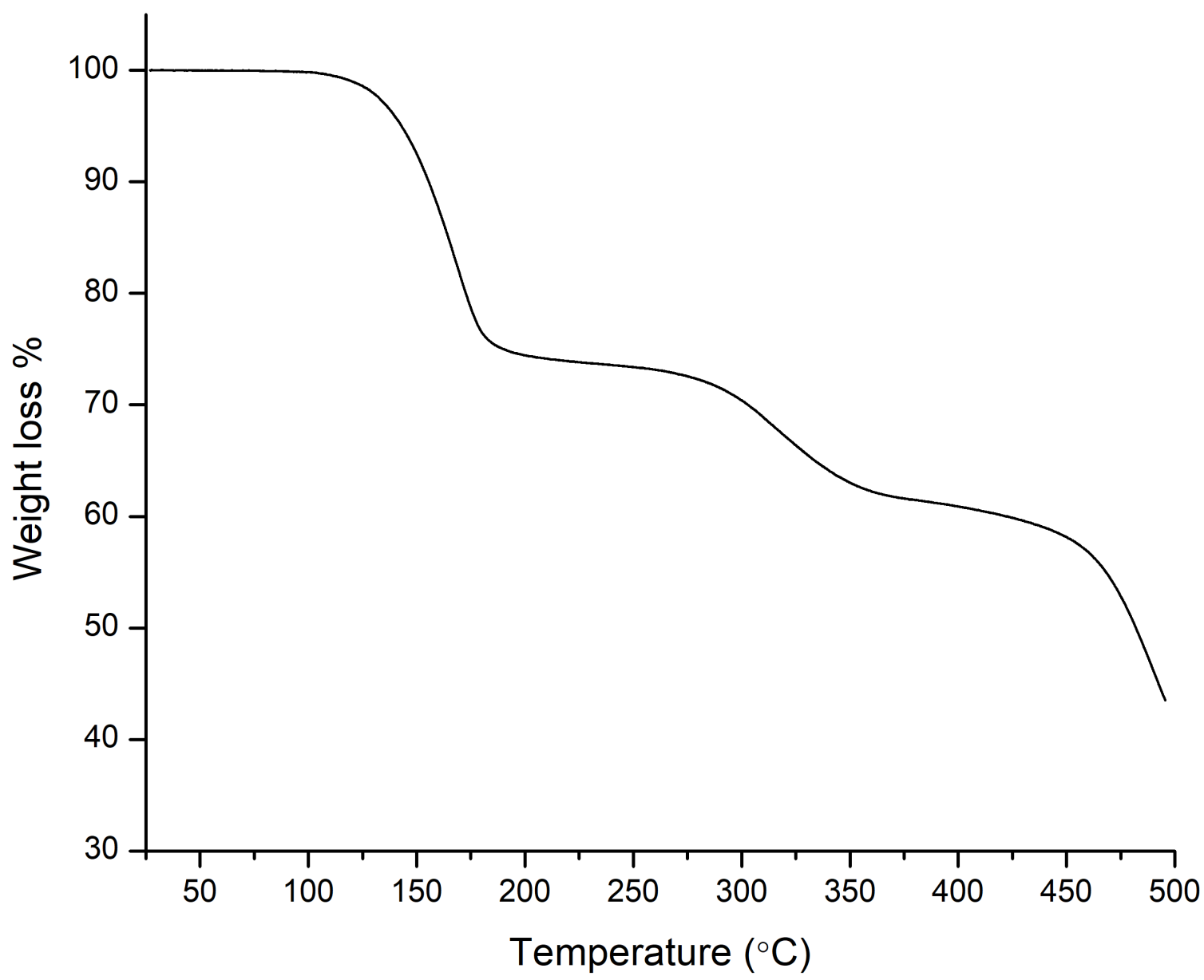

Figure S6.1: TGA thermogram of **Mg-Aqua-DL-mal**

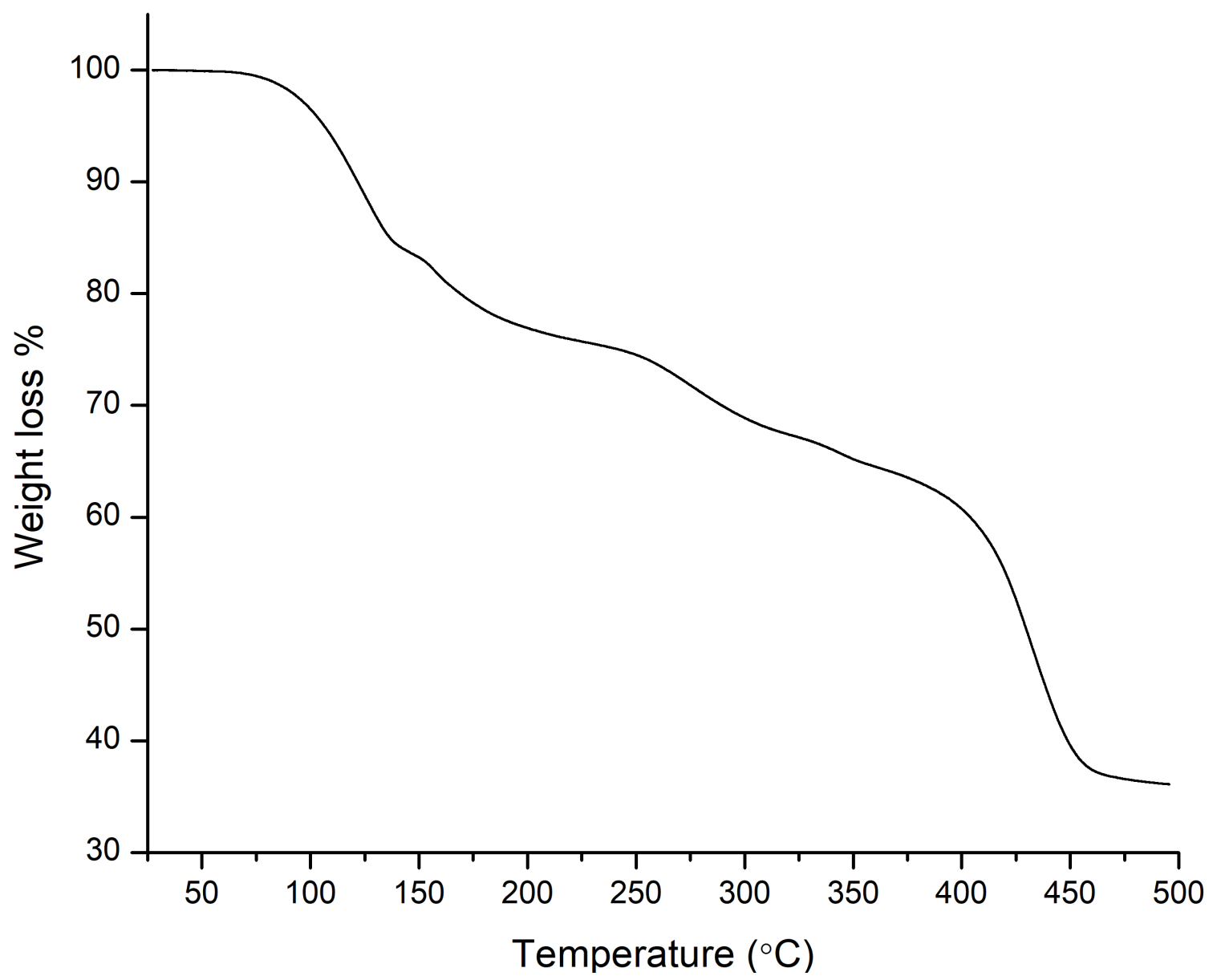

Figure S6.2: TGA thermogram of **Mn-Aqua-DL-mal**.

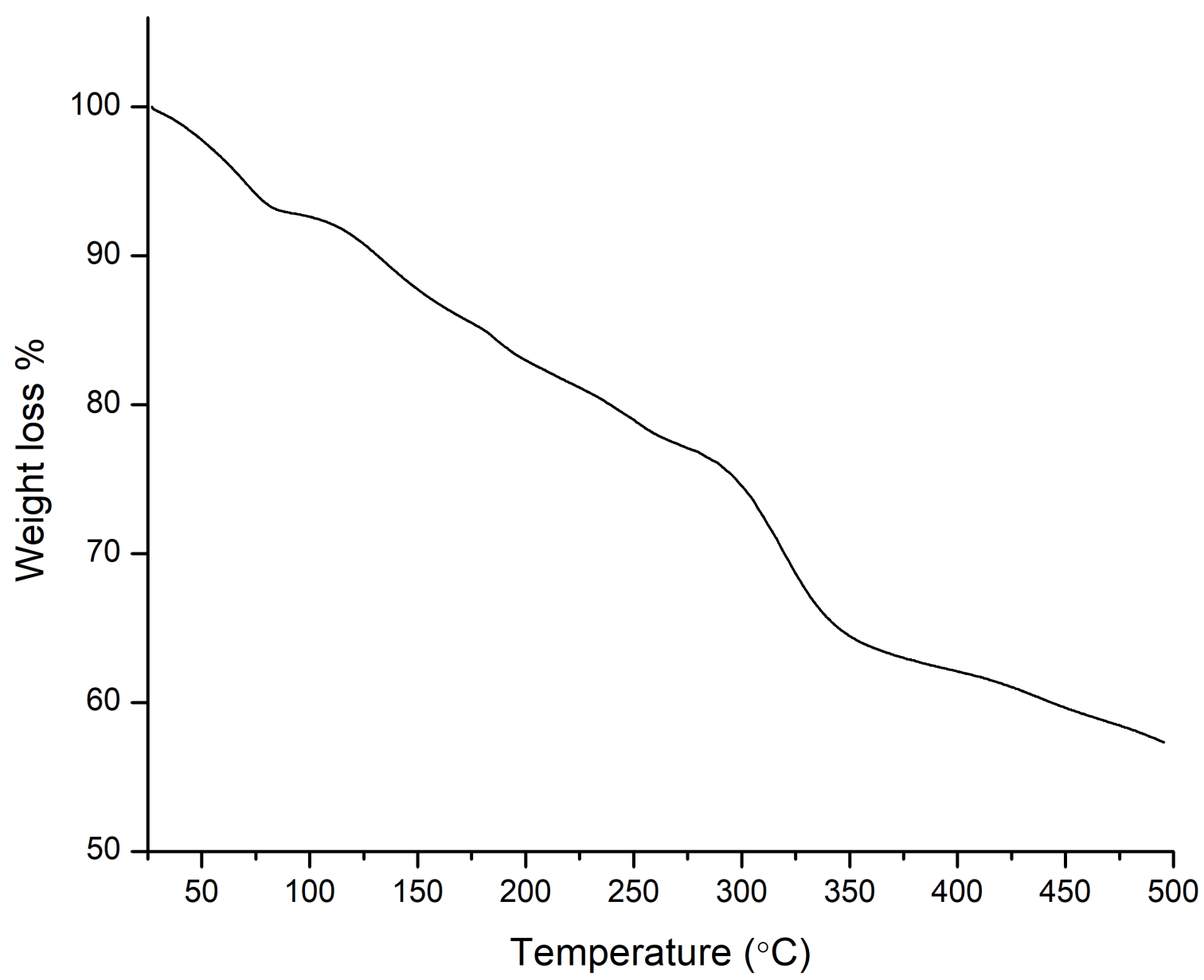

Figure S6.3: TGA thermogram of **Fe-Bzim-DL-mal**.

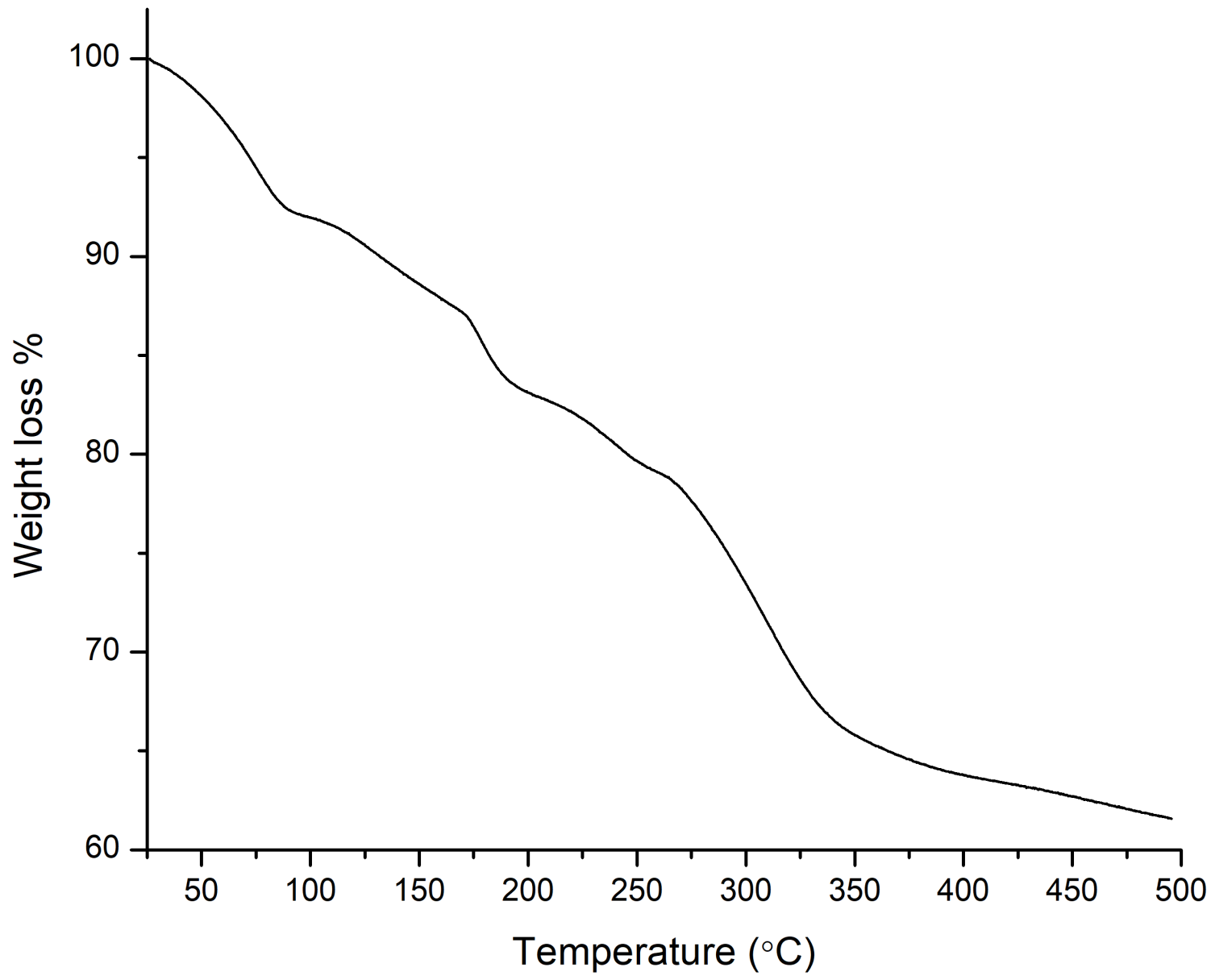

Figure S6.4: TGA thermogram of **Co-Bzim-DL-mal**.

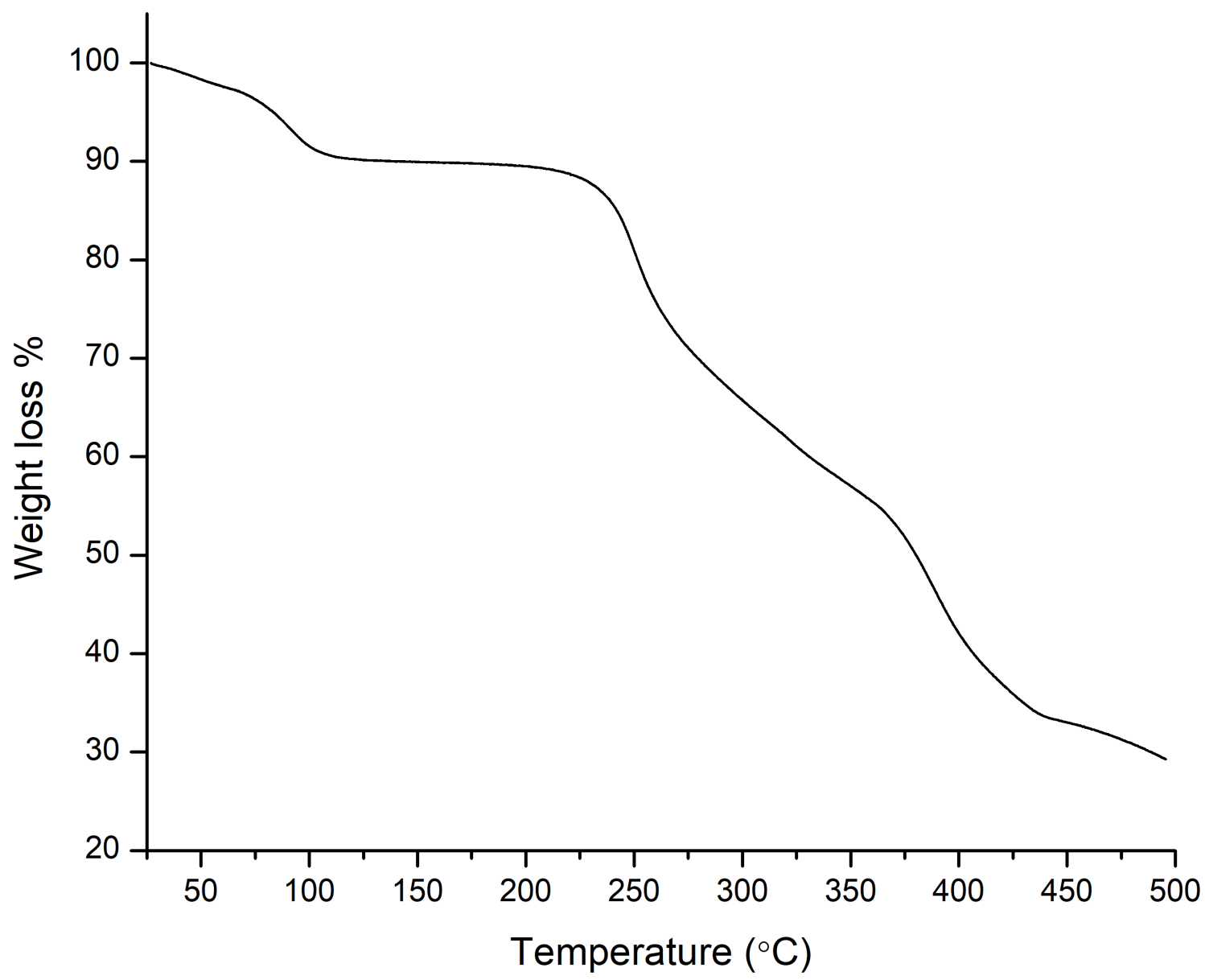

Figure S6.5: TGA thermogram of **Ni-Bzim-DL-mal**.

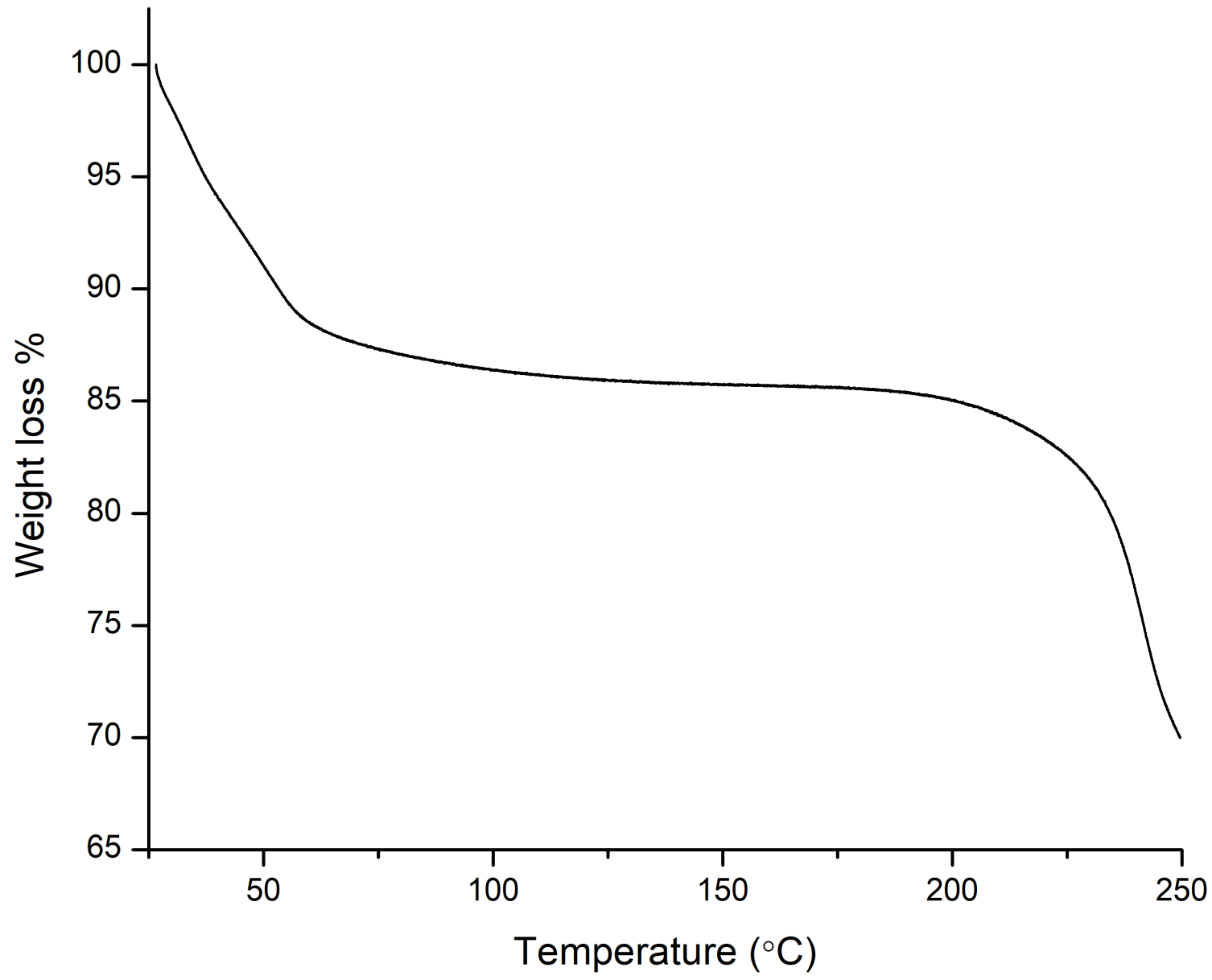

Figure S6.6: TGA thermogram of MeOH exchanged **Ni-Bzim-DL-mal**.

S7. Differential Scanning Calorimetry (DSC)

Differential scanning calorimetry was carried out using a TA Instruments Q2000 differential scanning calorimeter. Samples were prepared by crimping the sample pan and lid (a pin hole was placed in the lid to prevent pressure build-up). A reference pan was prepared in the same manner for each analysis. The sample and reference pans were heated at 10 °C min^{-1} from room temperature to 400 °C and so the heat flow, relative to the reference, was measured as a function of time and temperature under a controlled atmosphere. N_2 gas flowing at a rate of 40 mL min^{-1} was used to purge the furnace.

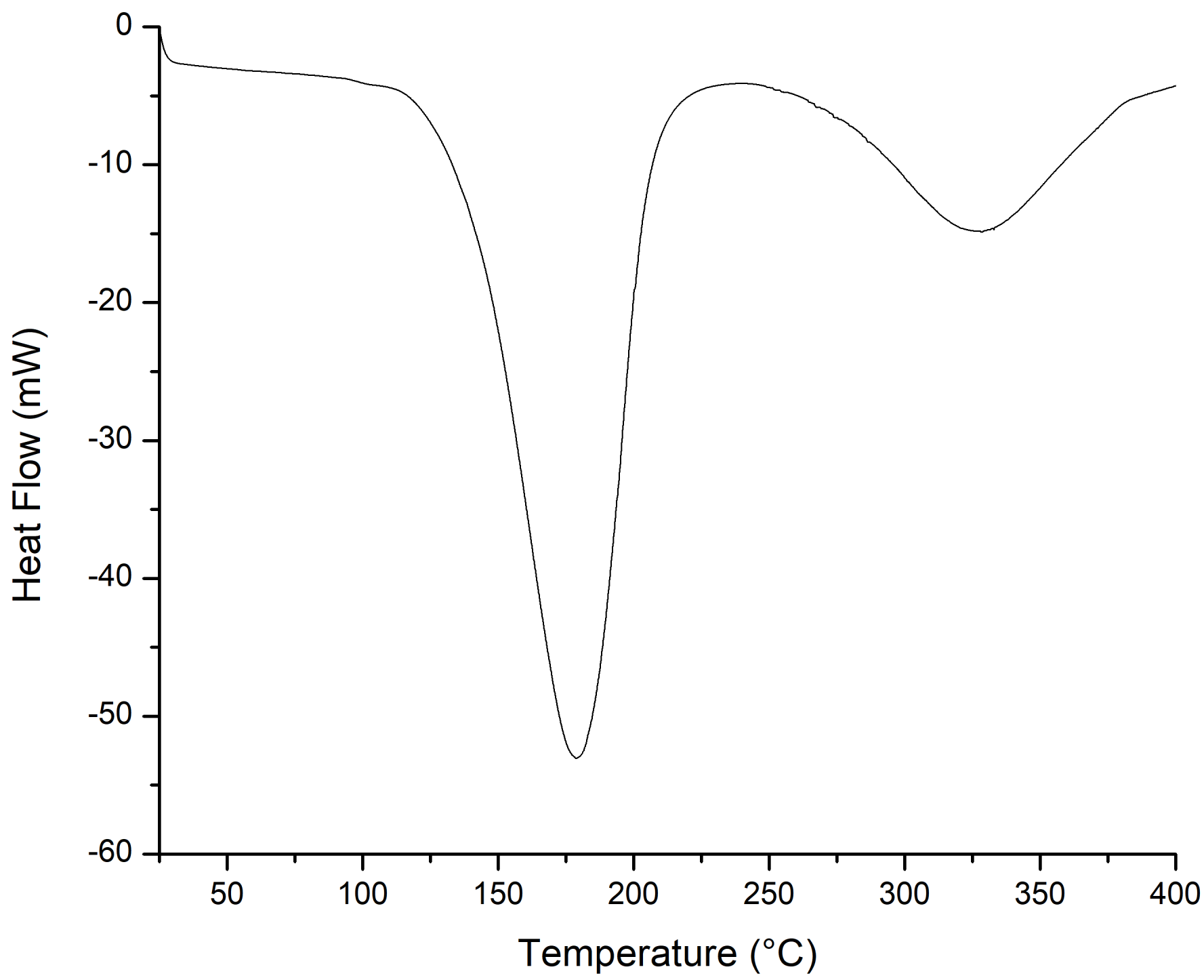

Figure S7.1: DSC thermogram of **Mg-Aqua-DL-mal**

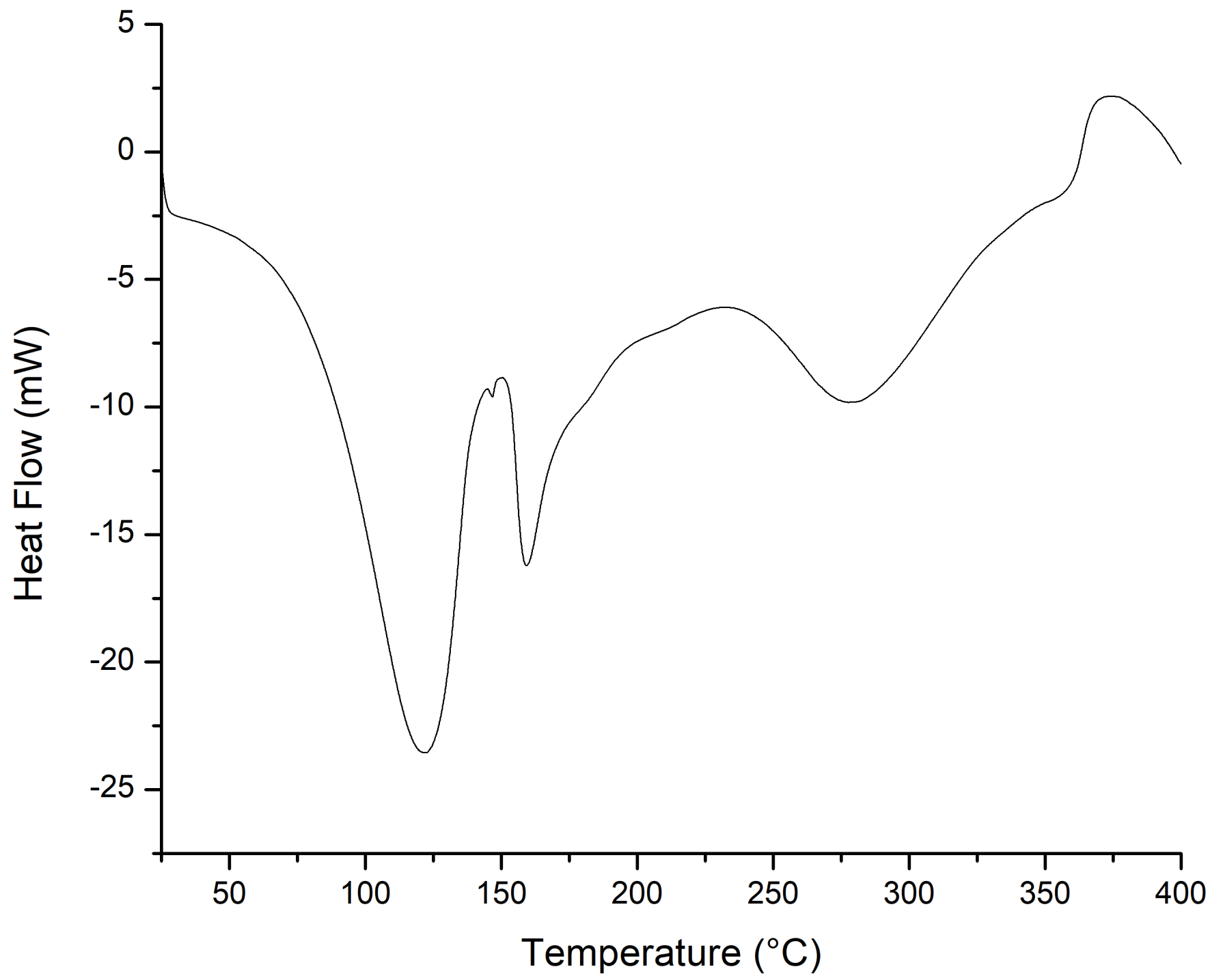

Figure S7.2: DSC thermogram of **Mn-Aqua-DL-mal**

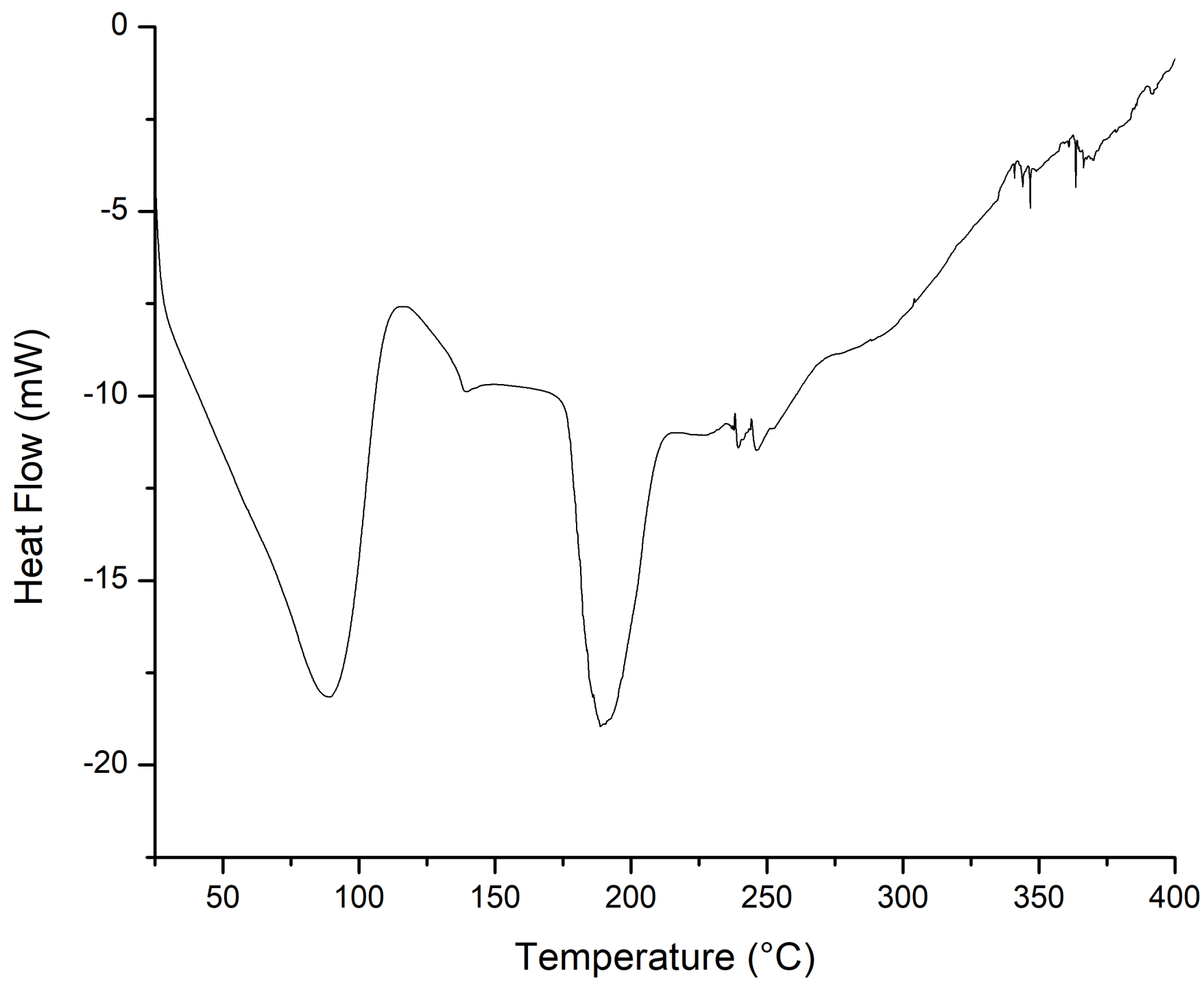

Figure S7.3: DSC thermogram of **Co-Bzim-DL-mal**

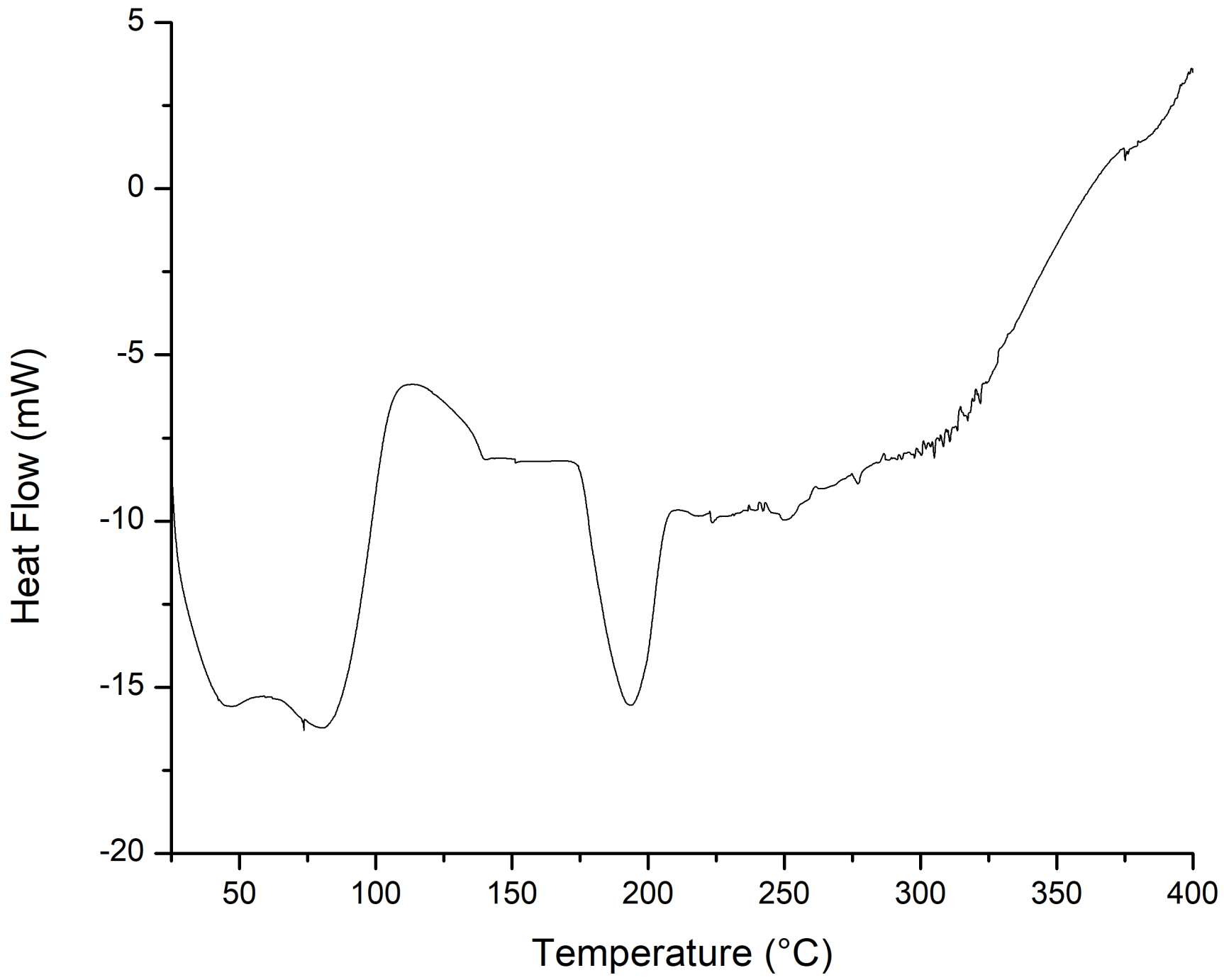

Figure S7.4: DSC thermogram of **Fe-Bzim-DL-mal**

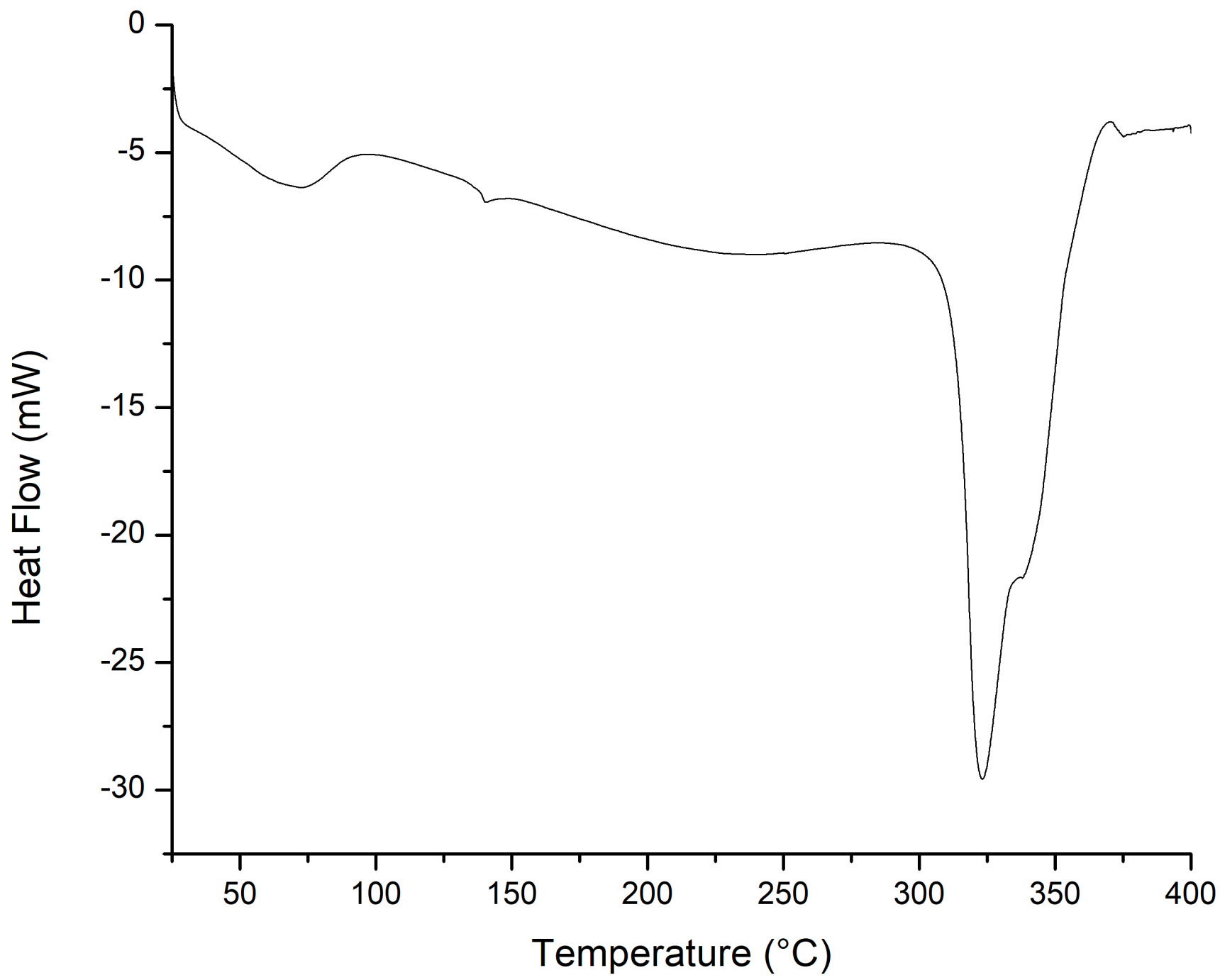

Figure S7.5: DSC thermogram of **Ni-Bzim-DL-mal**

S8. Fourier transform infrared

Fourier transform infrared (FTIR) spectra were collected on a PerkinElmer Spectrum 100 spectrometer with a Universal ATR accessory. The spectra for each sample was collected from 4000 to 500 cm^{-1}.

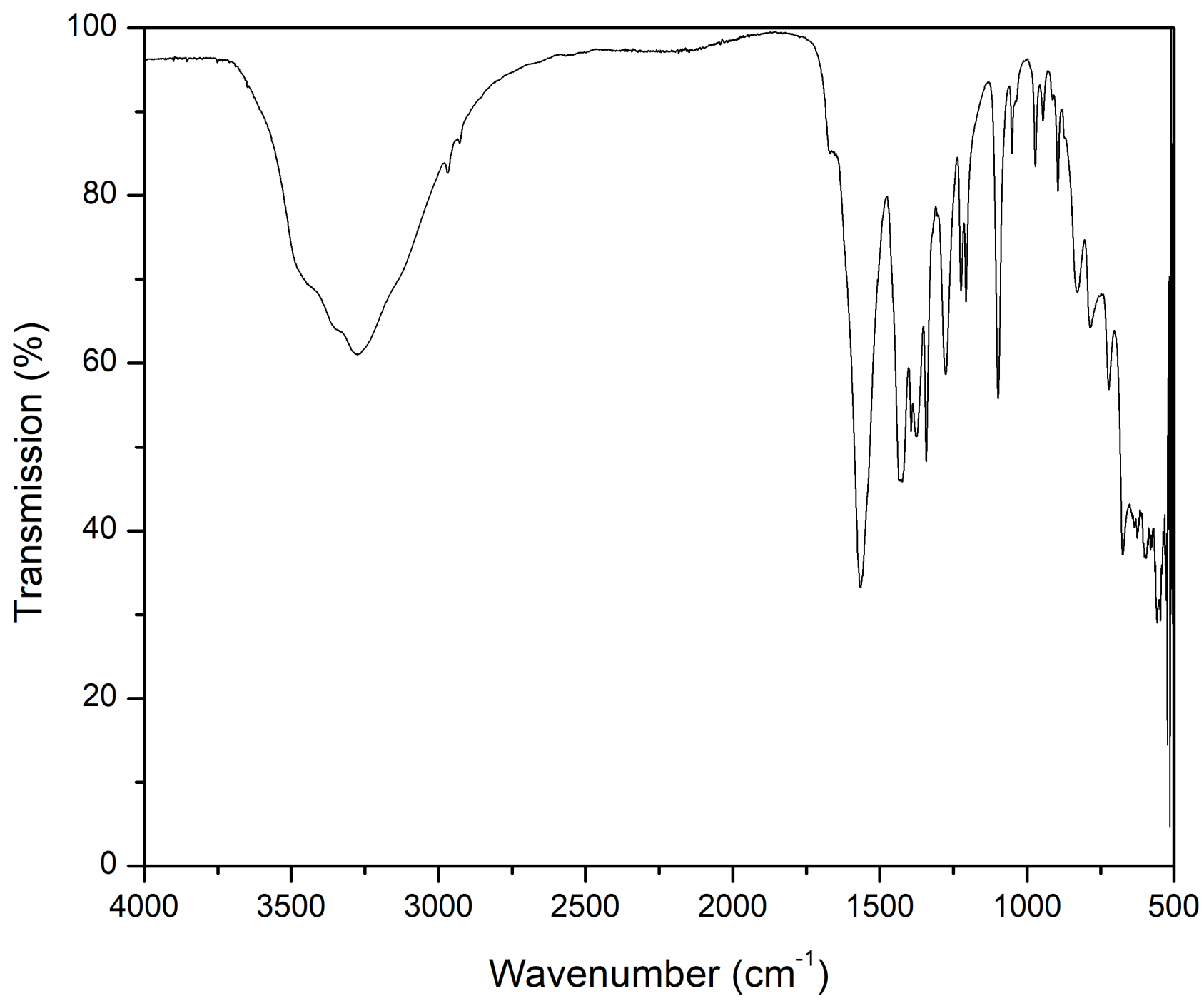

Figure S8.1: FTIR spectrum of **Mg-Aqua-DL-mal**

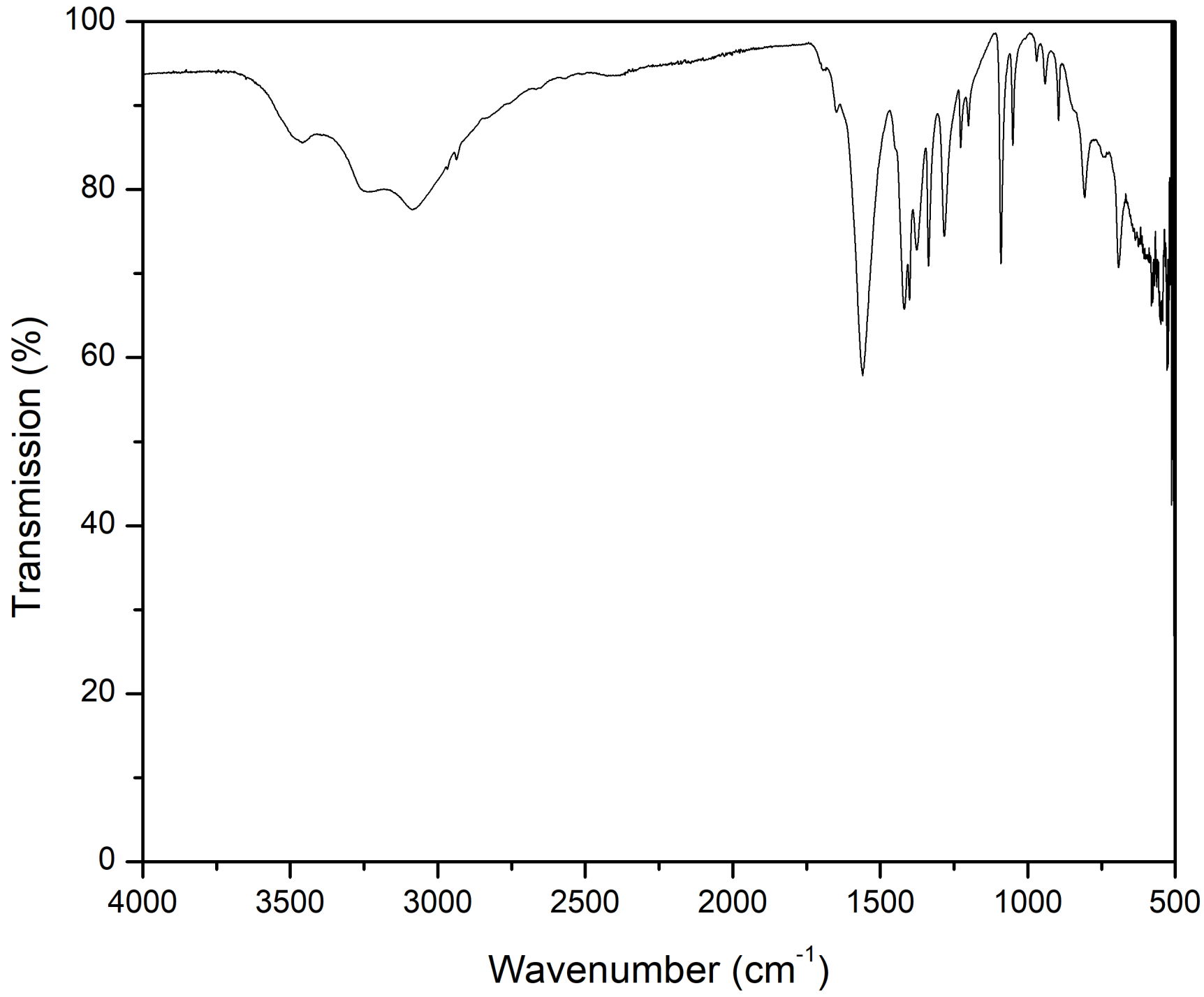

Figure S8.2: FTIR spectrum of **Mn-Aqua-DL-mal**

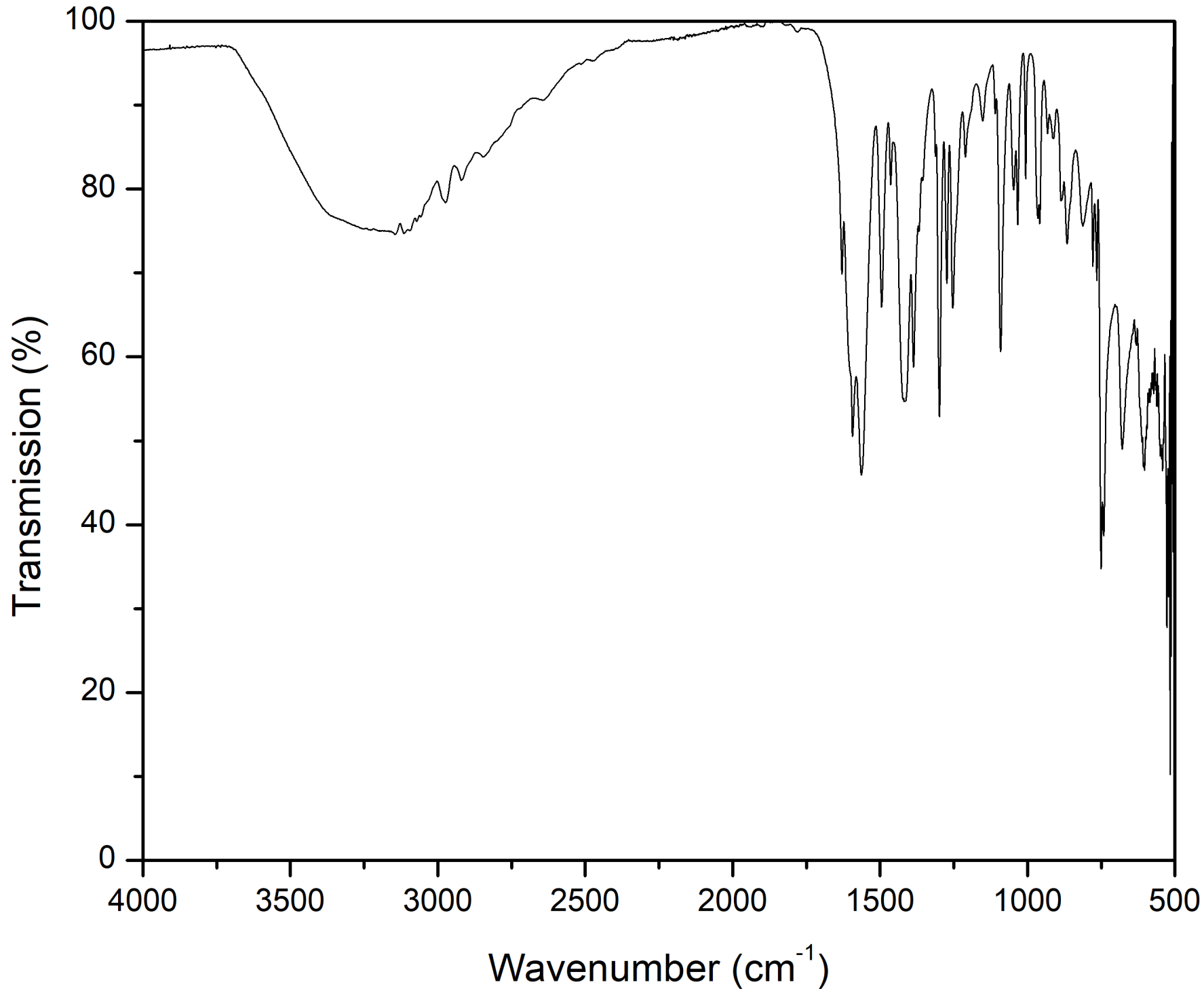

Figure S8.3: FTIR spectrum of **Fe-Bzim-DL-mal**

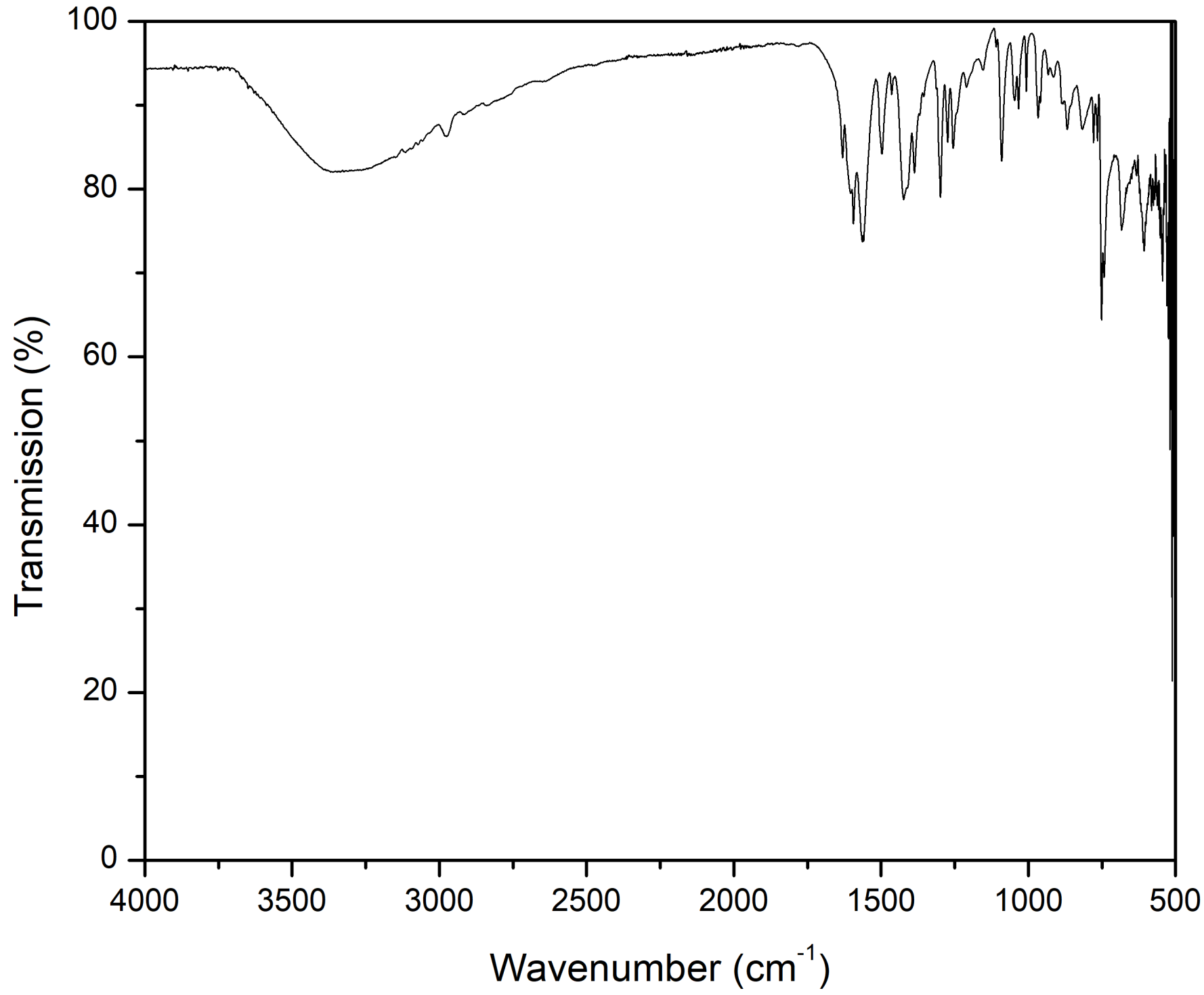

Figure S8.4: FTIR spectrum of **Co-Bzim-DL-mal**

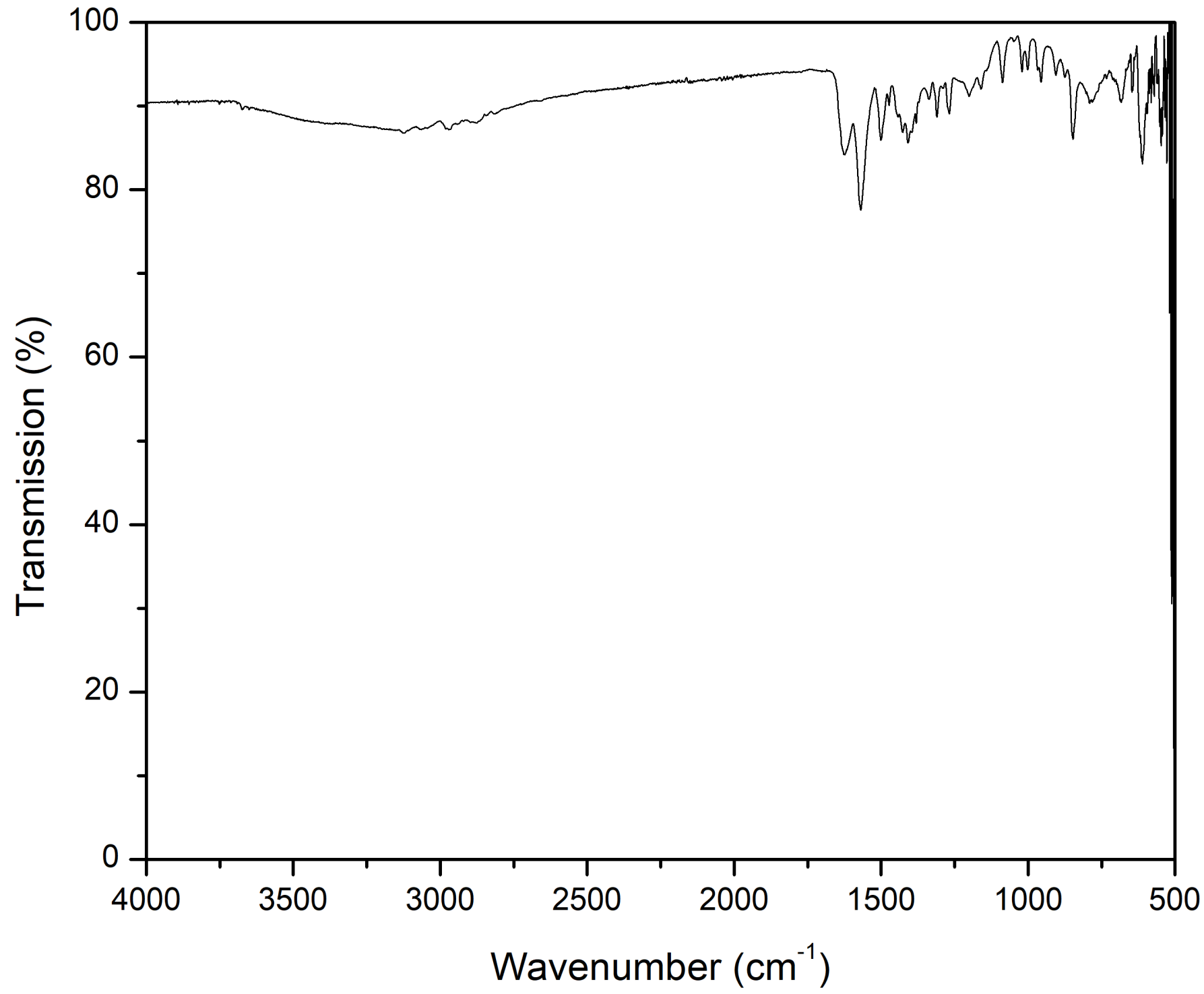

Figure S8.5: FTIR spectrum of **Ni-Bzim-DL-mal**

S9. Gas Adsorption

For gas sorption experiments, high-purity gases were used as received from BOC Gases Ireland: CO_2 (99.995%), N_2 (99.9995%%), and C_2H_2 (98.5%). A Micromeritics 3Flex surface area and pore size analyzer 3500 was used for collecting the 0 and 25 sorption isotherms of **Ni-Bzim-DL-mal**. Before sorption measurements, the **Ni-Bzim-DL-mal** was activated on a SmartVacPrep™ using dynamic vacuum at 60 °C, giving the porous guest-free **Ni-Bzim-DL-mal** (dry mass 70 mg). A Julabo temperature controller was used to maintain a constant temperature in the bath throughout the experiment.

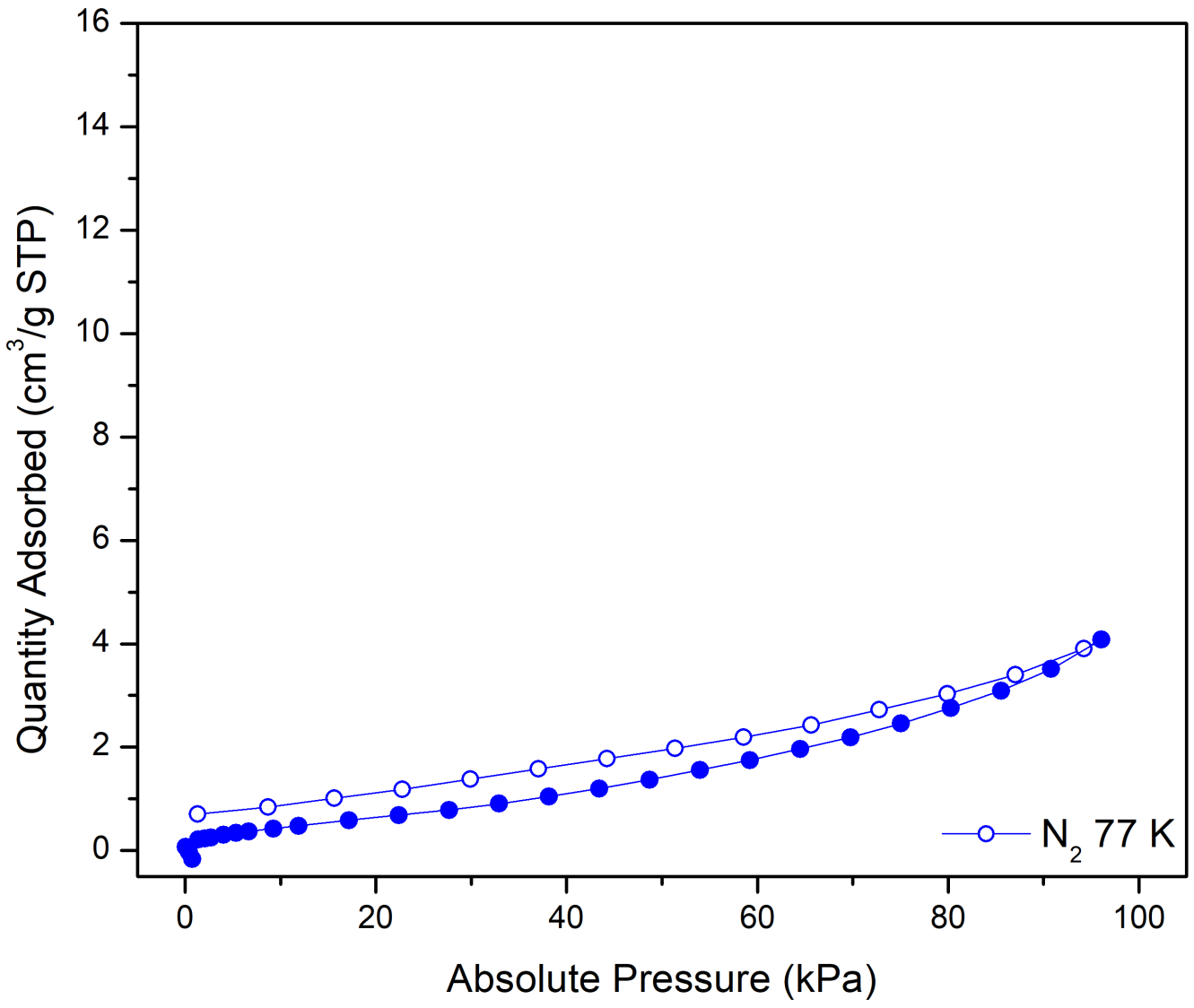

Figure S9.1: **Ni-Bzim-DL-mal** N_2 adsorption (filled circles) and desorption (hollow circles) isotherm.

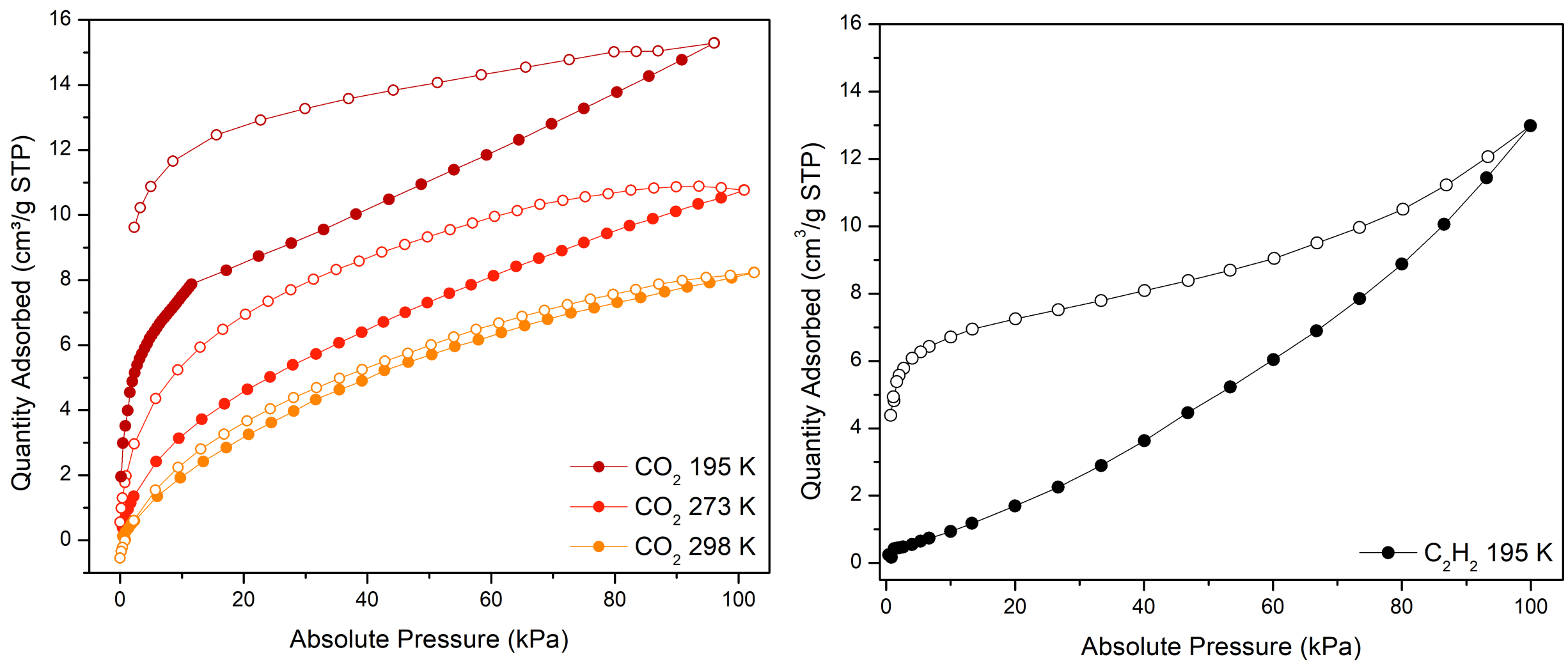

Figure S9.2: **Ni-Bzim-DL-mal** CO_2 (left) and C_2 (right) adsorption (filled circles) and desorption (hollow circles) isotherms.

S10. Water Vapor Sorption Isotherms

Water vapor sorption isotherm measurements were performed using Adventure dynamic vapor sorption (DVS) instrument manufactured by Surface Measurement Systems. The instrument gravimetrically measures water vapor uptake using air as a carrier gas. Digital mass flow controllers regulate flows of dry and saturated gases. Relative humidity is generated by precisely mixing dry and saturated gas flows in desired flow ratios which produce expected relative humidity. Pure water was used to generate water vapor for these measurements and temperature was maintained at 298 K by enclosing the system in a temperature-controlled incubator. The mass of the sample was determined by a high-resolution microbalance with a precision of 0.01 µg. Microbalance has symmetric configuration with two branches of the balance being exposed to the same gas and being kept at the same temperature, which allows negation of buoyancy and drag effects. Instrument allows measurement of 2 samples in parallel. Prior to the measurement, sample was in-situ activated in dry air at 333 K for four hours using built-in preheater and consequently cooled to sorption temperature. Isotherm measurements were performed on approximately 11 mg of sample powder. 400 sccm min^{-1} total flow was used for the measurements at 300 K and 50 sccm min^{-1} total flow was used for measurements at 333 K. The flow is split between two samples. For each isotherm point, dm/dt < 0.01 %/min was used as criteria of reaching equilibrium.

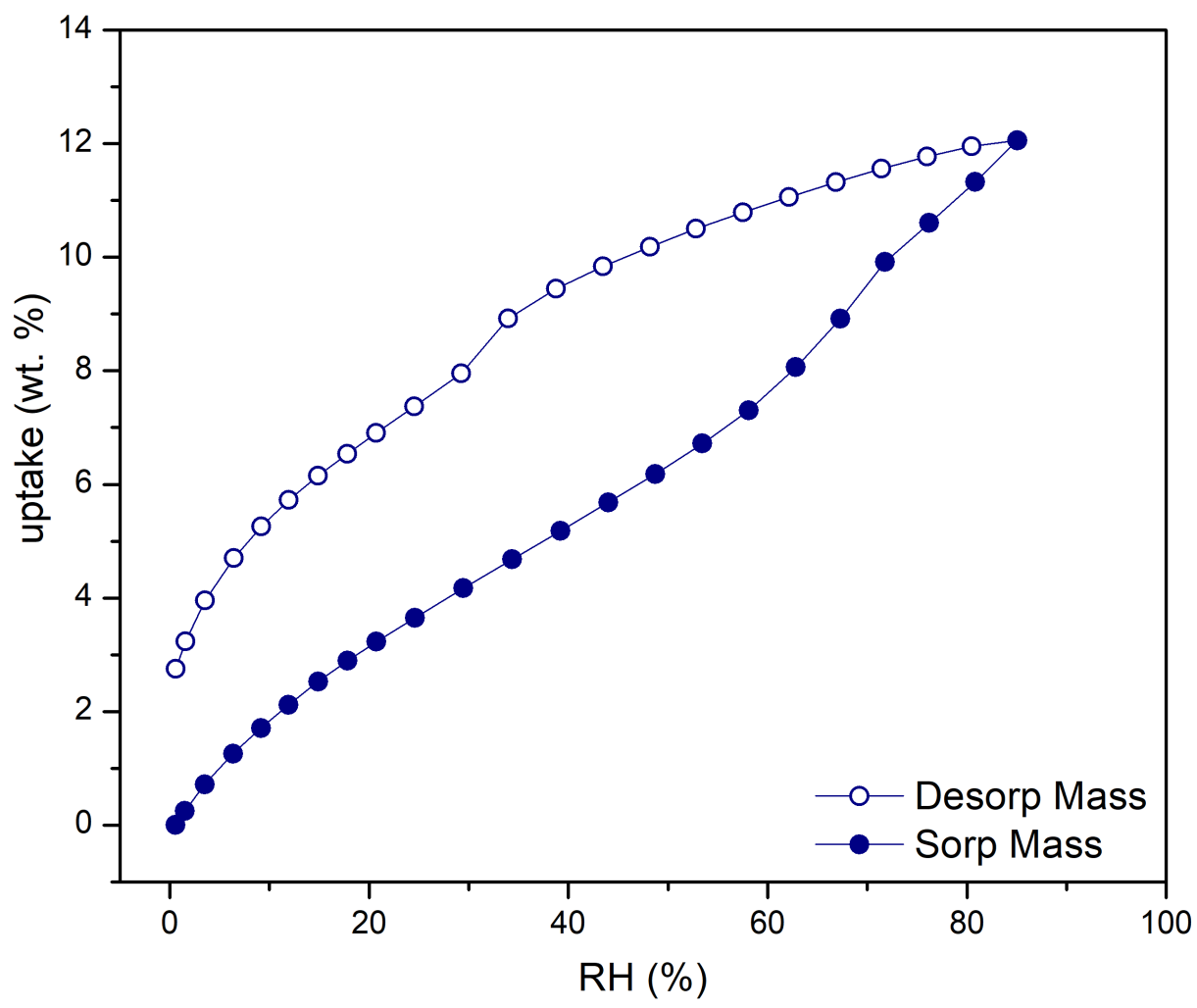

Figure S10.1: **Ni-Bzim-DL-mal** water vapour adsorption (filled circles) and desorption (hollow circles) isotherm collected at 25 °C.

S11. References

1. J.-H. Li, J.-J. Nie, J.-R. Su and D.-J. Xu, *Structure Reports*, 2008, **64**, m538-m539.
2. S. C. Ward and G. Sadiq, *CrystEngComm*, 2020, **22**, 7143-7144.
3. B. I. C. J. E. PR, *Pearson J. Taylor R. Acta Crystallogr., Sect. B: Struct. Sci*, 2002, **58**, 389-397.
4. C. F. Macrae, I. Sovago, S. J. Cottrell, P. T. Galek, P. McCabe, E. Pidcock, M. Platings, G. P. Shields, J. S. Stevens and M. Towler, *Applied Crystallography*, 2020, **53**, 226-235.
5. R. V. Gudavarthy, N. Burla, E. A. Kulp, S. J. Limmer, E. Sinn and J. A. Switzer, *J. Mater. Chem.*, 2011, **21**, 6209-6216.
6. B. Xia, Q. Gao, Z.-P. Hu, Q.-L. Wang, X.-W. Cao, W. Li, Y. Song and X.-H. Bu, *Research*, 2021.
7. G.-Y. D. G.-H.Cui, X.-H.Liu, Y.-M.Yu, *Zhurnal Strukturnoi Khimii*, 2010, **51**.

8. M. Fleck, E. Tillmanns, L. Bohatý and P. Held, *Zeitschrift für Kristallographie-Crystalline Materials*, 2004, **219**, 101-111.
9. A. Lenstra and J. Dillen, *Bulletin des Sociétés Chimiques Belges*, 1983, **92**, 257-262.
10. Y.-S. Song, B. Yan and Z.-X. Chen, *J. Solid State Chem.*, 2006, **179**, 4037-4046.
11. A. Beghidja, P. Rabu, G. Rogez and R. Welter, *Chem. Eur. J.*, 2006, **12**, 7627-7638.
12. X. Huang, F. Chen, H. Sun, L. Yang, Q. Yang, Z. Zhang, Y. Yang, Q. Ren and Z. Bao, *J. Am. Chem. Soc.*, 2023, **146**, 617-626.
13. Y.-L. Bai, J. Tao, R.-B. Huang and L.-S. Zheng, *CrystEngComm*, 2008, **10**, 472-474.
14. L. Kryger and S. Rasmussen, *Acta Chem. Scand*, 1972, **26**, 2349-2359.
15. W. Van Havere and A. Lenstra, *Bulletin des Sociétés Chimiques Belges*, 1978, **87**, 419-425.
16. H.-R. Fu, R.-Y. Zhang, D.-D. Ren, K. Zhang, J. Yuan, X.-Y. Lu and Q.-R. Ding, *Cryst. Growth Des.*, 2024, **24**, 4819-4824.
17. J. Zhang, S. Chen, A. Zingiryan and X. Bu, *J. Am. Chem. Soc.*, 2008, **130**, 17246-17247.
18. J.Kansikas, *Ann.Acad.Sci.Fenn.,Ser.A2:Chem*, 1985, **206**.
19. G.-C. Ou and S. W. Ng, *Structure Reports*, 2007, **63**, m2004-m2004.
20. A. Zingiryan, J. Zhang and X. Bu, *Inorg. Chem.*, 2008, **47**, 8607-8609.
21. E. Halevas, E. Chatzigeorgiou, S. Hadjispyrou, A. Hatzidimitriou, V. Psycharis and A. Salifoglou, *Polyhedron*, 2017, **127**, 420-431.
22. R.-H. Zhang, X.-W. Zhou and Z.-H. Zhou, *J. Coord. Chem.*, 2010, **63**, 3589-3598.
23. F. T. Xie, L. M. Duan, J. Q. Xu, L. Ye, Y. B. Liu, X. X. Hu and J. F. Song, *Eur. J. Inorg. Chem.*, 2004, **2004**, 4375-4379.
24. Y. Guo, D. Xiao, E. Wang, Y. Lu, J. Lü, X. Xu and L. Xu, *J. Solid State Chem.*, 2005, **178**, 776-781.
25. Q.-X. Liu, L.-B. Ni, F.-P. Liang, Z.-H. Zhou and H.-L. Wan, *Polyhedron*, 2009, **28**, 917-922.
26. R.-H. Zhang, Q.-M. Hong, J.-M. Yang, H.-L. Zhang, G. M. Blackburnb and Z.-H. Zhou, *Inorganica Chimica Acta*, 2009, **362**, 2643-2649.
27. M. S. Zavakhina, D. G. Samsonenko, A. V. Virovets, D. N. Dybtsev and V. P. Fedin, *J. Solid State Chem.*, 2014, **210**, 125-129.
28. D. Zhuravlev, I. Korolkov, M. Sokolov and P. Abramov, *J. Struct. Chem.*, 2025, **66**, 435-448.
29. M. M. R. C. V.H.Rodrigues, E.M.Gomes, A.Criado *Zeitschrift fur Kristallographie - New Crystal Structures*, 2006, **221**.
30. E. Mayorova, A. Pak, Y. V. Nelyubina and V. Novikov, *Russ. J. Coord. Chem*, 2023, **49**, 142-147.
31. K. D. Demadis, Z. Anagnostou and H. Zhao, *ACS applied materials & interfaces*, 2009, **1**, 35-38.
32. Y.-K. Lu, J. Liu, C.-L. Diao, R.-Q. Lü and Y.-Q. Liu, *Structure Reports*, 2011, **67**, m1684-m1685.
33. A. Devatine, J. P. Canselier, N. Gabas and J. Jaud, *J. Chem. Crystallogr.*, 2003, **33**, 85-90.
34. A. Beghidja, C. Beghidja, G. Rogez, P. Rabu and R. Welter, *Inorg. Chem. Commun.*, 2008, **11**, 1088-1090.
35. D. S. Raja, J.-H. Luo, T.-G. Chang, S.-H. Lo, C.-Y. Wu and C.-H. Lin, *Inorg. Chem. Commun.*, 2013, **32**, 22-27.
36. B. Liu, S. Dong, K. Lv, Z. Liu, S. Qi, S. Xiong and S. Hu, *J. Mater. Chem. A*, 2025, **13**, 12195-12202.
37. L.-P. Zhang, L. Huang, L.-b. Qu, H. Peng and Y.-F. Zhao, *J. Mol. Struct.*, 2006, **787**, 14-17.
38. J.-Q. Liu, Y.-Y. Wang, P. Liu, W.-P. Wu, Y.-P. Wu, X.-R. Zeng, F. Zhong and Q.-Z. Shi, *Inorg. Chem. Commun.*, 2007, **10**, 343-347.
39. A. Karipides and A. T. Reed, *Inorg. Chem.*, 1976, **15**, 44-47.
40. A. T. Reed and A. Karipides, *Structural Science*, 1976, **32**, 2085-2088.

41. R. Carballo, B. Covelo, N. Fernández-Hermida, E. García-Martínez, A. B. Lago, M. Vázquez and E. M. Vázquez-López, *Cryst. Growth Des.*, 2006, **6**, 629-631.
42. M. Fleck, E. Tillmanns and L. Bohatý, *Zeitschrift für Kristallographie-Crystalline Materials*, 2001, **216**, 633-645.
43. S. Sushrutha and S. Natarajan, *CrystEngComm*, 2014, **16**, 4774-4782.
44. J.-Q. Liu, Y.-Y. Wang, L.-F. Ma, W.-H. Zhang, X.-R. Zeng, Q.-Z. Shi and S.-M. Peng, *Inorganica Chimica Acta*, 2008, **361**, 2327-2334.
45. X. He, C.-Z. Lu and Y. Yan, *Inorg. Chem. Commun.*, 2004, **7**, 851-853.
46. Y. Jian, T. Jia-Yu, X. Guo-Xiang, M. Ting, Y. Xue-Mei, D. Li-Ting, L. Xu and X. Hai-Jun, *Chin. J. Inorg. Chem.*, 2018, **34**, 1312-1318.
47. A. Ghosh and C. Rao, *Zeitschrift für anorganische und allgemeine Chemie*, 2008, **634**, 1115-1122.
48. C. Lei, Z. Wang, F. Zhang, Z. Yang, F. Zhang, Y. Wang and S. Pan, *Inorg. Chem. Commun.*, 2015, **61**, 5-9.
49. H. H. M. Yeung, W. Li, P. J. Saines, T. K. Köster, C. P. Grey and A. K. Cheetham, *Angew. Chem. Int. Ed.*, 2013, **52**.
50. A. Lincy, V. Mahalakshmi, J. Thomas and K. Saban, *IOSR J. Appl. Phys.*, 2014, **6**, 56-61.
51. A. Beghidja, G. Rogez, P. Rabu, R. Welter and M. Drillon, *J. Mater. Chem.*, 2006, **16**, 2715-2728.
52. R. Wartchow, *Zeitschrift für Kristallographie-Crystalline Materials*, 1996, **211**, 329-330.
53. Z.-H. Zhou, J.-J. Ye, Y.-F. Deng, G. Wang, J.-X. Gao and H.-L. Wan, *Polyhedron*, 2002, **21**, 787-790.
54. H.-Y. Bai, J.-F. Ma, Y.-Y. Liu and J. Yang, *Inorganica Chimica Acta*, 2011, **376**, 332-339.
55. M. Teusner, J. Mata, B. Johannessen, G. Stewart, S. Cadogan and N. Sharma, *Mater. Adv.*, 2023, **4**, 3224-3238.
56. T.-F. Zheng, X.-M. Tian, L.-H. Wu, S.-J. Liu, S.-L. Yao, K.-H. He, H. Huang, J. Liao, J.-L. Chen and H.-R. Wen, *New Journal of Chemistry*, 2019, **43**, 18445-18450.
57. M. P. Yutkin, M. S. Zavakhina, D. G. Samsonenko, D. N. Dybtsev and V. P. Fedin, *Inorganica Chimica Acta*, 2013, **394**, 367-372.
58. L. Zhu and F. Sun, *Structure Reports*, 2007, **63**, m2966-m2966.
59. W. Wu, J. Liu, F. Zeng and Y. Wu, *Crystal Research and Technology: Journal of Experimental and Industrial Crystallography*, 2008, **43**, 991-994.
60. A. B. Peraka Krishna, Ewa Patyk-Kaźmierczak, Michał Kaźmierczak, Daniel O'Nolan, Alcemira C. Oliveira, Miranda Perry, Michael J. Zaworotko,, *CSD Communication*, 2024.
61. T. B. Kinraide and U. Yermiyahu, *Journal of inorganic biochemistry*, 2007, **101**, 1201-1213.
62. G. Sheldrick, *Acta Cryst. A*, 2007, **64**, 112-122.
63. B. SMART, *Bruker AXS, Madison, Wisconsin (USA)*, 2000.
64. G. Sheldrick, *Struct. Chem.*, 2015, **71**, 3.
65. O. V. Dolomanov, L. J. Bourhis, R. J. Gildea, J. A. Howard and H. Puschmann, *Applied Crystallography*, 2009, **42**, 339-341.
66. G. M. Sheldrick, *Acta Crystallographica Section A: Foundations and Advances*, 2015, **71**, 3-8.
67. C. F. Macrae, I. Sovago, S. J. Cottrell, P. T. Galek, P. McCabe, E. Pidcock, M. Platings, G. P. Shields, J. S. Stevens and M. Towler, *Journal of applied crystallography*, 2020, **53**, 226-235.
